# Development of a supramolecular solvent–based extraction method for application to quantitative analyses of a wide range of organic contaminants in indoor dust

**DOI:** 10.1007/s00216-024-05433-3

**Published:** 2024-07-12

**Authors:** Paula Marcinekova, Lisa Melymuk, Pernilla Bohlin-Nizzetto, Erika Martinelli, Simona Rozárka Jílková, Jakub Martiník, Petr Šenk, Petr Kukučka, Ondřej Audy, Jiří Kohoutek, Mebrat Ghebremeskel, Alexander Håland, Anders Røsrud Borgen, Heidi Eikenes, Linda Hanssen, Mikael Harju, Zofia Cebula, Pawel Rostkowski

**Affiliations:** 1 RECETOX, Faculty of Science, Masaryk University, Kotlářská 2, 61137 Brno, Czechia; 2 NILU, Instituttveien 18, Kjeller, 2007 Lillestrøm, Norway; 3 Fram Center, NILU, Hjalmar Johansens Gate 14, 9007 Tromsø, Norway; 4 Institute of Biotechnology and Molecular Medicine, Kampinoska 25, 80-180, Gdańsk, Poland

**Keywords:** SUPRAS, Indoor environments, Plastic additives, Pesticides, PFAS

## Abstract

This study investigates the efficacy of supramolecular solvent (SUPRAS) in extracting a diverse spectrum of organic contaminants from indoor dust. Initially, seven distinct SUPRAS were assessed across nine categories of contaminants to identify the most effective one. A SUPRAS comprising Milli-Q water, tetrahydrofuran, and hexanol in a 70:20:10 ratio, respectively, demonstrated the best extraction performance and was employed for testing a wider array of organic contaminants. Furthermore, we applied the selected SUPRAS for the extraction of organic compounds from the NIST Standard Reference Material (SRM) 2585. In parallel, we performed the extraction of NIST SRM 2585 with conventional extraction methods using hexane:acetone (1:1) for non-polar contaminants and methanol (100%) extraction for polar contaminants. Analysis from two independent laboratories (in Norway and the Czech Republic) demonstrated the viability of SUPRAS for the simultaneous extraction of twelve groups of organic contaminants with a broad range of physico-chemical properties including plastic additives, pesticides, and combustion by-products. However, caution is advised when employing SUPRAS for highly polar contaminants like current-use pesticides or volatile substances like naphthalene.

**Supplementary Information:**

The online version contains supplementary material available at 10.1007/s00216-024-05433-3.

## Introduction

The requirements for the preparation of biological and environmental samples have, over time, increased to meet the demands of the analytical instruments. Many research disciplines, including environmental monitoring, epidemiological studies, exposomics, forensics, and anti-doping control, have shifted their focus from single chemical analysis to suspect and or wide-scope target screening and detection of structurally unrelated multiclass chemicals [1–4]. However, conventional liquid–liquid and solid-phase extraction methods are often tailored to specific pollutants and may not be suitable for the extraction of a broad range of pollutants [5]. Additionally, they are often labour-intensive and time-consuming and use large quantities of organic solvents contributing to the generation of high amounts of chemical waste [1, 5–7]. Conventional methods for sample preparation are progressively being replaced by novel approaches. These involve strategies like miniaturization aimed to diminish solvent consumption [2, 8], improved solvent extraction to enhance extraction efficiency [2, 9], and the adoption of environmentally friendly solvents [2, 8].

Supramolecular solvents (SUPRAS) are nanostructured water-immiscible liquids resulting from the self-assembly and coacervation of amphiphiles in colloidal suspensions [8, 10]. In this two-step process, a colloidal suspension of amphiphiles is first spontaneously formed on a molecular level. It contains three-dimensional supramolecular aggregates such as aqueous or reverse micelles or vesicles. The colloidal suspension then self-assembles on a nanoscale level via coacervation, a type of liquid–liquid phase separation typically occurring in colloidal solutions [8, 10, 11]. The environmental conditions of the colloidal suspension are altered by a coacervation-inducing agent (poor amphiphile solvent, pH or temperature change, or addition of salt) to promote coacervation of the aggregates. The amphiphile concentration present in SUPRAS ranges from 0.1 to 1 mg/µl, offering an abundance of hydrophilic and hydrophobic binding sites. The large number of binding sites facilitates an efficient extraction of compounds at very low solvent volumes [10, 12–14]. The extraction efficiency is further enhanced by the ability of SUPRAS to extract compounds across a broad range of polarities (both polar and non-polar). This property arises from the presence of a high number of different polarity microenvironments in the supramolecular aggregates [13, 15–17]. The multiple extraction mechanisms offered by SUPRAS, including dispersion, hydrogen bonding, polar, ionic, and dipole–dipole interactions, further amplify the extraction efficiency [10, 13, 15, 18]. As a result, extraction with SUPRAS is particularly useful for suspect screening or non-targeted analyses, where a broad range of contaminants needs to be extracted [3, 8, 10]. Besides its significant extraction potential, SUPRAS also exhibit restricted access properties that efficiently remove undesired matrix components (proteins, carbohydrates, lipids, humic acids) throughout the extraction process, reducing or eliminating the need for additional cleanup steps [17, 19, 20]. There are also noteworthy operational characteristics of SUPRAS that make them excellent substitutes to conventional extraction methods: SUPRAS are non-flammable, low in volatility and toxicity, and the synthesis of SUPRAS follows a simple, time-efficient, safe, and environmentally sound process that aligns with the principles of green chemistry [10, 13, 17, 20].

SUPRAS have primarily been utilized for the extraction of environmental pollutants that are commonly analysed by liquid chromatography-mass spectrometry (LC–MS) [2, 17]. This is because the SUPRAS extracts can be injected directly into the liquid chromatograph unless they exhibit significant viscosity, and highly viscous extracts can be diluted with an organic solvent. The prevalent system used is reversed-phase LC, but SUPRAS extracts are also compatible with micellar and chiral LC [2]. Most recently, the use of SUPRAS with advanced instrumental screening has been demonstrated through the use in combined target, suspect, and non-target screening of chemicals in indoor dust, identifying 146 compounds using LC–MS [17]. The combination of SUPRAS extracts with gas chromatography (GC) presents more challenges compared to LC, mainly because of the extract’s viscous nature, the low volatility, and the high concentration of the surfactants. Direct injection of SUPRAS extracts is not feasible due to the potential damage they could cause to the GC injector and capillary column. Several approaches have been suggested to enhance the compatibility of SUPRAS extracts with GC. These approaches predominantly involve the elimination of surfactants, often accomplished through solid-phase extraction or back-extraction techniques prior to injecting the extract into the GC system [2, 8, 21]. In a more recent development, Takagai and Hinze have proposed the use of derivatization to eliminate the necessity of surfactant removal [22]. This innovative approach not only enhances chromatographic performance but also establishes a favourable elution time window for analytes, ensures consistent retention times, and enables precise quantification of results [2, 22]. The use of headspace GC techniques has also been proposed [23]; however, this approach faces some criticism due to concerns that the heating of SUPRAS could lead to the production of substantial amounts of organic vapours and decomposition by-products [21]. SUPRAS have proven effective in capturing substances such as polycyclic aromatic compounds (PACs), pesticides, bioactive compounds, dyes, endocrine disruptors, phenols, surfactants, and other organic pollutants. The source matrices encompass a wide range, including soil, sediments, various water types (seawater, wastewater, tap water, lake water, snow), sewage sludge, foods (such as fruits, vegetables, canned products, fish), beverages (like tea, coffee, and beer), as well as human urine and plasma samples [8]. However, despite having the potential to extract a wide range of contaminants, SUPRAS are usually employed for the extraction of a single group of contaminants that share similar physico-chemical properties [10, 12, 14, 15, 18, 24–27]. Although there have been some studies employing SUPRAS extraction for quantitative analysis of multiclass substances [3, 13, 17], the use of such approaches remains limited.

Indoor settled dust receives substantial attention as a complex matrix with relevance to human exposure and is often used to identify chemical sources relevant to indoor environments [28, 29]. While many groups of chemicals have been reported in indoor dust [29], typically one or a few selected chemical groups are quantitatively characterized in individual dust extracts, and broad characterization can require multiple extractions [30].

The objective of this study was to investigate the capability of SUPRAS to simultaneously extract multiple classes of chemically and structurally unrelated organic contaminants from indoor settled dust with recoveries sufficient for quantitative analyses with GC- and LC-MS. For this purpose, nine classes of organic contaminants, reflecting a range of contaminants of concern, were initially selected: polycyclic aromatic hydrocarbons (PAHs), substituted PAHs (oxy- and nitro-PAHs), organochlorine pesticides (OCPs), polychlorinated biphenyls (PCBs), synthetic polycyclic musks, phthalate esters (PTHTs), per- and polyfluoroalkyl substances (PFAS), current-use pesticides (CUPs), and legacy and novel flame retardants (FRs). Seven different SUPRAS were tested. A SUPRAS demonstrating the highest extraction efficiency was selected and applied for testing the efficiency of extraction of additional classes of organic contaminants, including hexabromocyclododecane (HBCD), parabens, bisphenols and derivatives of bisphenol A and F (BADGE/BFGDE), organophosphate flame retardants (OPFRs), chlorinated paraffins (CPs), and dechloranes.

## Methods

### Chemicals

Tetrahydrofuran (THF) and 1-decanol were acquired from Sigma-Aldrich (Germany). Hexanol was purchased from Thermo Fisher Scientific (Germany), while ethanol was purchased from VWR Chemicals (France). Methanol (MeOH) was obtained from Biosolve Chimie (France), and acetone and dichloromethane (DCM) were obtained from Fisher Scientific (UK). Hexane was purchased from Avantor-J.T.Baker (Poland). All chemicals were of analytical reagent quality. The ultrapure water was obtained from the Sartorius Arium® Mini (Germany) Milli-Q water purification system. The complete list of isotopically labelled or deuterated standards, further referred to as surrogates (added to the sample prior extraction) and internal standards (added prior to injection), used in this study can be found in Tables S1 and S2 in SI.

### Dust collection and preparation

Settled indoor dust samples were collected from fifteen homes in the South Moravian region of the Czech Republic in summer 2022. Prior to sampling, glass Petri dishes were thermally sterilized and pre-weighed quartz filters (Whatman QM-A Quartz Microfiber, 101.6 mm, 2.2 μm) were autoclaved using a Tuttnauer 3850EL-D (Netherlands) autoclave. A household vacuum cleaner equipped with a modified sampling head that allowed for the collection of particles < 1 mm onto the quartz fibre filter (QFF) was used to collect the dust samples. The sampling head was pre-cleaned using 95% ethanol before each sampling. A composite dust sample was collected from the “most frequently used” room of the house, which was typically a living room, or a living room connected to a kitchen. Following sampling, the QFF with the dust sample was packed into the Petri dish, sealed with parafilm, placed into a zip-lock bag, and stored in a freezer at − 18 °C until further processing.

The dust samples, along with the QFF, were pulverized using a Retsch MM 301 (Germany) mixer mill equipped with ZrO_2_ cartridges. Subsequently, the resulting ground sample was transferred to a pre-weighed sterile glass vial. To ensure sufficient material for testing, all fifteen samples were combined to create a pooled sample, which was then used in phase 1 and phase 2 evaluations.

### SUPRAS synthesis

SUPRAS were prepared with reverse aggregates of short- and long-chain alcohols (hexanol and decanol, respectively) in mixtures of water as the coacervating agent and THF as the organic solvent. By varying the composition of the bulk solution (THF:water:hexanol/decanol ratio), seven different SUPRAS were synthesized. We based our study on a previously published study by Caballero-Casero and Rubio [15], where SUPRAS of reverse aggregates was successfully utilized for extraction of bisphenols from dust samples. The SUPRAS and the volume of each SUPRAS component utilized are presented in Table 1. Prior to use, THF was purified with aluminium oxide. Seven sterilized 50-ml centrifuge tubes were filled with the SUPRAS components according to Table 1. These were then manually shaken and centrifuged for 30 min at 5 °C at 2400 rpm. The upper amphiphile-rich SUPRAS phase was separated and transferred to a sterilized 15-ml centrifuge tube. The remaining equilibrium solution phase was retained. Both phases were refrigerated at 4 °C and used within 1 month of synthesis.

**Table 1:** SUPRAS and their components, tested under phase 1

SUPRAS	Milli-Q water	Organic solvent	Amphiphile	Ratio
1	21 ml	6 ml THF	3 ml hexanol	70:20:10
2	19.5 ml	6 ml THF	4.5 ml hexanol	65:20:15
3	24 ml	3 ml THF	3 ml hexanol	80:10:10
4	15 ml	12 ml THF	3 ml hexanol	50:40:10
5	9 ml	18 ml THF	3 ml hexanol	30:60:10
6	25.5 ml	3 ml ethanol	1.5 ml decanol	85:10:5
7	19.5 ml	9 ml ethanol	1.5 ml decanol	65:30:5

### Extraction

The testing and validation of the SUPRAS methodology involved three phases. The full list of compounds and associated abbreviations for each phase are given in Table S3.

In phase 1, the pooled dust sample was split and extracted using the seven different SUPRAS to identify the most effective SUPRAS for predetermined groups of organic pollutants: PAHs and substituted PAHs, OCPs, PCBs, musks, PTHTs, PFAS, CUPs and FRs, including polybrominated diphenyl ethers (PBDEs) and novel flame retardants (NFRs) (Table S3). This phase of the study took place at RECETOX, Masaryk University, in Czech Republic.

In phase 2, the subsequent extraction of the same pooled dust sample was performed at NILU in Norway. The selected SUPRAS from step 1 was assessed for its efficacy in extracting a wider scope of pollutants, including HBCD, OPFRs, parabens, bisphenols, BADGE/BFGDE, CPs, and dechloranes (Table S3).

In phase 3, the SUPRAS extraction was performed in parallel with “conventional” hexane:acetone (hex:acet) and MeOH extractions [30–34] to extract a set of non-polar and polar contaminants, respectively, from the NIST standard reference material (SRM) 2585: Organic contaminants in house dust. This third and final phase of extractions took place at RECETOX.

**Fig. 1:**
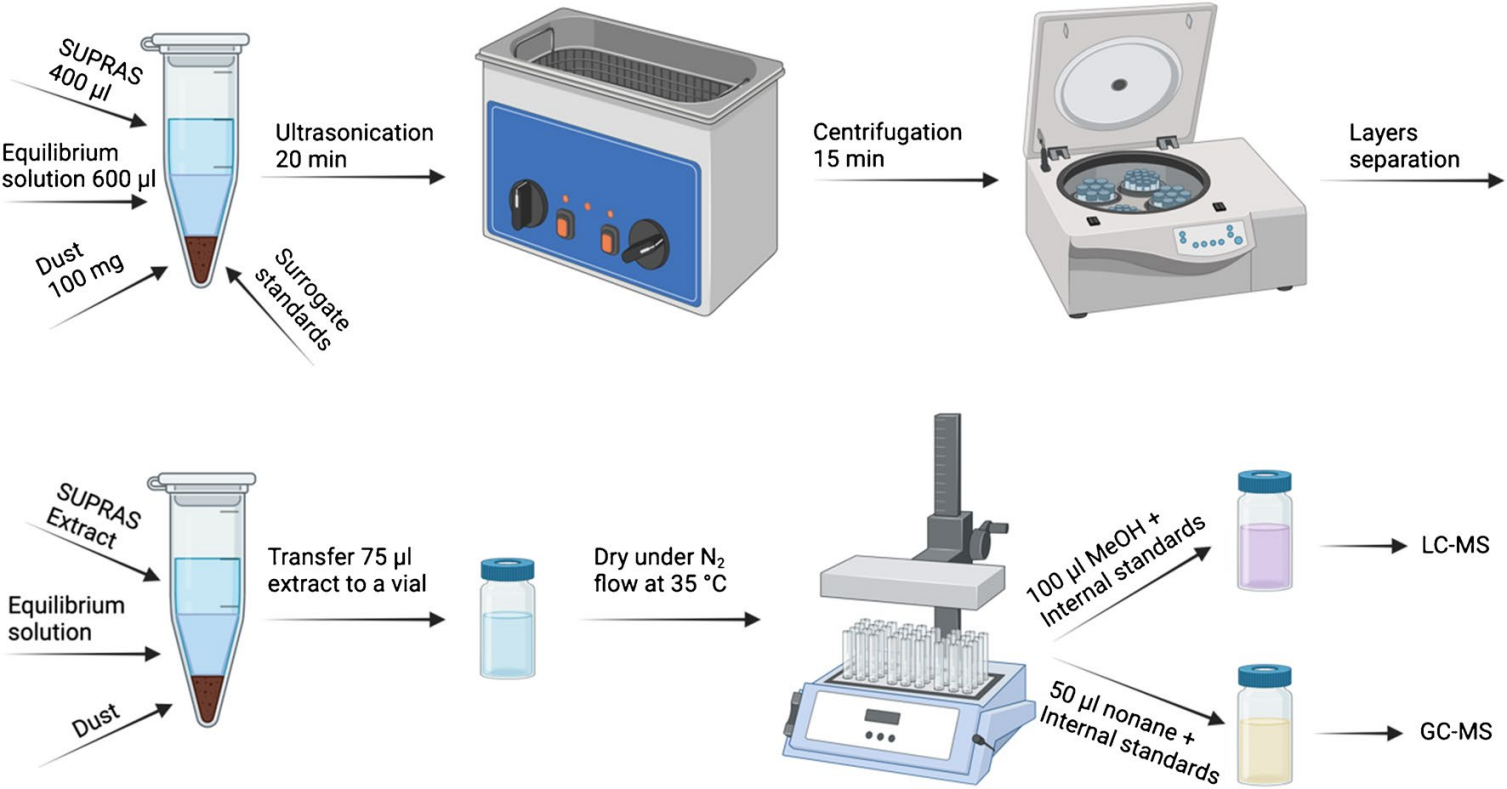
Schematic extraction of dust with SUPRAS

In each phase, a mass of 100 mg of the pooled dust sample or SRM 2585 was weighed and transferred to 2-ml Eppendorf tubes. The dust samples were spiked with surrogate standards and subsequently evaporated under a controlled nitrogen (N_2_) flow at 35 °C. Next, the dried samples were hydrated using 600 µl of SUPRAS equilibrium solution and then extracted with 400 µl of SUPRAS. The extraction was carried out using ultrasonication for 20 min at room temperature (RT), followed by centrifugation at 11,000 rpm for 15 min at RT to ensure complete phase separation. The obtained SUPRAS extract was split into four 1-ml glass vials, each containing 75 µl of the extract and again evaporated under controlled N_2_ flow at 35 °C. Subsequently, the analytes were dissolved in a solvent compatible with either LC–MS or GC–MS analysis, as shown in Fig. 1.

In phase 3, extraction with hex:acet (1:1 v/v) was performed to isolate non-polar (PAHs, NFRs, PHTHs, PBDEs, PCBs, OCPs) while extraction with MeOH (100%) was performed to isolate polar (CUPs, PFAS) contaminants from the pooled dust samples. The extraction methods used have been previously published [35–37] and are briefly described here. For the non-polar contaminants, the dust samples/SRMs were sonicated three times in hex:acet, and extracts were split 70:30. The 70% aliquot was cleaned and fractionated using H_2_SO_4_ silica column eluted with hexane:DCM (1:1 v/v). The 30% aliquot was cleaned and fractionated using an activated silica column eluted with hexane (1st fraction), followed by DCM (2nd fraction). The fractionated extracts were exchanged to 50 µl nonane. For the polar contaminants, the dust samples/SRMs were sonicated three times in MeOH and cleaned by filtration through a nylon filter. The extracts were partially evaporated under N_2_ flow and dissolved in 0.5 ml MeOH.

### Analysis

The instrumental analyses were conducted at accredited laboratories at RECETOX, Masaryk University, in the Czech Republic and NILU, in Norway. The RECETOX laboratory analysed samples derived from phase 1 (involving seven SUPRAS for testing) and phase 3 (extraction of SRM 2585 and extraction with conventional methods). Phase 2 analysis (wider scope of contaminants) was done at NILU. PAHs, substituted PAHs, PCBs, OCPs, NFRs, PBDEs, PHTHs, CPs, and dechloranes were analysed by GC–MS [30, 33, 34, 38–40]. CUPs, PFAS, OPFRs, PHTHs, bisphenols, HBCDs, BADGE, and parabens were analysed by LC–MS [31, 32, 41–43]. Full instrumental methods for all individual compound groups are given in the SI Text S1 and S2.

### QC/QA

Three filter (QFF) blanks and three solvent blanks per extraction method (hex:acet, MeOH, SUPRAS) were prepared and analysed. The filter and solvent blanks underwent identical treatment as the dust samples. Examination of the blank samples revealed that the predominant source of contamination originated from the filter blanks. As a result, the filter blanks were utilized for establishing the method detection limits (MDLs). The MDLs were determined by calculating the average of the filter blank measurements plus three times the standard deviation of the blank measurements (Table S4). If blanks were below detection, the instrumental quantification limit (IQL) was used as the MDL. The concentrations of the target compounds in each sample were then compared to the average concentrations in the filter blanks and treated as follows: if the concentration of the targeted compound was > MDL, the average blank level was subtracted from the measured level, and if the concentration of the targeted compound was < MDL, the values were recorded as < MDL.

As a quality control measure, samples were prepared in duplicates during the first phase (testing seven SUPRAS). The samples were prepared in triplicates in the subsequent second and third phases.

The recoveries were determined to assess the combined matrix and extraction effects. In phases 1 and 2, the recoveries were calculated as absolute recoveries of surrogate standards through either (depending on analytical method): (a) comparison of the peak areas in the sample compared with a control sample consisting of the surrogate standards in solvent, processed in the same instrumental run (for LC–MS analyses performed at RECETOX); or (b) by calculating the concentration of the surrogate in the sample based on a response factor (RF) determined in calibration standards and peak area of internal standard, and comparing it to the nominal concentration that was initially added (for all GC–MS analyses performed at RECETOX and all analyses performed at NILU). In phase 3, where concentrations of native compounds in the dust samples were determined, the recoveries were calculated relative to the surrogate, ensuring that the matrix and extraction effects were accounted for in the final quantification of the target compounds. We assessed the matrix effect by comparing surrogate recovery in blank samples to that in dust spiked samples. Consistent surrogate recovery between blank and spiked dust samples, with minimal deviation, indicated minimal matrix effects. Lower surrogate recovery in spiked samples suggested matrix suppression, whereas higher recovery indicated matrix enhancement.

To ensure comparability between the conventional extraction methods and SUPRAS, NIST SRM 2585 was extracted with conventional extraction methods (hex:acet for non-polar contaminants and MeOH for polar contaminants) and SUPRAS. The results were compared with the values in the NIST SRM 2585 Certificate of Analysis [44] and literature [45–52]. To quantify the difference between our measured values and the NIST-certified or literature values, we used adapted z-scores. Z-scores were calculated, using the following formula:

1 $$\text{z}-\text{score}=\frac{x - y}{0.25*y}$$

where *x* is the observed value and *y* is the certified/literature value. The z-scores therefore indicate the deviation between our observed values and the certified or literature values, considering an assumed 25% variation around those certified/literature values.

## Results and discussion

### Phase 1

In the initial phase of our experiment, we evaluated seven SUPRAS, formed from either hexanol or decanol as the key amphiphile components (Table 1) to simultaneously extract a diverse array of environmental contaminants.

SUPRAS 6 and 7 were synthesized using decanol, as different chain alcohols provide varying hydrophilic/lipophilic balance (HLB) and influence the SUPRAS composition and nanostructures, thus tuning the SUPRAS for different extraction properties. Both short- and long-chain alcohols can achieve good extraction efficiencies for polar and nonpolar compounds. Additionally, the ratio of alcohol to THF to water during SUPRAS formation plays a crucial role. Therefore, it is the complex equilibrium between these tuning factors that affects the extraction efficiency for compounds with different polarities [11, 16]. The formation of SUPRAS was successfully achieved, showing the inherent self-assembly properties of decanol-based amphiphiles. However, a significant challenge arose during the subsequent extraction process, when attempting to evaporate the decanol-based SUPRAS extracts using the N_2_ evaporation technique. Despite prolonged drying over several days, the evaporation of decanol-based SUPRAS extracts remained elusive under N_2_ flow. A vacuum evaporation system, such as a rotary evaporator, may offer a more effective approach; however, these decanol-based SUPRAS were not further tested in our method development.

SUPRAS 5, comprising Milli-Q, THF, and hexanol in a 30:60:10 ratio, respectively (Table 1), did not form the SUPRAS liquid phase. This is because at high concentrations of THF, the concentration of the coacervating agent (water) is too low to facilitate self-assembly and coacervation. Under these conditions, the concentration of water falls below the critical aggregation concentration (CAC) required for the system to form SUPRAS. As a result, the system does not form SUPRAS but rather a mixture of hexanol monomers or micelles diluted in THF:water [8, 11, 17]. Nevertheless, we proceeded to employ SUPRAS 5 for the extraction process. However, the average recovery (%) based on the recovery of surrogate standards representative for each group of contaminants was the lowest for many of the contaminant groups analysed (PAHs, O-PAHs, musks, PHTHs, and CUPs; Table 2, Table S5). We did not consider SUPRAS 5 further as the non-formation of SUPRAS was a large uncertainty and drawback.

**Table 2:**
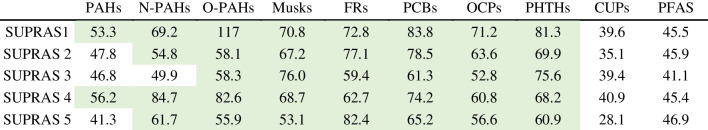
Average recovery (%) for surrogate standards of different groups of chemicals during phase 1. Average recoveries above 50% are marked in green

**Fig. 2:**
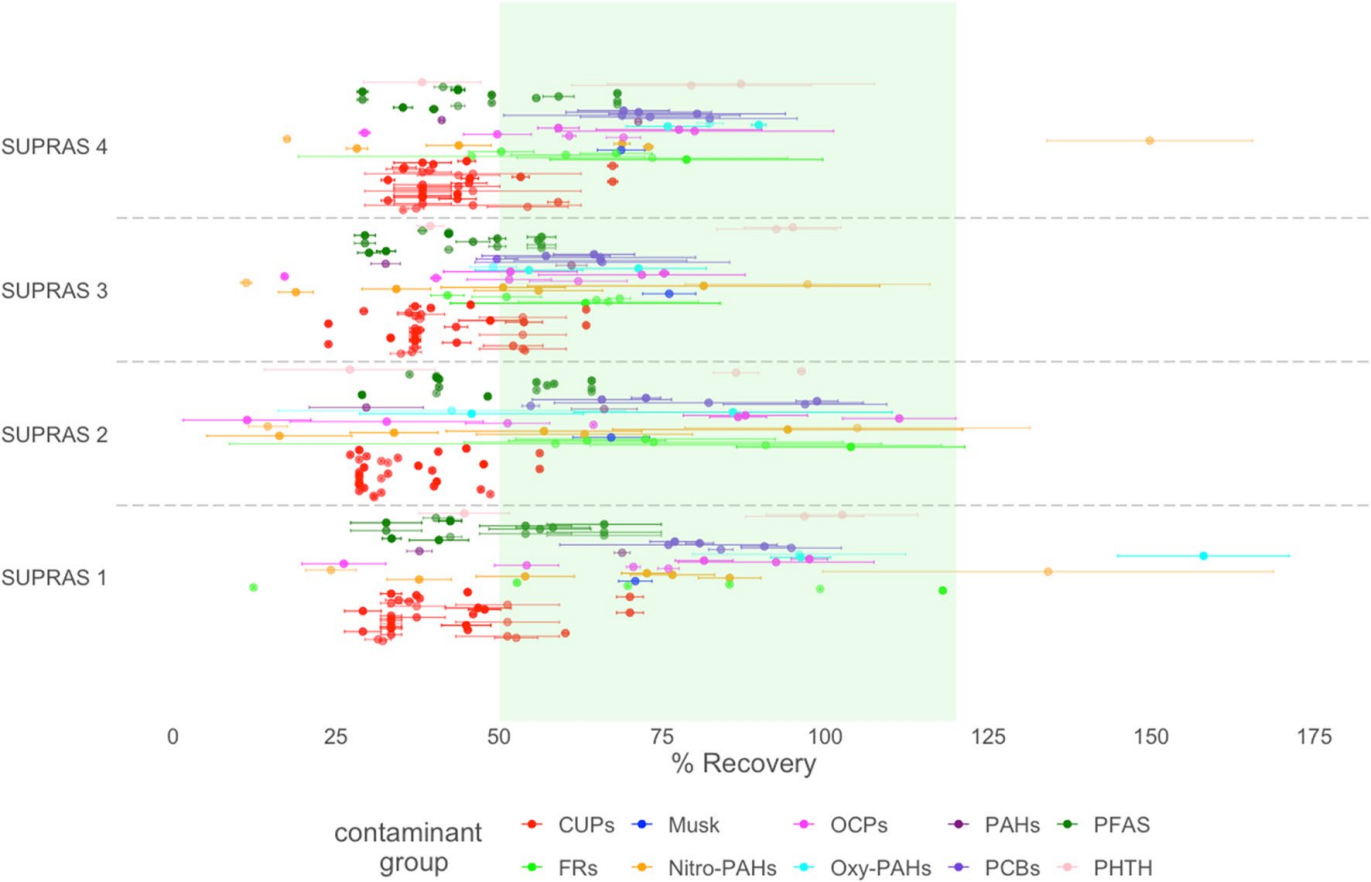
Extraction efficiency of SUPRAS 1, 2, 3, and 4 in phase 1 tests. The green shaded area indicates acceptable average recoveries of 50 to 120% for each contaminant group

The formation of the desired SUPRAS liquid phase in SUPRAS 1, 2, 3, and 4 was achieved and was therefore used for extraction. Among these, SUPRAS 2 displayed bigger variability between replicates and the broadest range of recoveries across different groups of contaminants. SUPRAS 1, 3, and 4 had the most consistent recoveries between replicates and among different contaminant groups (Fig. 2, Table S5). The best extraction performance was achieved using SUPRAS 1 and 4, where the average recoveries were above 50% across most of the contaminant groups, except for CUPs and PFAS, where recoveries failed to surpass 50% with any of the SUPRAS (Table 2). SUPRAS 1 and 4 yielded average recoveries of 53.3% and 56.2% for PAHs, 69.2% and 84.7% for nitro-PAHs, 117% and 82.6% for oxy-PAHs, 70.8% and 68.7% for musks, 72.8% and 62.7% for FRs, 83.8% and 74.2% for PCBs, 71.2% and 60.8% for OCPs, 81.3% and 68.2% for PHTHs, 39.6% and 40.9% for CUPs, and finally 45.5% and 45.4% for PFAS, respectively. Given that the bulk solution used to form SUPRAS for extraction of organic contaminants typically contains around 70% water [8, 10, 15], we selected SUPRAS 1 for further testing. We also estimated matrix effects from phase 1 analysis for SUPRAS 1 (Table S6).

### Phase 2

In the second phase, we broadened our assessment to include bisphenols, parabens, BADGE/BFDGE, HBCDs, OPFRs, dechloranes, and CPs in SUPRAS 1.

**Fig. 3:**
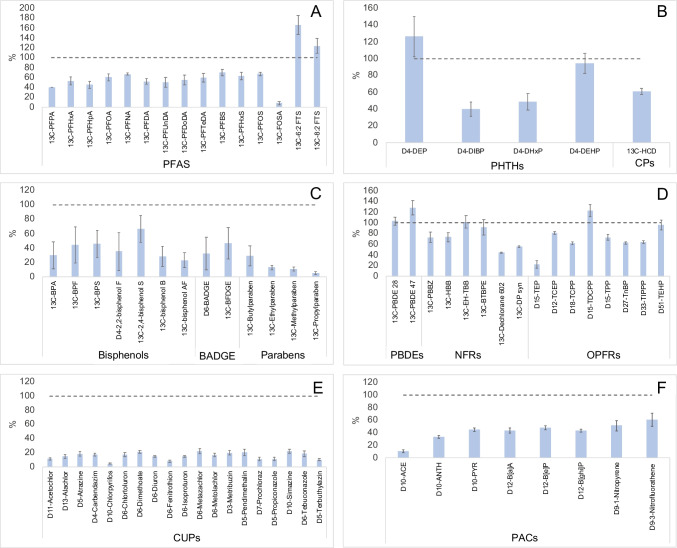
Extraction efficiency of surrogate standards with SUPRAS 1 for **A** PFAS; **B** PHTHs and CPs; **C** bisphenols, BADGE, and parabens; **D** PBDEs, NFRs, and OPFRs; **E** CUPs; and **F** PAHs and substituted PAHs. The bars represent average of three replicates, the error bars show standard deviation, and the dashed lines indicate a recovery of 100%

Among the bisphenols tested, we assessed the commonly used BPA, BPS, and BPF, with average recoveries of the surrogate standards of 29.8%, 45.9%, and 43.8%, respectively (Fig. 3, Table S7). Other bisphenols, such as bisphenol B, 2,4-bisphenol S, 2,2-bisphenol F, and bisphenol AF, exhibited average surrogate standard recoveries of 28.2%, 66.9%, 35.1%, and 23.0%, respectively. Recoveries of 31.9% and 46.9% were also achieved for BADGE and BFDGE, respectively. Four parabens—butyl, methyl, ethyl, and propyl—showed notably low recoveries, indicating less efficient extraction using SUPRAS 1 (Fig. 3, Table S7). Attempts to analyse HBCDs did not yield any data, marking the sole contaminant group where the SUPRAS method appeared ineffective.

Other flame retardants, including PBDEs, NFRs, and OPFRs, demonstrated good recoveries, ranging from 72.8 to 128% for PBDEs, from 44.2 to 102% for NFRs, and from 22.0 to 123% for OPFRs. Triethyl phosphate had the lowest recovery (22.0%) of the group, attributed to evaporation during extraction due to its higher volatility. A similar trend was observed for PAHs. Volatile PAHs like naphthalene evaporated entirely, while less volatile ones with higher molecular weights showed consistently better recoveries, averaging between 33.1 and 60.6% (Fig. 3, Table S7).

Four PHTHs—DEP, DiBP, DHxP, and DEHP—displayed recoveries of 126%, 40.0%, 48.5%, and 94.1%, respectively. Additionally, the chlorinated paraffins exhibited an average recovery of 61.0% (Fig. 3, Table S7). The results indicate that the extraction of these contaminants with SUPRAS is efficient.

For fifteen PFAS, average recoveries for fourteen ranged from 39.7 to 166%. However, FOSA displayed a very low recovery (7.86%), attributed to its volatile nature and evaporation during extraction, impacting detection and recovery (Fig. 3, Table S7).

Finally, CUPs had consistently low recoveries, ranging from 4.62 to 22.1% (Fig. 3, Table S7). The pesticides that have > 50% detection frequencies in European outdoor air [53] and were included in our study—atrazine, chlorpyrifos, metazachlor, metolachlor, tebuconazole, and terbuthylazine—all demonstrated very low recoveries, varying from 4.62% for chlorpyrifos to 21.7% for metazachlor (Fig. 3, Table S7). Previously, Peyrovi and Hadjmohammadi [54] found that the recoveries of selected CUPs, such as chlorpyrifos, were dependent on the chain length of the alkanol used (undecanol) and pH, with the most optimal being below or equal to pKa of their targeted pesticides. This may also explain the low recoveries in our study.

The SUPRAS-based extraction procedure developed in this study was found to be suitable for several groups of contaminants, notably flame retardants (PBDEs, NFRs, and OPFRs), certain bisphenols, PHTHs, PFAS, and chlorinated paraffins. However, it showed limitations with the extraction of HBCDs, and volatile compounds like triethyl phosphate, some PAHs, parabens, and FOSA, as well as very polar compounds, for example, selected CUPs. The low recoveries of volatile compounds are a limitation in the applicability; however, these compounds are typically of lesser importance in settled dust samples, which are dominated by less volatile compounds. Other sample handling strategies that could reduce losses during volume reduction steps may further expand the applicability of the proposed method to more volatile compounds as well. Low recoveries of very polar compounds such as CUPs could be improved by adding a salting out agent (e.g., using water with Na_2_SO_4_ instead of only water) to ensure the compounds are not extracted in the equilibrium solution. Alternatively, acidifying the water before SUPRAS formation below the pKa could prevent losses of acidic compounds. Furthermore, testing other SUPRAS, which have proven efficient in the simultaneous extraction of polar and nonpolar compounds, such as diol [3]- or acid [55]-based SUPRAS should be considered. Finally, increasing the ratio of SUPRAS to dust could enhance the recoveries.

### Phase 3

**Fig. 4:**
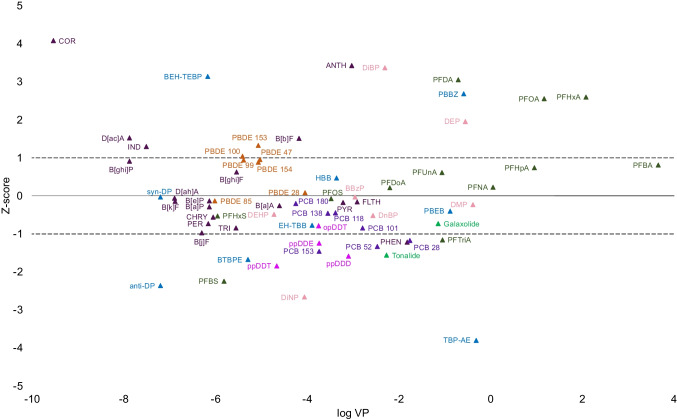
Concentrations of selected contaminants extracted with SUPRAS compared to the NIST certified/literature concentrations in SRM 2585. The z-scores show deviation between the observed values and the certified or literature values, considering a 25% variation around those certified/literature values. The *x*-axis shows the vapour pressure (Pa) of the contaminants on a logarithmic scale

In the final step, we compared results from SRM 2585 extracted with SUPRAS with the NIST SRM 2585 certified values where applicable, or with literature values when NIST certified values were not available (Fig. 4). Additionally, we also extracted SRM 2585 with the conventional methods, using hex:acet extraction for the non-polar contaminants and MeOH extraction for the polar contaminants. The results for comparison of NIST SRM 2585 certified values with those obtained by SUPRAS extraction and “conventional” hex:acet and MeOH extractions are presented in Table S8 and Figure S1.

SUPRAS reproduced PAH certified values extremely well for 17 of 20 PAHs (PHEN, FLTH, PYR, B[a]A, CHRY, B[b]F, B[k]F, B[a]P, IND, D[ah]A, B[ghi]P, B[ghi]F, TRI, B[j]F, B[e]P, PER, D[a,c]A), in many cases better than the commonly used hex:acet extraction (Table S8). The most volatile PAH, naphthalene, was absent, due to evaporation during extraction. Anthracene and coronene concentrations were two times higher in SUPRAS compared to the NIST-certified values, however comparable to the hex:acet extraction values. The concentrations of synthetic musks, galaxolide (HHCB), and tonalide (AHTN) extracted with SUPRAS were 1200 ng/g and 1040 ng/g, respectively. These values were within the range of NIST-certified values, with concentrations of 1470 ng/g for galaxolide and 1700 ng/g for tonalide (Fig. 4).

For the PCBs and OCPs, the findings indicated overall consistency between SUPRAS, hex:acet, and NIST-certified values for all PCBs and most OCPs. Concentrations of p,p′-DDT were half of the NIST-certified value (111 ng/g) in SUPRAS (59.9 ng/g) but were also substantially underreported in the hex:acet extraction (35.3 ng/g). SUPRAS extraction was not effective for PeCB, deviating by 680% deviation from the NIST-certified value, attributed to a very low recovery of PeCB surrogate, due to the volatility of the compound, resulting in misleading concentration calculation.

Out of the 12 PFAS analysed, eight PFAS (PBFA, PFHpA, PFNA, PFDoA, PFHxS, PFTriA, PFOS, and PFUnA) were within the 25% accepted variation of the literature values. PFHxA (429 ng/g), PFDA (67.2 ng/g), and PFOA (928 ng/g) were all approximately double the concentration in SUPRAS, although maintaining the same order of magnitude with the NIST certified/literature values (260 ng/g PFHxA, 38.1 ng/g PFDA, and 567 ng/g PFOA). In contrast, PFBS concentration was lower in SUPRAS (18.0 ng/g) compared to the literature value (40.8 ng/g) but again, remained within the same order of magnitude (Fig. 4, Table S8). The recoveries of PFAS surrogate standards were often below 50%, however, very consistent, which allowed for accurate quantification of PFAS in SRM 2585.

Most PBDEs (congeners 28, 47, 85, 99, 100, 153, and 154) largely aligned with previous data, although BDE-183 was two times higher in SUPRAS (106 ng/g) compared to the NIST-certified value (43.0 ng/g). Further clarification is necessary for BDE-209, which was not detected in SUPRAS-extracted SRM 2585 while the NIST SRM 2585 certified value is 2510 ng/g. With the hex:acet extraction, we observed a concentration of PBDE 209 of 2940 ng/g, which suggests that hex:acet extraction is a more optimal method (Table S8). We suspect that the large molecular size and low solubility of PBDE 209 could contribute to inefficient extraction with SUPRAS. The highly brominated PBDE 209 structure might hinder its interaction with the surfactants involved in the SUPRAS extraction, as was noted for HBCD.

Five PHTHs (DMP, DEP, DnBP, BBzP, and DEHP) demonstrated good consistency with literature values (Fig. 4). DiBP was two times higher in SUPRAS (11.9 ng/g) compared to the literature value (6.5 ng/g), although within the same order of magnitude. DiNP, however, was three times lower in SUPRAS, with a concentration of 66.6 ng/g, compared to the concentration of 199 ng/g in the literature (Table S8). However, we note that SRM 2585 is not certified for PHTHs; therefore, some greater uncertainty is expected.

Four NFRs, HBB, PBEB, EH-TBB, and syn-DP, extracted with SUPRAS developed in this study exhibited concentrations within the 25% accepted variation of the literature values [45, 51]. BEH-TEBP and PBBZ were two times higher, while anti-DP was two times lower in SUPRAS compared with the literature values, however maintaining the same order of magnitude. Only TBP-AE showed a significant discrepancy between SUPRAS (0.308 ng/g) and the literature value (6.0 ng/g) (Fig. 4, Table S8). As with PHTHs, this may be, in part, due to a lack of certified values.

To our knowledge, there is no SRM 2585 data available for substituted PAHs and CUPs in the NIST certificate or existing literature. Consequently, we only conducted a comparative assessment for substituted PAHs and CUPs between the SUPRAS extracts and hex:acet (substituted PAHs) and MeOH (CUPs) extracts.

Of the 35 CUPs analysed, only 11 (azinphos-methyl, carbaryl, diazinon, dimetachlor, chlorpyrifos, chlorsulfuron, isoproturon, pendimethalin, prochloraz, propiconazole, and tebuconazole) were detected in MeOH extracts, while only five (carbaryl, diazinon, chlorpyrifos, pendimethalin, and tebuconazole) were detected in SUPRAS extracts. The concentration of carbaryl was 1760 ng/g and 413 ng/g, diazinon was 282 ng/g and 226 ng/g, chlorpyrifos was 538 ng/g and 504 ng/g, pendimethalin was 21.7 ng/g and 45.8 ng/g, and tebuconazole was 0.584 ng/g and 2.17 ng/g for SUPRAS and MeOH, respectively. Only two compounds (diazinon and chlorpyrifos) were in agreement. Due to large variability between the CUP data and generally low recoveries (< 30%) of the surrogate standards, SUPRAS-based extraction using the current method is not recommended for CUPs.

Eighteen N-PAHs and nine O-PAHs were analysed, of which three N-PAHs and eight O-PAHs were detected in SUPRAS extracts and five N-PAHs and five O-PAHs were detected in hex:acet extracts. N-PAHs varied greatly between SUPRAS and hex:acet extracts, with only 7-nitrobenz[a]anthracene having comparable concentrations of 38.5 ng/g and 30.0 ng/g for SUPRAS and hex:acet, respectively. All O-PAHs except 1-naphthaldehyde were detected in SUPRAS extracts; however, they all had twofold higher concentrations compared to the hex:acet extracts. The concentration of 1,4-naphthoquinone in the SUPRAS extract was 122 µg/g, which seems highly implausible and can be attributed to a very low recovery (13%) of the surrogate standard.

### Limitations

The evaluation of SUPRAS for a wide range of compound groups, including FRs, PHTHs, PFAS, CPs, bisphenols, and others, has shown promising results. However, it is crucial to acknowledge and address specific limitations that have surfaced during this assessment.

While the SUPRAS selected in this study proved effective for many compound classes, challenges arise when dealing with volatile compounds, such as naphthalene, TEP, TBP-AE, FOSA, and PeCB. For instance, deuterated naphthalene and 13C-PeCB exhibited peak heights less than 10 times the noise level, leading to inaccuracies in the quantification of native naphthalene and PeCB. This observation confirms that the most volatile compounds are absent due to the evaporation step. In terms of additional method optimization, we recommend avoiding full drying of the extracts, but instead employing a solvent exchange method or to add a small volume of keeper, such as nonane, during the evaporation step. The evaporation of SUPRAS before LC–MS analysis may be unnecessary, as the extract is generally compatible with this technique. However, there are challenges related to implementing novel methods in routine instrumental analysis and concerns regarding the impacts of SUPRAS on instrumentation. While evaporation to dryness is typically unnecessary, if required for consistency with existing instrument practices, calibrants can be added to maintain accuracy and consistency. Calibrants can account for potential retention time shifts and variations in ionization, which can affect MS signals. When considering the loss of compounds due to evaporation, and if they are compatible with LC–MS, this approach should be noted as a potential solution. We employed sonication instead of the typically used vortex stirring during the extraction process. This alteration may have affected the extraction efficiency of the contaminants into the SUPRAS, potentially contributing to the lower recoveries observed. Additionally, the method faces difficulties with large, non-polar brominated compounds like HBCD and PBDE 209, as well as very polar compounds like CUPs. These findings highlight the need for further method optimization, or even a selection of different SUPRAS when these specific compounds/compound classes are of particular interest. The area of SUPRAS is developing rapidly, and there are multiple different SUPRAS, which have been shown to be efficient for the extraction of organic contaminants. These include for example cubosomic [56], vesicular [57], or magnetic [58] SUPRAS and should be tested in future studies.

When aiming for a wide-scope target screening method, some compromises need to be made as compared to typical targeted methods focusing on a single group of compounds. It is not possible to clean up the samples in a way that they meet the demands of the single specific targeted methods, because this will affect the detection of other groups of substances. The absence of a cleanup step in the extraction process can become an issue for the lifespan of GC components. This can lead to retention time shifts and suboptimal chromatography after a relatively small number of extracts. In addition, the lack of cleanup steps may contribute to challenges with matrix-related effects, requiring extra attention and caution during GC and LC–MS analyses. The matrix effects in this study showed high variability, with substantial matrix suppression for nitro-PAHs and oxy-PAHs and some NFRs, PCBs, and OCPs indicating impacts on analyte detection. Conversely, some compounds, such as many CUPs and PFAS, exhibited matrix enhancement (Table S6). This highlights the importance of considering matrix effects in analytical methods to ensure accurate quantification and reliable results and adjustments or calibrations might be necessary to account for these effects. For instance, due to the lack of a cleanup step in SUPRAS extracts in the quantification of DDT, the GC inlet and column became rapidly contaminated, resulting in a degradation of the 13C-p,p′-DDT, hindering accurate DDT quantification. While some matrix issues can be addressed through extract dilution or split injection, this approach may lead to higher limits of quantification and potential data loss due to lower concentrations of compounds of interest. Incorporation of cleanup steps or GC liner selection could enhance the instrument’s performance and mitigate these challenges.

## Conclusion

SUPRAS is a valuable extraction method suitable for the extraction of a wide range of environmental contaminants for both wide-scope target and suspect and non-target screening. It demonstrates robust performance for polar and nonpolar compounds, as seen in successful recoveries of surrogate standards and comparisons with NIST SRM 2585. The method proves valuable for analysing compounds such as FRs (OPFRs, NFRs, and PBDEs), PCBs, OCPs, PFAS, PHTHs, CPs, bisphenols, less volatile PAHs, and musks.

It is important to note that our data not only provide qualitative insights but also offer quantitative measurements, reinforcing the method’s reliability and applicability. The acknowledged limitations, particularly regarding volatile compounds and the absence of cleanup steps, emphasize the necessity for further method development and optimization. Nevertheless, SUPRAS persists as a promising, efficient, cost-effective, and environmentally friendly extraction tool with the potential for extensive applications.

### Supplementary Information

Below is the link to the electronic supplementary material.

Supplementary file1 (PDF 1243 KB)

## References

[1] Daryanavard SM, Zolfaghari H, Abdel-Rehim A, Abdel-Rehim M. Recent applications of microextraction sample preparation techniques in biological samples analysis. Biomed Chromatogr. 2021;35(7):e5105. 33660303 10.1002/bmc.5105

[2] Rubio S. Twenty years of supramolecular solvents in sample preparation for chromatography: achievements and challenges ahead. Anal Bioanal Chem. 2020;412(24):6037–58. 32206847 10.1007/s00216-020-02559-y

[3] González-Rubio S, Caballero-Casero N, Ballesteros-Gómez A, Cuervo D, Muñoz G, Rubio S. Supramolecular solvents for making comprehensive liquid-liquid microextraction in multiclass screening methods for drugs of abuse in urine based on liquid chromatography-high resolution mass spectrometry. J Chromatogr A. 2023;1701:464061. 37187096 10.1016/j.chroma.2023.464061

[4] Gutiérrez-Martín D, Restrepo-Montes E, Golovko O, López-Serna R, Aalizadeh R, Thomaidis NS, et al. Comprehensive profiling and semi-quantification of exogenous chemicals in human urine using HRMS-based strategies. Anal Bioanal Chem. 2023;415(29–30):7297–313. 37946034 10.1007/s00216-023-04998-9 PMC10684428

[5] Lambropoulou DA, Albanis TA. Liquid-phase micro-extraction techniques in pesticide residue analysis. J Biochem Biophys Methods. 2007;70(2):195–228. 17161462 10.1016/j.jbbm.2006.10.004

[6] Ochiai N, Sasamoto K, Kanda H, Pfannkoch E. Sequential stir bar sorptive extraction for uniform enrichment of trace amounts of organic pollutants in water samples. J Chromatogr A. 2008;1200(1):72–9. 18541254 10.1016/j.chroma.2008.05.069

[7] Buldini PL, Ricci L, Sharma JL. Recent applications of sample preparation techniques in food analysis. J Chromatogr A. 2002;975(1):47–70. 12458748 10.1016/S0021-9673(02)01335-3

[8] Ballesteros-Gómez A, Sicilia MD, Rubio S. Supramolecular solvents in the extraction of organic compounds A review. Anal Chim Acta. 2010;677(2):108–30. 20837178 10.1016/j.aca.2010.07.027

[9] Llompart M, Celeiro M, Dagnac T. Microwave-assisted extraction of pharmaceuticals, personal care products and industrial contaminants in the environment. TrAC Trends Anal Chem. 2019;116:136–50. 10.1016/j.trac.2019.04.029

[10] Dueñas-Mas MJ, Ballesteros-Gómez A, Rubio S. Supramolecular solvent-based microextraction probe for fast detection of bisphenols by ambient mass spectrometry. Chemosphere. 2022;294:133719. 35077738 10.1016/j.chemosphere.2022.133719

[11] Ballesteros-Gómez A, Lunar L, Sicilia MD, Rubio S. Hyphenating supramolecular solvents and liquid chromatography: tips for efficient extraction and reliable determination of organics. Chromatographia. 2019;82(1):111–24. 10.1007/s10337-018-3614-1

[12] González-Rubio S, Ballesteros-Gómez A, García-Gómez D, Rubio S. Double-headed amphiphile-based sponge droplets: synthesis, characterization and potential for the extraction of compounds over a wide polarity range. Talanta. 2022;239:123108. 34863061 10.1016/j.talanta.2021.123108

[13] Keddar MN, Ballesteros-Gómez A, Amiali M, Siles JA, Zerrouki D, Martín MA, et al. Efficient extraction of hydrophilic and lipophilic antioxidants from microalgae with supramolecular solvents. Sep Purif Technol. 2020;251:117327. 10.1016/j.seppur.2020.117327

[14] Sánchez-Vallejo C, Ballesteros-Gómez A, Rubio S. Tailoring composition and nanostructures in supramolecular solvents: impact on the extraction efficiency of polyphenols from vegetal biomass. Sep Purif Technol. 2022;292:120991. 10.1016/j.seppur.2022.120991

[15] Caballero-Casero N, Rubio S. Comprehensive supramolecular solvent-based sample treatment platform for evaluation of combined exposure to mixtures of bisphenols and derivatives by liquid chromatography-tandem mass spectrometry. Anal Chim Acta. 2021;1144:14–25. 33453791 10.1016/j.aca.2020.11.057

[16] Ballesteros-Gómez A, Rubio S. Environment-responsive alkanol-based supramolecular solvents: characterization and potential as restricted access property and mixed-mode extractants. Anal Chem. 2012;84(1):342–9. 22103906 10.1021/ac2026207

[17] Ballesteros-Gómez A, Ballesteros J, Rubio S. Comprehensive characterization of organic compounds in indoor dust after generic sample preparation with SUPRAS and analysis by LC-HRMS/MS. Sci Total Environ. 2024;912:169390. 38135084 10.1016/j.scitotenv.2023.169390

[18] Dueñas-Mas MJ, Ballesteros-Gómez A, Rubio S. Supramolecular solvent-based microextraction of aryl-phosphate flame retardants in indoor dust from houses and education buildings in Spain. Sci Total Environ. 2020;733:139291. 32450379 10.1016/j.scitotenv.2020.139291

[19] López-Jiménez FJ, Rosales-Marcano M, Rubio S. Restricted access property supramolecular solvents for combined microextraction of endocrine disruptors in sediment and sample cleanup prior to their quantification by liquid chromatography–tandem mass spectrometry. J Chromatogr A. 2013;1303:1–8. 23849783 10.1016/j.chroma.2013.06.043

[20] Li X, Huang A, Liao X, Chen J, Xiao Y. Restricted access supramolecular solvent based magnetic solvent bar liquid-phase microextraction for determination of non-steroidal anti-inflammatory drugs in human serum coupled with high performance liquid chromatography-tandem mass spectrometry. J Chromatogr A. 2020;1634:461700. 33229009 10.1016/j.chroma.2020.461700

[21] Salatti-Dorado JÁ, González-Rubio S, García-Gómez D, Lucena R, Cárdenas S, Rubio S. A high thermally stable oligomer-based supramolecular solvent for universal headspace gas chromatography: proof-of-principle determination of residual solvents in drugs. Anal Chim Acta. 2019;1046:132–9. 30482290 10.1016/j.aca.2018.09.023

[22] Takagai Y, Hinze WL. Cloud point extraction with surfactant derivatization as an enrichment step prior to gas chromatographic or gas chromatography−mass spectrometric analysis. Anal Chem. 2009;81(16):7113–22. 19621897 10.1021/ac9009963

[23] Wang Y, McCaffrey J, Norwood DL. Recent advances in headspace gas chromatography. J Liq Chromatogr Relat Technol. 2008;31(11–12):1823–51. 10.1080/10826070802129092

[24] Merino F, Rubio S, Pérez-Bendito D. Mixed aggregate-based acid-induced cloud-point extraction and ion-trap liquid chromatography–mass spectrometry for the determination of cationic surfactants in sewage sludge. J Chromatogr A. 2003;998(1–2):143–54. 12862380 10.1016/S0021-9673(03)00565-X

[25] Seebunrueng K, Dejchaiwatana C, Santaladchaiyakit Y, Srijaranai S. Development of supramolecular solvent based microextraction prior to high performance liquid chromatography for simultaneous determination of phenols in environmental water. RSC Adv. 2017;7(79):50143–9. 10.1039/C7RA07780G

[26] Martinefski M, Feizi N, Lunar ML, Rubio S. Supramolecular solvent-based high-throughput sample treatment platform for the biomonitoring of PAH metabolites in urine by liquid chromatography-tandem mass spectrometry. Chemosphere. 2019;237:124525. 31549648 10.1016/j.chemosphere.2019.124525

[27] Oliveira FMD, Scheel GL, Augusti R, Tarley CRT, Nascentes CC. Supramolecular microextraction combined with paper spray ionization mass spectrometry for sensitive determination of tricyclic antidepressants in urine. Anal Chim Acta. 2020;1106:52–60. 32145855 10.1016/j.aca.2020.01.061

[28] Melymuk L, Demirtepe H, Jílková SR. Indoor dust and associated chemical exposures. Curr Opin Environ Sci Health. 2020;15:1–6. 10.1016/j.coesh.2020.01.005

[29] Lucattini L, Poma G, Covaci A, De Boer J, Lamoree MH, Leonards PEG. A review of semi-volatile organic compounds (SVOCs) in the indoor environment: occurrence in consumer products, indoor air and dust. Chemosphere. 2018;201:466–82. 29529574 10.1016/j.chemosphere.2018.02.161

[30] Demirtepe H, Melymuk L, Diamond ML, Bajard L, Vojta Š, Prokeš R, et al. Linking past uses of legacy SVOCs with today’s indoor levels and human exposure. Environ Int. 2019;127:653–63. 30991221 10.1016/j.envint.2019.04.001

[31] Řiháčková K, Pindur A, Komprdová K, Pálešová N, Kohoutek J, Šenk P, et al. The exposure of Czech firefighters to perfluoroalkyl substances and polycyclic aromatic hydrocarbons: CELSPAC – FIREexpo case-control human biomonitoring study. Sci Total Environ. 2023;881:163298. 37054786 10.1016/j.scitotenv.2023.163298 PMC10230324

[32] Degrendele C, Prokeš R, Šenk P, Jílková SR, Kohoutek J, Melymuk L, et al. Human exposure to pesticides in dust from two agricultural sites in South Africa. Toxics. 2022;10(10):629. 36287909 10.3390/toxics10100629 PMC9610731

[33] Melymuk L, Jílková SR, Kolář M, Svobodová P, Vrana B, Hilscherová K. Questioning the appropriateness of sieving for processing indoor settled dust samples. J Environ Expo Assess. 2022;1(3):15. 10.20517/jeea.2022.12

[34] Wietzoreck M, Kyprianou M, Musa Bandowe BA, Celik S, Crowley JN, Drewnick F, et al. Polycyclic aromatic hydrocarbons (PAHs) and their alkylated, nitrated and oxygenated derivatives in the atmosphere over the Mediterranean and Middle East seas. Atmospheric Chem Phys. 2022;22(13):8739–66. 10.5194/acp-22-8739-2022

[35] Vykoukalová M, Venier M, Vojta Š, Melymuk L, Bečanová J, Romanak K, et al. Organophosphate esters flame retardants in the indoor environment. Environ Int. 2017;106:97–104. 28624751 10.1016/j.envint.2017.05.020

[36] Venier M, Audy O, Vojta Š, Bečanová J, Romanak K, Melymuk L, et al. Brominated flame retardants in the indoor environment — comparative study of indoor contamination from three countries. Environ Int. 2016;94:150–60. 27248661 10.1016/j.envint.2016.04.029

[37] Jílková S, Melymuk L, Vojta Š, Vykoukalová M, Bohlin-Nizzetto P, Klánová J. Small-scale spatial variability of flame retardants in indoor dust and implications for dust sampling. Chemosphere. 2018;206:132–41. 29734094 10.1016/j.chemosphere.2018.04.146

[38] Nežiková B, Degrendele C, Bandowe BAM, Holubová Šmejkalová A, Kukučka P, Martiník J, et al. Three years of atmospheric concentrations of nitrated and oxygenated polycyclic aromatic hydrocarbons and oxygen heterocycles at a central European background site. Chemosphere. 2021;269:128738. 33121801 10.1016/j.chemosphere.2020.128738

[39] Sobotka J, Smedes F, Vrana B. Performance comparison of silicone and low-density polyethylene as passive samplers in a global monitoring network for aquatic organic contaminants. Environ Pollut. 2022;302:119050. 35218918 10.1016/j.envpol.2022.119050

[40] Rusina TP, Jílková SR, Melymuk L, Vrana B, Smedes F. Accessibility investigation of semi-volatile organic compounds in indoor dust estimated by multi-ratio equilibrium passive sampling. Environ Res. 2023;219:115105. 36549487 10.1016/j.envres.2022.115105

[41] Routti H, Harju M, Lühmann K, Aars J, Ask A, Goksøyr A, et al. Concentrations and endocrine disruptive potential of phthalates in marine mammals from the Norwegian Arctic. Environ Int. 2021;152:106458. 33677245 10.1016/j.envint.2021.106458

[42] Lippold A, Harju M, Aars J, Blévin P, Bytingsvik J, Gabrielsen GW, et al. Occurrence of emerging brominated flame retardants and organophosphate esters in marine wildlife from the Norwegian Arctic. Environ Pollut. 2022;315:120395. 36228858 10.1016/j.envpol.2022.120395

[43] Hanssen L, Dudarev AA, Huber S, Odland JØ, Nieboer E, Sandanger TM. Partition of perfluoroalkyl substances (PFASs) in whole blood and plasma, assessed in maternal and umbilical cord samples from inhabitants of Arctic Russia and Uzbekistan. Sci Total Environ. 2013;447:430–7. 23410865 10.1016/j.scitotenv.2013.01.029

[44] National Institute of Standards and Technology. Standard Reference Material 2585, Organic contaminants in house dust. SRM 2582. U.S. Department of Commerce, Gaithersburg MD; 2018.

[45] Van Den Eede N, Dirtu AC, Ali N, Neels H, Covaci A. Multi-residue method for the determination of brominated and organophosphate flame retardants in indoor dust. Talanta. 2012;89:292–300. 22284495 10.1016/j.talanta.2011.12.031

[46] Larsson K, Lindh CH, Jönsson BA, Giovanoulis G, Bibi M, Bottai M, et al. Phthalates, non-phthalate plasticizers and bisphenols in Swedish preschool dust in relation to children’s exposure. Environ Int. 2017;102:114–24. 28274486 10.1016/j.envint.2017.02.006

[47] Luongo G, Östman C. Organophosphate and phthalate esters in settled dust from apartment buildings in Stockholm. Indoor Air. 2016;26(3):414–25. 25929991 10.1111/ina.12217

[48] Mercier F, Gilles E, Saramito G, Glorennec P, Le Bot B. A multi-residue method for the simultaneous analysis in indoor dust of several classes of semi-volatile organic compounds by pressurized liquid extraction and gas chromatography/tandem mass spectrometry. J Chromatogr A. 2014;1336:101–11. 24598454 10.1016/j.chroma.2014.02.004

[49] Weiss JM, Gustafsson Å, Gerde P, Bergman Å, Lindh CH, Krais AM. Daily intake of phthalates, MEHP, and DINCH by ingestion and inhalation. Chemosphere. 2018;208:40–9. 29860143 10.1016/j.chemosphere.2018.05.094

[50] Bergh C, Luongo G, Wise S, Östman C. Organophosphate and phthalate esters in standard reference material 2585 organic contaminants in house dust. Anal Bioanal Chem. 2012;402(1):51–9. 22065343 10.1007/s00216-011-5440-2

[51] Fan X, Kubwabo C, Rasmussen PE, Wu F. Non-PBDE halogenated flame retardants in Canadian indoor house dust: sampling, analysis, and occurrence. Environ Sci Pollut Res. 2016;23(8):7998–8007. 10.1007/s11356-015-5956-7 26780041

[52] Reiner JL, Blaine AC, Higgins CP, Huset C, Jenkins TM, Kwadijk CJAF, et al. Polyfluorinated substances in abiotic standard reference materials. Anal Bioanal Chem. 2015;407(11):2975–83. 26005739 10.1007/s00216-013-7330-2

[53] Mayer L, Degrendele C, Šenk P, Kohoutek J, Přibylová P, Kukučka P, et al. Widespread pesticide distribution in the European atmosphere questions their degradability in air. Environ Sci Technol. 2024;58(7):3342–52. 38323876 10.1021/acs.est.3c08488 PMC10882970

[54] Peyrovi M, Hadjmohammadi M. Alkanol-based supramolecular solvent microextraction of organophosphorus pesticides and their determination using high-performance liquid chromatography. J Iran Chem Soc. 2017;14(5):995–1004. 10.1007/s13738-017-1049-5

[55] Luque N, Ballesteros-Gómez A, Van Leeuwen S, Rubio S. A simple and rapid extraction method for sensitive determination of perfluoroalkyl substances in blood serum suitable for exposure evaluation. J Chromatogr A. 2012;1235:84–91. 22420956 10.1016/j.chroma.2012.02.055

[56] González-Rubio S, Ballesteros-Gómez A, Muñoz G, Rubio S. Cubosomic supramolecular solvents: synthesis, characterization, and potential for high-throughput multiclass testing of banned substances in urine. Anal Chem. 2022;94(9):4103–11. 35191686 10.1021/acs.analchem.2c00082

[57] Moral A, Sicilia MD, Rubio S. Determination of benzimidazolic fungicides in fruits and vegetables by supramolecular solvent-based microextraction/liquid chromatography/fluorescence detection. Anal Chim Acta. 2009;650(2):207–13. 19720194 10.1016/j.aca.2009.07.056

[58] Zohrabi P, Shamsipur M, Hashemi M, Hashemi B. Liquid-phase microextraction of organophosphorus pesticides using supramolecular solvent as a carrier for ferrofluid. Talanta. 2016;160:340–6. 27591622 10.1016/j.talanta.2016.07.036

