# Supplementary material for: Development of a supramolecular solvent–based extraction method for application to quantitative analyses of a wide range of organic contaminants in indoor dust
Source: Anal Bioanal Chem. 2024 Jul 12;416(22):4973–85. doi: 10.1007/s00216-024-05433-3 (PMC11330406; doi:10.1007/s00216-024-05433-3)
Supplement: Supplementary file 1 — Supplementary file1 (PDF 1243 KB) [file 216_2024_5433_MOESM1_ESM.pdf]

Supporting information for

Development of a supramolecular solvent-based extraction method for application to quantitative analyses of a wide range of organic contaminants in indoor dust

Paula Marcinekova[1], Lisa Melymuk[1],* Pernilla Bohlin-Nizzetto[2], Erika Martinelli[2], Simona Rozárka Jílková[1], Jakub Martinik[1], Petr Šenk[1], Petr Kukučka[1], Ondřej Audy[1], Jiří Kohoutek[1], Mebrat Ghebremeskel[2], Alexander Håland[2], Anders Røsrud Borgen[2], Heidi Eikenes[2], Linda Hanssen[3], Mikael Harju[3], Zofia Cebula[4], Pawel Rostkowski[2,*]

(1) RECETOX, Masaryk University, Faculty of Science, Kotlářská 2, 61137 Brno, Czechia
(2) NILU, Instituttveien 18, 2007, Kjeller, Norway
(3) NILU, Fram Center, Hjalmar Johansens gate 14, 9007, Tromsø, Norway
(4) Institute of Biotechnology and Molecular Medicine, Kampinoska 25, 80-180, Gdańsk, Poland

* Corresponding authors:

Lisa Melymuk, RECETOX, Masaryk University, Faculty of Science, Kotlářská 2, 61137 Brno, Czechia, lisa.melymuk@recetox.muni.cz

Pawel Rostkowski, NILU, Instituttveien 18, 2007, Kjeller, Norway, pr@nilu.no

Contents

Text S1 Instrumental analysis at RECETOX laboratories S20

Text S2 Instrumental analysis at NILU laboratories S22

Table S1:Surrogate standards and internal standards used in analysis at NILU Laboratories. S2

Table S2: Surrogate standards and internal standards used in analysis at RECETOX Laboratories..... S4

Table S3: List of compounds included in the method development and relevant physical-chemical properties S6

Table S4: Instrument detection and quantification limits and blank concentrations for Phase 3 extractions. S25

Table S5: Percentage recoveries from Phase 1 analysis S28

Table S6: Matrix effect from Phase 1 analysis calculated for SUPRAS 1 .... S31

Table S7: Percentage recoveries for Phase 2 analysis S32

Table S8: Comparison of SRM 2585 certified/literature values (ng/g) with those obtained by SUPRAS extraction and "conventional" hex:ace and MeOH extractions S34

Figure S1: Comparison of SRM 2585 certified/literature values with those obtained by SUPRAS extraction and "conventional" extractions S36

Table S1:Surrogate standards (added prior to extraction) and internal standards (added prior to injection, marked in italic in the table) used in analysis at NILU Laboratories.

	Standard	Conc.	Unit	Spike volume (µl)	Supplier
Bisphenols and consumer products	13C12 4,4'-Bisphenol A	599.2	pg/µl	50	Cambridge Isotope Laboratories, Inc.
	13C12 Bisphenol B	631.0	pg/µl	50	Cambridge Isotope Laboratories, Inc.
	13C12 4,4'-Bisphenol F	582.6	pg/µl	50	Cambridge Isotope Laboratories, Inc.
	D4 2,2'-Bisphenol F	579.6	pg/µl	50	Toronto Research Chemicals, Inc.
	13C4 Bisphenol P	598.1	pg/µl	50	Toronto Research Chemicals, Inc.
	13C12 4,4'-Bisphenol S	606.8	pg/µl	50	Cambridge Isotope Laboratories, Inc.
	13C12 2,4'-Bisphenol S	591.9	pg/µl	50	Toronto Research Chemicals, Inc.
	13C12 Bisphenol Z	596.9	pg/µl	50	Toronto Research Chemicals, Inc.
	13C12 Bisphenol AF	604.9	pg/µl	50	Cambridge Isotope Laboratories, Inc.
	13C12 TBBPA	595.1	pg/µl	50	Cambridge Isotope Laboratories, Inc.
	13C6 p-n-Nonylphenol	603.6	pg/µl	50	Cambridge Isotope Laboratories, Inc.
	13C6 4-tert-Octylphenol	530.3	pg/µl	50	Sigma-Aldrich
	13C12 Triclosan	589.2	pg/µl	50	Cambridge Isotope Laboratories, Inc.
	D9 4-Tert-Butylphenol	*1224.7*	*pg/µl*	*50*	*Toronto Research Chemicals, Inc.*
	D8- 4,4'-Bisphenol A	*589*	*pg/µl*	*20*	*Toronto Research Chemicals, Inc.*
	D10-4,4'- Bisphenol F	*605*	*pg/µl*	*20*	*Toronto Research Chemicals, Inc.*
	D8-Bisphenol B	*593*	*pg/µl*	*20*	*Toronto Research Chemicals, Inc.*
	D16-Bisphenol P	*600*	*pg/µl*	*20*	*Toronto Research Chemicals, Inc.*
PBDEs	13C PBDE-28	127.8	pg/µl	50	Cambridge Isotope Laboratories, Inc.
	13C PBDE-47	128.5	pg/µl	50	Cambridge Isotope Laboratories, Inc.
	13C PBDE-99	131.7	pg/µl	50	Cambridge Isotope Laboratories, Inc.
	13C PBDE-153	128.4	pg/µl	50	Cambridge Isotope Laboratories, Inc.
	13C PBDE-183	130.9	pg/µl	50	Cambridge Isotope Laboratories, Inc.
	13C PBDE-197	129.0	pg/µl	50	Cambridge Isotope Laboratories, Inc.
	13C PBDE-206	126.2	pg/µl	50	Cambridge Isotope Laboratories, Inc.
	13C PBDE-209	634.7	pg/µl	50	Cambridge Isotope Laboratories, Inc.
NFRs	13C6 BTBPE	505.0	pg/µl	50	Wellington Laboratories, Canada
	13C6 HBB	514.2	pg/µl	50	Wellington Laboratories, Canada
	13C6 D17 EH-TBB	511.9	pg/µl	50	Wellington Laboratories, Canada
	13C14 DBDPE	493.6	pg/µl	50	Wellington Laboratories, Canada
	13C6 PBBZ	495.8	pg/µl	50	Wellington Laboratories, Canada
	13C6 D17 BEH-TEBP	505.8	pg/µl	50	Wellington Laboratories, Canada
	D6 p-TBX	*521.8*	*pg/µl*	*50*	*Toronto Research Chemicals, Inc.*
PCBs	13C PeCB	9.6	pg/µl	50	Cambridge Isotope Laboratories, Inc.
	13C HCB	9.4	pg/µl	50	Cambridge Isotope Laboratories, Inc.
	13C PCB- 28	23.8	pg/µl	50	Cambridge Isotope Laboratories, Inc.
	13C PCB- 52	24.0	pg/µl	50	Cambridge Isotope Laboratories, Inc.
	13C PCB- 101	23.7	pg/µl	50	Cambridge Isotope Laboratories, Inc.
	13C PCB- 105	24.0	pg/µl	50	Cambridge Isotope Laboratories, Inc.
	13C PCB- 114	23.8	pg/µl	50	Cambridge Isotope Laboratories, Inc.
	13C PCB- 118	23.7	pg/µl	50	Cambridge Isotope Laboratories, Inc.
	13C PCB- 123	24.3	pg/µl	50	Cambridge Isotope Laboratories, Inc.
	13C PCB- 138	23.8	pg/µl	50	Cambridge Isotope Laboratories, Inc.
	13C PCB- 153	23.9	pg/µl	50	Cambridge Isotope Laboratories, Inc.
	13C PCB- 156	23.7	pg/µl	50	Cambridge Isotope Laboratories, Inc.
	13C PCB- 157	23.7	pg/µl	50	Cambridge Isotope Laboratories, Inc.
	13C PCB- 167	23.8	pg/µl	50	Cambridge Isotope Laboratories, Inc.
	13C PCB- 180	23.9	pg/µl	50	Cambridge Isotope Laboratories, Inc.
	13C PCB- 189	23.8	pg/µl	50	Cambridge Isotope Laboratories, Inc.
	13C PCB- 209	23.7	pg/µl	50	Cambridge Isotope Laboratories, Inc.
OCPs	13C Tris-Nonachlor	73.9	pg/µl	50	Cambridge Isotope Laboratories, Inc.
	13C Cis-Nonachlor	51.3	pg/µl	50	Cambridge Isotope Laboratories, Inc.
	13C Tris-Chlordane	50.9	pg/µl	50	Cambridge Isotope Laboratories, Inc.
	13C Cis-Chlordane	74.6	pg/µl	50	Cambridge Isotope Laboratories, Inc.
	13C Oxychlordane	515.9	pg/µl	50	Cambridge Isotope Laboratories, Inc.
	13C Heptachlor epoxide	1003.3	pg/µl	50	Cambridge Isotope Laboratories, Inc.
	13C Heptachlor	1211.8	pg/µl	50	Cambridge Isotope Laboratories, Inc.
	13C Dieldrin	1001.9	pg/µl	50	Cambridge Isotope Laboratories, Inc.
	13C Mirex	260.9	pg/µl	50	Cambridge Isotope Laboratories, Inc.
	13C Endosulfan I	99.0	pg/µl	50	Cambridge Isotope Laboratories, Inc.
	13C Endosulfan II	99.4	pg/µl	50	Cambridge Isotope Laboratories, Inc.
	13C Endosulfan Sulfate	74.3	pg/µl	50	Cambridge Isotope Laboratories, Inc.
	D14 Trifluralin (di-n-propyl)	74.7	pg/µl	50	Cambridge Isotope Laboratories, Inc.
	13C Endrin	981.3	pg/µl	50	Cambridge Isotope Laboratories, Inc.
	13C Aldrin	1009.2	pg/µl	50	Cambridge Isotope Laboratories, Inc.
	13C Isodrin	1972.9	pg/µl	50	Cambridge Isotope Laboratories, Inc.
[illegible]	13C10 Hexachlorodecane	328.0	pg/µl	50	Cambridge Isotope Laboratories, Inc.

	13C12 Octachlorodecane	318.2	pg/µl	50	Cambridge Isotope Laboratories, Inc.
	13C10 Dechlorane Plus Syn	99.4	pg/µl	50	Cambridge Isotope Laboratories, Inc.
	13C10 Dechlorane 602	104.4	pg/µl	50	Cambridge Isotope Laboratories, Inc.
Parabens	13C6 Methyl-4-Hydroxybenzoate	1.2	ng/µl	50	Sigma-Aldrich
	13C6 Ethyl-4-Hydroxybenzoate	1.2	ng/µl	50	Sigma-Aldrich
	13C6 Propyl-4-Hydroxybenzoate	1.2	ng/µl	50	Sigma-Aldrich
	13C6 Butyl-4-Hydroxybenzoate	1.2	ng/µl	50	Sigma-Aldrich
	D21 BHT	*1.3*	*ng/µl*	*50*	*Cambridge Isotope Laboratories, Inc.*
	D21 2,4-Di-tert-butylphenol	*1.2*	*ng/µl*	*50*	*Toronto Research Chemicals, Inc.*
	D9 4-Tert-butylphenol	*1.2*	*ng/µl*	*50*	*Toronto Research Chemicals, Inc.*
	D7 4-(4-Isopropoxyphenylsulfonyl)phenol	*1.2*	*ng/µl*	*50*	*Toronto Research Chemicals, Inc.*
N-PAHs	D9 1-Nitropyrene	2.5	ng/µl	50	Chiron AS, Norway
	D9 3-Nitrofluorathene-	2.5	ng/µl	50	Chiron AS, Norway
PAHs	D10 2-Methylnaphthalane	109.4	ng/µl	20	Chiron AS, Norway
	D10 Acenaphthene	115.9	ng/µl	20	Chiron AS, Norway
	D10 Anthracene	119.3	ng/µl	20	Chiron AS, Norway
	D10 Pyrene	125.9	ng/µl	20	Chiron AS, Norway
	D12 Benz(a)anthracene	119.2	ng/µl	20	Chiron AS, Norway
	D12 Benzo€pyrene	114.1	ng/µl	20	Chiron AS, Norway
	D12 Benzo(g,h,i)perylene	114.5	ng/µl	20	Chiron AS, Norway
	D10- Biphenyl	*1.40*	*ng/µl*	*20*	*Chiron AS, Norway*
	D10-Fluoranthene	*1.45*	*ng/µl*	*20*	*Chiron AS, Norway*
	D12- Perylene	*1.53*	*ng/µl*	*20*	*Chiron AS, Norway*
HBCD	13C α-HBCD	253.6	pg/µl	50	Wellington Laboratories, Canada
	13C β-HBCD	258.2	pg/µl	50	Wellington Laboratories, Canada
	13C γ-HBCD	256.5	pg/µl	50	Wellington Laboratories, Canada
	D18 α-HBCD	*124*	*pg/µl*	*20*	*Wellington Laboratories, Canada*
	D18 β-HBCD	*124*	*pg/µl*	*20*	*Wellington Laboratories, Canada*
	D18 γ-HBCD	*123*	*pg/µl*	*20*	*Wellington Laboratories, Canada*
BADGE	D6 BADGE	4.7	ng/µl	50	Toronto Research Chemicals, Inc.
	13C12 BFDGE	5.0	ng/µl	50	Cambridge Isotope Laboratories, Inc.
	D10 BADGE	*1.29*	*ng/µl*	*20*	*Toronto Research Chemicals, Inc.*
PFAS	13C4 PFBA	0.5	ng/µl	50	Wellington Laboratories, Canada
	13C5 PFPeA	0.5	ng/µl	50	Wellington Laboratories, Canada
	13C5 PFHxA	0.5	ng/µl	50	Wellington Laboratories, Canada
	13C4 PFHpA	0.5	ng/µl	50	Wellington Laboratories, Canada
	13C4 PFOA	0.5	ng/µl	50	Wellington Laboratories, Canada
	13C5 PFNA	0.5	ng/µl	50	Wellington Laboratories, Canada
	13C6 PFDA	0.5	ng/µl	50	Wellington Laboratories, Canada
	13C7 PFUnDA	0.5	ng/µl	50	Wellington Laboratories, Canada
	13C2 PFDoDA	0.5	ng/µl	50	Wellington Laboratories, Canada
	13C2 PFTeDA	0.5	ng/µl	50	Wellington Laboratories, Canada
	13C2 PFHxDA	0.5	ng/µl	50	Wellington Laboratories, Canada
	13C3 PFBS	0.5	ng/µl	50	Wellington Laboratories, Canada
	13C3 PFHxS	0.5	ng/µl	50	Wellington Laboratories, Canada
	13C4 PFOS	0.5	ng/µl	50	Wellington Laboratories, Canada
	13C8 FOSA	0.5	ng/µl	50	Wellington Laboratories, Canada
	13C2 6:2 FTS	0.5	ng/µl	50	Wellington Laboratories, Canada
	13C2 8:2 FTS	0.5	ng/µl	50	Wellington Laboratories, Canada
	3,7-dimethyl PFOA	*0.1*	*ng/µl*	*20*	*Wellington Laboratories, Canada*
OPFRs	D27-TnBP	0.5	ng/µl	50	Cambridge Isotope Laboratories, Inc.
	D33-TIPPP	0.5	ng/µl	50	Cambridge Isotope Laboratories, Inc.
	D15-TEP	0.5	ng/µl	50	Cambridge Isotope Laboratories, Inc.
	D18-TCPP	0.5	ng/µl	50	Cambridge Isotope Laboratories, Inc.
	D12-TCEP	0.5	ng/µl	50	Cambridge Isotope Laboratories, Inc.
	D15-TDCPP	0.5	ng/µl	50	Cambridge Isotope Laboratories, Inc.
	D15-TPHP	0.5	ng/µl	50	Cambridge Isotope Laboratories, Inc.
	D51-TEHP	0.5	ng/µl	50	Cambridge Isotope Laboratories, Inc.
	d27-Tris(2,4-dimethylphenyl) phosphate	*0.5*	*ng/µl*	*20*	*Cambridge Isotope Laboratories, Inc.*
PTHTs	D4 Dimethyl phthalate	5.0	ng/µl	50	Accustandard, Inc.
	D4 Diethyl phthalate	5.0	ng/µl	50	Accustandard, Inc.
	D4 Di-n-pentyl phthalate	5.0	ng/µl	50	Accustandard, Inc.
	D4 Di-n-butyl phthalate	5.0	ng/µl	50	Accustandard, Inc.
	D4 Di-iso-butyl phthalate	5.0	ng/µl	50	Accustandard, Inc.
	D4 Dibenzyl phthalate	5.0	ng/µl	50	Accustandard, Inc.
	D4 Dicyclohexyl phthalate	5.0	ng/µl	50	Accustandard, Inc.
	D4 Di-n-hexyl phthalate	5.0	ng/µl	50	Accustandard, Inc.
	D4 Din-n-propyl phthalate	*5.0*	*ng/µl*	*20*	*Accustandard, Inc.*
	D4 Di-n-octyl phthalate	*5.0*	*ng/µl*	*20*	*Accustandard, Inc.*

IS for POPs	*1,2,3,4-tetrachloronaphthalene*	*21.3*	*pg/µl*	*20*	*UltraScientific/Agilent*

Table S2: Surrogate standards (added prior to extraction) and internal standards (added prior to injection, marked in italic in the table) used in analysis at RECETOX Laboratories.

	**Standards**	**Conc.**	**Unit**	**Spike volume (µl)**	**Supplier**
CUPs	Acetochlor-D11	1	µg/ml	50	Dr. Ehrenstorfer, Germany
	Alachlor-D13	1	µg/ml	50	Absolute Standards, US
	Atrazine-D5	1	µg/ml	50	Dr. Ehrenstorfer, Germany
	Carbendazim-D4	1	µg/ml	50	HPC Standards, Germany
	Chlorpyrifos-D10	1	µg/ml	50	Dr. Ehrenstorfer, Germany
	Chlortoluron-D6	1	µg/ml	50	Dr. Ehrenstorfer, Germany
	Dimethoate-D6	1	µg/ml	50	HPC Standards, Germany
	Diuron-D6	1	µg/ml	50	HPC Standards, Germany
	Fenitrothion-D6	1	µg/ml	50	Dr. Ehrenstorfer, Germany
	Isoproturon-D6	1	µg/ml	50	Dr. Ehrenstorfer, Germany
	Metamitron-D5	1	µg/ml	50	HPC Standards, Germany
	Metazachlor-D6	1	µg/ml	50	HPC Standards, Germany
	Metolachlor-D6	1	µg/ml	50	Dr. Ehrenstorfer, Germany
	Metribuzin-D3	1	µg/ml	50	HPC Standards, Germany
	Pendimethalin-D5	1	µg/ml	50	Dr. Ehrenstorfer, Germany
	Phosmet-D6	1	µg/ml	50	Dr. Ehrenstorfer, Germany
	Prochloraz-D7	1	µg/ml	50	Dr. Ehrenstorfer, Germany
	Propiconazole-D5	1	µg/ml	50	Dr. Ehrenstorfer, Germany
	Pyrazon-D5	1	µg/ml	50	Dr. Ehrenstorfer, Germany
	Simazine-D10	1	µg/ml	50	Dr. Ehrenstorfer, Germany
	Tebuconazole-D6	1	µg/ml	50	Dr. Ehrenstorfer, Germany
	Terbuthylazine-D5	1	µg/ml	50	Dr. Ehrenstorfer, Germany
PFAS	Perfluoro-n-[1,2,3,4-13C4]butanoic acid (MPFBA)	0.04	µg/ml	50	Wellington Laboratories, Canada
	Perfluoro-n-[1,2-13C2]hexanoic acid (MPFHxA)	0.04	µg/ml	50	Wellington Laboratories, Canada
	Perfluoro-n-[1,2,3,4-13C4]octanoic acid (MPFOA)	0.04	µg/ml	50	Wellington Laboratories, Canada
	Perfluoro-n-[1,2,3,4,5,-13C5]nonanoic acid (MPFNA)	0.04	µg/ml	50	Wellington Laboratories, Canada
	Perfluoro-n-[1,2-13C2]decanoic acid (MPFDA)	0.04	µg/ml	50	Wellington Laboratories, Canada
	Perfluoro-n-[1,2,-13C2]undecanoic acid (MPFUnDA)	0.04	µg/ml	50	Wellington Laboratories, Canada
	Perfluoro-n-[1,2-13C2]dodecanoic acid (MPFDoDA)	0.04	µg/ml	50	Wellington Laboratories, Canada
	Sodium perfluoro-1-[18O2]hexanesulfonate (MPFHxS)	0.04	µg/ml	50	Wellington Laboratories, Canada
	Sodium perfluoro-1-[1,2,3,4-13C4]-octanesulfonate (MPFOS)	0.04	µg/ml	50	Wellington Laboratories, Canada
	Sodium perfluoro-1-[1,2,3-13C3]butanesulfonate (MPFBS)	0.04	µg/ml	50	Wellington Laboratories, Canada
PTHTs	D4 BBzP	4	µg/ml	50	Cambridge Isotope Laboratories, US
	D4 DEP	4	µg/ml	50	Supelco / Sigma-Aldrich, US
	D4 DEHP	4	µg/ml	50	Supelco / Sigma-Aldrich, US
PAHs	D8 Naphthalene	6.66	µg/ml	50	Supelco / Sigma-Aldrich, US
	D10 Phenanthrene	6.66	µg/ml	50	Supelco / Sigma-Aldrich, US
	D12 Perylene	6.66	µg/ml	50	Supelco / Sigma-Aldrich, US
OCPs	13C6 PeCB	0.2	µg/ml	50	Cambridge Isotope Laboratories, US
	13C6 HCB	0.2	µg/ml	50	Cambridge Isotope Laboratories, US
	13C6 β-HCH	0.2	µg/ml	50	Wellington Laboratories, Canada
	13C6 γ-HCH	0.2	µg/ml	50	Cambridge Isotope Laboratories, US
	13C12 ppDDE	0.2	µg/ml	50	Cambridge Isotope Laboratories, US
	13C12 ppDDD	0.2	µg/ml	50	Cambridge Isotope Laboratories, US
	13C12 ppDDT	0.2	µg/ml	50	Cambridge Isotope Laboratories, US
PCBs	13C12 PCB 28	0.2	µg/ml	50	Cambridge Isotope Laboratories, US
	13C12 PCB 52	0.2	µg/ml	50	Cambridge Isotope Laboratories, US
	13C12 PCB 101	0.2	µg/ml	50	Cambridge Isotope Laboratories, US
	13C12 PCB 153	0.2	µg/ml	50	Cambridge Isotope Laboratories, US
	13C12 PCB 138	0.2	µg/ml	50	Cambridge Isotope Laboratories, US
	13C12 PCB 180	0.2	µg/ml	50	Cambridge Isotope Laboratories, US
	13C12 PCB 209	0.2	µg/ml	50	Cambridge Isotope Laboratories, US
Musks	D3 AHTN	4	µg/ml	50	Neochema, Germany

PBDEs	13C12 PBDE 28	0.02	µg/ml	50	Wellington Laboratories, Canada
	13C12 PBDE 47	0.02	µg/ml	50	Wellington Laboratories, Canada
	13C12 PBDE 99	0.02	µg/ml	50	Wellington Laboratories, Canada
	13C12 PBDE 100	0.02	µg/ml	50	Wellington Laboratories, Canada
	13C12 PBDE 153	0.02	µg/ml	50	Wellington Laboratories, Canada
	13C12 PBDE 154	0.02	µg/ml	50	Wellington Laboratories, Canada
	13C12 PBDE 183	0.02	µg/ml	50	Wellington Laboratories, Canada
	13C12 PBDE 209	0.1	µg/ml	50	Wellington Laboratories, Canada
NFRs	13C6 PBBZ	0.02	µg/ml	50	Wellington Laboratories, Canada
	13C6 HBB	0.02	µg/ml	50	Wellington Laboratories, Canada
	13C aDP	0.02	µg/ml	50	Wellington Laboratories, Canada
	13C sDP	0.02	µg/ml	50	Wellington Laboratories, Canada
	13C6 BTBPE	0.02	µg/ml	50	Wellington Laboratories, Canada
	13C6 d17-BEH-TEBP	0.02	µg/ml	50	Wellington Laboratories, Canada
	13C6 d17-EH-TBB	0.02	µg/ml	50	Wellington Laboratories, Canada
	13C14 DBDPE	0.03	µg/ml	50	Wellington Laboratories, Canada
N-PAHs	D9 2-Nitrofluorene	0.4	µg/ml	50	AccuStandard, US
	D9 3-Nitrofluoranthene	0.4	µg/ml	50	AccuStandard, US
	D11 6-Nitrochrysene	0.4	µg/ml	50	AccuStandard, US
	D7 1-Nitronaphthalene	0.4	µg/ml	50	AccuStandard, US
	D9 9-Nitroanthracene	0.4	µg/ml	50	AccuStandard, US
	D9 1-Nitropyrene	0.4	µg/ml	50	AccuStandard, US
O-PAHs	D11 6-Nitrobenzo[a]pyrene	0.4	µg/ml	50	AccuStandard, US
	D8 Dibenzofuran	0.4	µg/ml	50	Chiron, Norway
	D8 9,10-Anthraquinone	0.4	µg/ml	50	Chiron, Norway
	D8 9-Fluroenone	0.4	µg/ml	50	Chiron, Norway
IS for GC compou	*13C12 PCB 95*	*0.2*	*µg/ml*	*50*	*Cambridge Isotope Laboratories, US*
	13C12 BDE 77	*0.1*	*µg/ml*	*10*	*Wellington Laboratories, Canada*
	p-terphenyl	*4.0*	*µg/ml*	*50*	*Sigma-Aldrich, US*

Table S3: List of compounds included in the method development and relevant physical-chemical properties. Physical-chemical properties were obtained from EPA CompTox Dashboard.

	Preferred name	CAS RN	INCHIKEY	IUPAC name	Abbreviation	$LogK_{OA}$	$LogK_{OW}$	VP (Pa)	Phase
PAHs	Naphthalene	91-20-3	UFWIBTONFRDIAS-UHFFFAOYSA-N	Naphthalene	NAP	5.17	3.30	11.2	1, 2, 3
	2-Methylnaphthalene	91-57-6	QIMMUPPBPVKWKM-UHFFFAOYSA-N	2-Methylnaphthalene		5.83	3.86	7.19	2
	1-Methylnaphthalene	90-12-0	QPUYECUOLPXSFR-UHFFFAOYSA-N	1-Methylnaphthalene		5.01	3.87	8.90	2
	Biphenyl	92-52-4	ZUOUZKKEUPVFJK-UHFFFAOYSA-N	1,1'-Biphenyl	BIP	6.14	4.01	1.19	1, 2, 3
	Acenaphthylene	208-96-8	HXGDTGSAIMULJN-UHFFFAOYSA-N	Acenaphthylene	ACY	6.56	3.94	0.88	1, 2, 3
	Acenaphthene	83-32-9	CWRYPZZKDGJXCA-UHFFFAOYSA-N	1,2-Dihydroacenaphthylene	ACE	6.33	3.92	0.29	1, 2, 3
	Fluorene	86-73-7	NIHNNTQXNPWCJQ-UHFFFAOYSA-N	9H-Fluorene	FLU	6.84	4.18	8.02E-02	1, 2, 3
	Dibenzothiophene	132-65-0	IYYZUPMFVPLQIF-UHFFFAOYSA-N	Dibenzo[b,d]thiophene		7.50	4.44	2.73E-02	2
	Phenanthrene	85-01-8	YNPNZTXNASCQKK-UHFFFAOYSA-N	Phenanthrene	PHEN	7.55	4.47	1.49E-02	1, 2, 3
	Anthracene	120-12-7	MWPLVEDNUUSJAV-UHFFFAOYSA-N	Anthracene	ANTH	7.55	4.45	9.46E-04	1, 2, 3
	3-Methylphenanthrene	832-71-3	GKYWZUBZZBHZKU-UHFFFAOYSA-N	3-Methylphenanthrene		8.41	5.14	8.61E-04	2
	2-Methylphenanthrene	2531-84-2	KANLOADZXMMCQA-UHFFFAOYSA-N	2-Methylphenanthrene		8.41	4.87	8.64E-04	2
	2-Methylanthracene	613-12-7	GYMFBYTZOGMSQJ-UHFFFAOYSA-N	2-Methylanthracene		8.41	5.00	7.14E-04	2
	9-Methylphenanthrene	883-20-5	DALBHIYZSZZWBS-UHFFFAOYSA-N	9-Methylphenanthrene		8.41	5.18	1.08E-03	2
	1-Methyl phenanthrene	832-69-9	DOWJXOHBNXRUOD-UHFFFAOYSA-N	1-Methylphenanthrene		7.55	5.08	2.02E-03	2
	Fluoranthene	206-44-0	GVEPBJHOBDJJJI-UHFFFAOYSA-N	Fluoranthene	FLTH	8.86	5.16	1.21E-03	1, 2, 3
	Pyrene	129-00-0	BBEAQIROQSPTKN-UHFFFAOYSA-N	Pyrene	PYR	8.86	4.88	5.97E-04	1, 2, 3
	11H-Benzo[a]fluorene	238-84-6	HKMTVMBEALTRRR-UHFFFAOYSA-N	11H-Benzo[a]fluorene		7.88	5.54	3.24E-05	2
	(1H)-Benzo(b)fluorene	14458-76-5	RZKOXMMPOZMIOS-UHFFFAOYSA-N	1H-Benzo[b]fluorene		8.86	5.10	3.02E-05	1, 2, 3

	Preferred name	CAS RN	INCHIKEY	IUPAC name	Abbreviation	LogK_{OA}	LogK_{OW}	VP (Pa)	Phase
	Retene	483-65-8	NXLOLUFNDSBYTP-UHFFFAOYSA-N	1-Methyl-7-(propan-2-yl)phenanthrene	RET	8.53	5.54	1.24E-04	1, 2, 3
	Benzo[b]naphtho[2,1-d]thiophene	239-35-0	YEUHHUCOSQOCIX-UHFFFAOYSA-N	Benzo[b]naphtho[2,1-d]thiophene		8.86	5.51	1.53E-05	1, 3
	Benzo[ghi]fluoranthene	203-12-3	YEIHPPOCKIHUQJ-UHFFFAOYSA-N	Benzo[ghi]fluoranthene	B[ghi]F	9.92	5.70	2.87E-06	1, 2, 3
	Cyclopenta[cd]pyrene	27208-37-3	BZCXQYVNASLLQO-UHFFFAOYSA-N	Cyclopenta[cd]pyrene	C[cd]P	9.63	5.70	2.95E-06	1, 2, 3
	Benz(a)anthracene	56-55-3	DXBHBZVCASKNBY-UHFFFAOYSA-N	Tetraphene	B[a]A	9.37	5.60	2.47E-05	1, 2, 3
	Triphenylene	217-59-4	SLGBZMMZGDRARJ-UHFFFAOYSA-N	Triphenylene	TRI	9.37	5.49	2.80E-06	1, 2, 3
	Chrysene	218-01-9	WDECIBYCCFPHNR-UHFFFAOYSA-N	Chrysene	CHRY	9.37	5.81	8.89E-07	1, 2, 3
	Benzo(b)fluoranthene	205-99-2	FTOVXSOBNPWTSH-UHFFFAOYSA-N	Benzo[e]acephenanthrylene	B[b]F	8.64	5.78	6.57E-05	1, 2, 3
	Benzo[k]fluoranthene	207-08-9	HAXBIWFMXWRORI-UHFFFAOYSA-N	Benzo[k]fluoranthene	B[k]F	9.38	6.11	1.30E-07	1, 2, 3
	Benzo(j)fluoranthene	205-82-3	KHNYNFUTFKJLDD-UHFFFAOYSA-N	Benzo[j]fluoranthene	B[j]F	10.34	6.30	4.99E-07	1, 2, 3
	Benzo[a]fluoranthene	203-33-8	OQDXASJSCOTNQS-UHFFFAOYSA-N	Benzo[a]aceanthrylene	B[a]F	10.19	5.39	1.80E-06	2
	Benzo[e]pyrene	192-97-2	TXVHTIQJNYSSKO-UHFFFAOYSA-N	Benzo[e]pyrene	B[e]P	10.34	6.43	7.58E-07	1, 2, 3
	Benzo[a]pyrene	50-32-8	FMMWHPNWAFZXNH-UHFFFAOYSA-N	Benzo[pqr]tetraphene	B[a]P	9.61	6.13	7.35E-07	1, 2, 3
	Perylene	198-55-0	CSHWQDPOILHKBI-UHFFFAOYSA-N	Perylene	PER	10.34	6.05	6.99E-07	1, 2, 3
	Indeno[1,2,3-cd]pyrene	193-39-5	SXQBHARYMNFBPS-UHFFFAOYSA-N	Indeno[1,2,3-cd]pyrene	IND	11.72	6.76	3.09E-08	1, 2, 3
	Dibenz[a,c]anthracene	215-58-7	RAASUWZPTOJQAY-UHFFFAOYSA-N	Benzo[f]tetraphene	D[a,c]A	11.69	6.43	1.35E-08	1, 2, 3
	Dibenz[a,h]anthracene	53-70-3	LHRCREOYAASXPZ-UHFFFAOYSA-N	Benzo[k]tetraphene	D[a,h]A	11.69	6.72	1.27E-07	1, 2, 3
	Benzo[g,h,i]perylene	191-24-2	GYFAGKUZYNFMBN-UHFFFAOYSA-N	Benzo[ghi]perylene	B[ghi]P	11.72	6.63	1.33E-08	1, 2, 3
	Anthanthrene	191-26-4	YFIJJNAKSZUOLT-UHFFFAOYSA-N	Naphtho[7,8,1,2,3-nopqr]tetraphene		11.72	7.04	2.82E-08	1, 2, 3
	Coronene	191-07-1	VPUGDVKSAQVFFS-UHFFFAOYSA-N	Coronene	COR	11.69	7.64	2.90E-10	1, 2, 3

	Preferred name	CAS RN	INCHIKEY	IUPAC name	Abbreviation	$LogK_{OA}$	$LogK_{OW}$	VP (Pa)	Phase
	Dibenzo(a,e)pyrene	192-65-4	KGHMWBNEMFNJFZ-UHFFFAOYSA-N	Dibenzo[f,pqr]tetraphene	D[a,e]P	11.76	6.96	1.05E-08	2
	Dibenzo[a,i]pyrene	189-55-9	TUGYIJVAYAHHHM-UHFFFAOYSA-N	Benzo[rst]pentaphene	D[a,i]P	11.69	7.39	5.41E-09	2
	Dibenzo[a,h]pyrene	189-64-0	RXUSYFJGDZFVND-UHFFFAOYSA-N	Dibenzo[c,pqr]tetraphene	D[a,h]P	11.69	7.39	5.41E-09	2
N-PAHs	1-Nitronaphthalene	86-57-7	RJKGJBPXVHTNJL-UHFFFAOYSA-N	1-Nitronaphthalene		5.77	3.17	6.35E-02	1, 3
	2-Nitronaphthalene	581-89-5	ZJYJZEAJZXVAMF-UHFFFAOYSA-N	2-Nitronaphthalene		6.08	3.24	5.64E-04	1, 3
	Acenaphthene, 3-nitro-	3807-77-0	WTRQPBAHQILXND-UHFFFAOYSA-N	3-Nitro-1,2-dihydroacenaphthylene		6.90	2.84	7.82E-05	1, 3
	5-Nitroacenaphthene	602-87-9	CUARLQDWYSRQDF-UHFFFAOYSA-N	5-Nitro-1,2-dihydroacenaphthylene		7.46	3.85	9.78E-05	1, 3
	2-Nitrofluorene	607-57-8	XFOHWECQTFIEIX-UHFFFAOYSA-N	2-Nitro-9H-fluorene		7.55	3.38	3.83E-04	1, 3
	9-Nitroanthracene	602-60-8	LSIKFJXEYJIZNB-UHFFFAOYSA-N	9-Nitroanthracene		7.37	4.78	1.25E-04	1, 2, 3
	9-Nitrophenanthrene	954-46-1	QTTCNQHPKFAYEZ-UHFFFAOYSA-N	9-Nitrophenanthrene		8.02	4.02	8.91E-05	1, 3
	Phenanthrene, 3-nitro-	17024-19-0	CPRHWWUDRYJODK-UHFFFAOYSA-N	3-Nitrophenanthrene		8.02	4.04	5.89E-05	1, 3
	Fluoranthene, 2-nitro-	13177-29-2	VBCBFNMZBHKVQN-UHFFFAOYSA-N	2-Nitrofluoranthene		9.32	4.71	4.39E-06	1, 2, 3
	3-Nitrofluoranthene	892-21-7	PIHGQKMEAMSUNA-UHFFFAOYSA-N	3-Nitrofluoranthene		4.61	3.98	1.15E-05	1, 2, 3
	1-Nitropyrene	5522-43-0	ALRLPDGCPYIVHP-UHFFFAOYSA-N	1-Nitropyrene		9.32	5.06	4.39E-06	1, 2, 3
	2-Nitropyrene	789-07-1	MAZCGYFIOOIVHE-UHFFFAOYSA-N	2-Nitropyrene		9.32	4.70	4.36E-06	1, 3
	4-Nitropyrene	57835-92-4	UISKIUIWPSPSAV-UHFFFAOYSA-N	4-Nitropyrene		9.32	4.92	4.40E-06	2
	Benz[a]anthracene, 7-nitro-	20268-51-3	KOPVBVBUIYTJBG-UHFFFAOYSA-N	7-Nitrotetraphene		9.40	4.01	8.26E-07	1, 2, 3
	3-Nitrobenzanthrone	17117-34-9	QAJOWHGESRCVLY-UHFFFAOYSA-N	3-Nitro-7H-benzo[de]anthracen-7-one		9.88	4.24	3.10E-08	1, 2, 3
	6-Nitrochrysene	7496-02-8	UAWLTQJFZUYROA-UHFFFAOYSA-N	6-Nitrochrysene		10.57	5.17	7.81E-07	1, 3
	1,3-Dinitropyrene	75321-20-9	KTNUVDBUEAQUON-UHFFFAOYSA-N	1,3-Dinitropyrene		10.59	4.12	1.27E-07	1, 2, 3

	Preferred name	CAS RN	INCHIKEY	IUPAC name	Abbreviation	LogK$_{OA}$	LogK$_{OW}$	VP (Pa)	Phase
	1,6-Dinitropyrene	42397-64-8	GUXACCKTQWVTLG-UHFFFAOYSA-N	1,6-Dinitropyrene		10.61	4.25	1.30E-07	1, 2, 3
	1,8-Dinitropyrene	42397-65-9	BLYXNIHKOMELAP-UHFFFAOYSA-N	1,8-Dinitropyrene		10.61	4.27	1.29E-07	1, 3
	Benzo[a]pyrene, 6-nitro-	63041-90-7	NMMAFYSZGOFZCM-UHFFFAOYSA-N	6-Nitrobenzo[pqr]tetraphene		11.70	5.81	1.06E-07	1, 3
O-PAHs	1,4-Naphthoquinone	130-15-4	FRASJONUBLZVQX-UHFFFAOYSA-N	Naphthalene-1,4-dione		5.72	1.71	2.42E-02	1, 3
	1-Naphthaldehyde	66-77-3	SQAINHDHICKHLX-UHFFFAOYSA-N	Naphthalene-1-carbaldehyde		5.78	2.57	3.36E-01	1, 3
	Dibenzofuran	132-64-9	TXCDCPKCNAJMEE-UHFFFAOYSA-N	Dibenzo[b,d]furan		6.90	4.12	3.27E-01	1, 3
	9-Fluorenone	486-25-9	YLQWCDOCJODRMT-UHFFFAOYSA-N	9H-Fluoren-9-one		7.51	3.58	4.38E-03	1, 2, 3
	6H-Dibenzo-(b,d)-pyran-6-one	2005-10-9	TVKNXKLYVUVOCV-UHFFFAOYSA-N	6H-Dibenzo[b,d]pyran-6-one		8.34	3.11	1.63E-05	1, 3
	Anthraquinone	84-65-1	RZVHIXYEVGDQDX-UHFFFAOYSA-N	Anthracene-9,10-dione		8.41	3.39	1.55E-05	1, 2, 3
	9,10-Phenanthrenedione	84-11-7	YYVYAPXYZVYDHN-UHFFFAOYSA-N	Phenanthrene-9,10-dione		8.51	2.52	9.77E-06	1, 3
	11H-Benzo[a]fluoren-11-one	479-79-8	RNICURKFVSAHLQ-UHFFFAOYSA-N	11H-Benzo[a]fluoren-11-one		9.69	4.75	2.71E-06	1, 3
	11H-Benzo(b)fluoren-11-one	3074-03-1	MLMNDNOSVOKYMT-UHFFFAOYSA-N	11H-Benzo[b]fluoren-11-one		9.69	4.72	2.71E-06	1, 3
	Benz[de]anthracen-7-one	82-05-3	HUKPVYBUJRAUAG-UHFFFAOYSA-N	7H-Benzo[de]anthracen-7-one		9.69	4.81	2.70E-06	1, 3
	7,12-Benz(a)anthraquinone	2498-66-0	LHMRXAIRPKSGDE-UHFFFAOYSA-N	Tetraphene-7,12-dione		9.74	4.40	2.01E-07	1, 2, 3
	5,12-Naphthacenedione	1090-13-7	LZPBKINTWROMEA-UHFFFAOYSA-N	Tetracene-5,12-dione		9.74	4.29	4.02E-07	1, 3
	6H-Benzo(cd)pyren-6-one	3074-00-8	CLIKSBRDCNSYNO-UHFFFAOYSA-N	6H-Benzo[cd]pyren-6-one		10.32	4.32	1.32E-06	1, 2, 3
	2-Methylanthraquinone	84-54-8	NJWGQARXZDRHCD-UHFFFAOYSA-N	2-Methylanthracene-9,10-dione		8.94	3.34	1.07E-05	2
Musks	1,3,4,6,7,8-Hexahydro-4,6,6,7,8,8-hexamethylcyclopenta[g]-2-benzopyran	1222-05-5	ONKNPOPIGWHAQC-UHFFFAOYSA-N	4,6,6,7,8,8-Hexamethyl-1,3,4,6,7,8-hexahydroindeno[5,6-c]pyran	HHCB	8.18	5.90	7.21E-02	1, 3
	Tonalide	21145-77-7	DNRJTBAOUJJKDY-UHFFFAOYSA-N	1-(3,5,5,6,8,8-Hexamethyl-5,6,7,8-tetrahydronaphthalen-2-yl)ethan-1-one	AHTN	7.93	5.70	5.36E-03	1, 3
P [illegible]	Dimethyl phthalate	131-11-3	NIQCNGHVCWTJSM-UHFFFAOYSA-N	Dimethyl benzene-1,2-dicarboxylate	DMP	5.72	1.58	0.411	1, 3

	Preferred name	CAS RN	INCHIKEY	IUPAC name	Abbreviation	$LogK_{OA}$	$LogK_{OW}$	VP (Pa)	Phase
	Diethyl phthalate	84-66-2	FLKPEMZONWLCSK-UHFFFAOYSA-N	Diethyl benzene-1,2-dicarboxylate	DEP	6.75	2.44	0.28	1, 2, 3
	Diisobutyl phthalate	84-69-5	MGWAVDBGNNKXQV-UHFFFAOYSA-N	Bis(2-methylpropyl) benzene-1,2-dicarboxylate	DIBP	8.21	4.11	4.95E-03	1, 2, 3
	Dibutyl 1,2-benzenedicarboxylate	84-74-2	DOIRQSBPFJWKBE-UHFFFAOYSA-N	Dibutyl benzene-1,2-dicarboxylate	DBP	8.84	4.61	2.69E-03	1, 2, 3
	Dihexyl phthalate	84-75-3	KCXZNSGUUQJJTR-UHFFFAOYSA-N	Dihexyl benzene-1,2-dicarboxylate	DHxP	9.71	6.81	1.86E-03	1, 2, 3
	Benzyl butyl phthalate	85-68-7	IRIAEXORFWYRCZ-UHFFFAOYSA-N	Benzyl butyl benzene-1,2-dicarboxylate	BBzP	9.83	4.82	1.09E-03	1, 2, 3
	Dipentyl phthalate	131-18-0	IPKKHRVROFYTEK-UHFFFAOYSA-N	Dipentyl benzene-1,2-dicarboxylate	DPNP	9.40	5.62	9.50E-04	1, 3
	Bis(4-methyl-2-pentyl) phthalate	84-63-9	UAFXUVUVOTXFND-UHFFFAOYSA-N	Bis(4-methylpentan-2-yl) benzene-1,2-dicarboxylate		9.81	5.36	9.44E-04	1, 3
	Di(2-methoxyethyl) phthalate	117-82-8	HSUIVCLOAAJSRE-UHFFFAOYSA-N	Bis(2-methoxyethyl) benzene-1,2-dicarboxylate	DMEP	8.02	1.35	8.33E-04	1, 3
	Dicyclohexyl phthalate	84-61-7	VOWAEIGWURALJQ-UHFFFAOYSA-N	Dicyclohexyl benzene-1,2-dicarboxylate	DCHP	11.02	5.83	1.16E-04	1, 2, 3
	1,2-Benzenedicarboxylic acid, dinonyl ester	84-76-4	DROMNWUQASBTFM-UHFFFAOYSA-N	Dinonyl benzene-1,2-dicarboxylate	DNP	11.69	7.72	8.61E-05	2
	1,2-Benzenedicarboxylic acid, bis(2-ethoxyethyl) ester	605-54-9	RMKYMNRQXYPJHL-UHFFFAOYSA-N	Bis(2-ethoxyethyl) benzene-1,2-dicarboxylate		9.17	2.47	8.11E-05	1, 3
	Bis(8-methylnonyl) phthalate	89-16-7	ZVFDTKUVRCTHQE-UHFFFAOYSA-N	Bis(8-methylnonyl) benzene-1,2-dicarboxylate	DIDP	11.69	8.90	7.03E-05	2
	Di(2-ethylhexyl) phthalate	117-81-7	BJQHLKABXJIVAM-UHFFFAOYSA-N	Bis(2-ethylhexyl) benzene-1,2-dicarboxylate	DEHP	11.69	7.52	1.90E-05	1, 2, 3
	Di-n-octyl phthalate	117-84-0	MQIUGAXCHLFZKX-UHFFFAOYSA-N	Dioctyl benzene-1,2-dicarboxylate	DNOP	11.74	8.10	1.34E-05	1, 2, 3
	Bis(2-butoxyethyl) phthalate	117-83-9	CMCJNODIWQEOAI-UHFFFAOYSA-N	Bis(2-butoxyethyl) benzene-1,2-dicarboxylate	DBEP	10.45	3.64	5.06E-06	1, 3
OPFRs	Triethyl phosphate	78-40-0	DQWPFSLDHJDLRL-UHFFFAOYSA-N	Triethyl phosphate	TEP	5.79	0.80	52.2	2
	Triisopropyl phosphate	513-02-0	OXFUXNFMHFCELM-UHFFFAOYSA-N	Tripropan-2-yl phosphate	TIPP	6.34	2.17	22.0	2
	Tris(2-chloroethyl) phosphate	115-96-8	HQUQLFOMPYWACS-UHFFFAOYSA-N	Tris(2-chloroethyl) phosphate	TCEP	8.41	1.44	8.12	2
	Tripropyl phosphate	513-08-6	RXPQRKFMDQNODS-UHFFFAOYSA-N	Tripropyl phosphate	TPrP	7.26	1.87	1.74	2
	Tris(2-chloroisopropyl)phosphate	13674-84-5	KVMPUXDNESXNOH-UHFFFAOYSA-N	Tris(1-chloropropan-2-yl) phosphate	TCIPP	8.85	2.59	0.50	2

	Preferred name	CAS RN	INCHIKEY	IUPAC name	Abbreviation	$LogK_{OA}$	$LogK_{OW}$	VP (Pa)	Phase
	Tributyl phosphate	126-73-8	STCOOQWBFONSKY-UHFFFAOYSA-N	Tributyl phosphate	TBP	7.88	4.00	0.15	2
	Triisobutyl phosphate	126-71-6	HRKAMJBPFPHCSD-UHFFFAOYSA-N	Tris(2-methylpropyl) phosphate	TiBP	7.38	3.15	1.20	2
	2-Ethylhexyl diphenyl phosphate	1241-94-7	CGSLYBDCEGBZCG-UHFFFAOYSA-N	2-Ethylhexyl diphenyl phosphate	EHDPP	11.73	5.73	6.64E-03	2
	Tris(2-butoxyethyl) phosphate	78-51-3	WTLBZVNBAKMVDP-UHFFFAOYSA-N	Tris(2-butoxyethyl) phosphate	TBOEP	11.73	3.75	2.28E-03	2
	Triphenyl phosphate	115-86-6	XZZNDPSIHUTMOC-UHFFFAOYSA-N	Triphenyl phosphate	TPHP	10.80	4.59	8.37E-04	2
	Tris(1,3-dichloro-2-propyl) phosphate	13674-87-8	ASLWPAWFJZFCKF-UHFFFAOYSA-N	Tris(1,3-dichloropropan-2-yl) phosphate	TDCIPP	10.27	3.65	7.01E-04	2
	Tris(2-ethylhexyl) phosphate	78-42-2	GTVWRXDRKAHEAD-UHFFFAOYSA-N	Tris(2-ethylhexyl) phosphate	TEHP	11.69	9.20	1.10E-05	2
	Diphenyl 4-isopropylphenyl phosphate	55864-04-5	JUHFQCKQQLMGAB-UHFFFAOYSA-N	Diphenyl 4-(propan-2-yl)phenyl phosphate	4-IPPDPP	11.71	5.27	3.76E-05	2
	Diphenyl o-isopropylphenylphenyl phosphate	64532-94-1	JJXNVYMIYBNZQX-UHFFFAOYSA-N	Diphenyl 2-(propan-2-yl)phenyl phosphate	2-IPPDPP	11.71	5.26	8.85E-06	2
	Tris(4-methylphenyl) phosphate	78-32-0	BOSMZFBHAYFUBJ-UHFFFAOYSA-N	Tris(4-methylphenyl) phosphate	TCP	11.70	5.13	5.26E-06	2
	Tris(2,4-dimethylphenyl) phosphate	3862-12-2	KOWVWXQNQNCRRS-UHFFFAOYSA-N	Tris(2,4-dimethylphenyl) phosphate	TXP	11.68	5.57	5.46E-07	2
	Bis(4-isopropylphenyl) phenyl phosphate	55864-07-8	GQJZORIDXGBCCT-UHFFFAOYSA-N	Phenyl bis[4-(propan-2-yl)phenyl] phosphate	B4IPPPP	11.69	5.47	5.20E-07	2
NFRs	1,2-Dibromo-4-(1,2-dibromoethyl)cyclohexane	3322-93-8	PQRRSJBLKOPVJV-UHFFFAOYSA-N	1,2-Dibromo-4-(1,2-dibromoethyl)cyclohexane	TBECH	8.42	5.15	14.2	1, 2, 3
	1,3,5-Tribromo-2-(prop-2-en-1-yloxy)benzene	3278-89-5	RZLLIOPGUFOWOD-UHFFFAOYSA-N	1,3,5-Tribromo-2-[(prop-2-en-1-yl)oxy]benzene	TBP-AE	7.82	4.85	0.49	1, 2, 3
	Benzene, pentabromo-	608-90-2	LLVVSBBXENOOQY-UHFFFAOYSA-N	1,2,3,4,5-Pentabromobenzene	PBBZ	7.93	4.73	0.26	1, 2, 3
	2,3,4,5,6-Pentabromoethylbenzene	85-22-3	FIAXCDIQXHJNIX-UHFFFAOYSA-N	1,2,3,4,5-Pentabromo-6-ethylbenzene	PBEB	8.83	5.62	0.13	1, 2, 3
	Pentabromotoluene	87-83-2	OZHJEQVYCBTHJT-UHFFFAOYSA-N	1,2,3,4,5-Pentabromo-6-methylbenzene	PBT	8.95	5.45	6.97E-02	1, 2, 3
	2-Bromoallyl 2,4,6-tribromophenyl ether	99717-56-3	RLPZXGWCSHFKJI-UHFFFAOYSA-N	1,3,5-Tribromo-2-[(2-bromoprop-2-en-1-yl)oxy]benzene	BATE	8.91	5.14	3.01E-02	1, 2, 3
	1,3,5-Tribromo-2-(2,3-dibromopropoxy)benzene	35109-60-5	QXWYPAKUEHGJSG-UHFFFAOYSA-N	1,3,5-Tribromo-2-(2,3-dibromopropoxy)benzene	TBP-DPTE	9.82	5.95	1.02E-03	1, 2, 3
	Hexabromobenzene	87-82-1	CAYGQBVSOZLICD-UHFFFAOYSA-N	Hexabromobenzene	HBB	9.26	6.07	4.39E-04	1, 2, 3

	Preferred name	CAS RN	INCHIKEY	IUPAC name	Abbreviation	$LogK_{OA}$	$LogK_{OW}$	VP (Pa)	Phase
	2-Ethylhexyl 2,3,4,5-tetrabromobenzoate	183658-27-7	HVDXCGSGEQKWGB-UHFFFAOYSA-N	2-Ethylhexyl 2,3,4,5-tetrabromobenzoate	EH-TBB	11.57	6.19	1.27E-04	1, 2, 3
	1,1'-Ethane-1,2-diylbis(pentabromobenzene)	84852-53-9	BZQKBFHEWDPQHD-UHFFFAOYSA-N	1,1'-(Ethane-1,2-diyl)bis(pentabromobenzene)	DBDPE	11.68	7.52	6.34E-06	1, 2, 3
	1,2-Bis(2,4,6-tribromophenoxy)ethane	37853-59-1	YATIGPZCMOYEGE-UHFFFAOYSA-N	1,1'-[Ethane-1,2-diylbis(oxy)]bis(2,4,6-tribromobenzene)	BTBPE	11.69	7.58	5.03E-06	1, 2, 3
	Bis(2-ethylhexyl) tetrabromophthalate	26040-51-7	UUEDINPOVKWVAZ-UHFFFAOYSA-N	Bis(2-ethylhexyl) 3,4,5,6-tetrabromobenzene-1,2-dicarboxylate	BEH-TEBP	11.68	7.55	6.71E-07	1, 2, 3
	2,3,4,5-Tetrabromo-6-chlorotoluene	39569-21-6	WMXWTOJJASZOCL-UHFFFAOYSA-N	1,2,3,4-Tetrabromo-5-chloro-6-methylbenzene	TBCT	8.21	5.33	1.56E-01	1, 3
	(Pentabromophenyl)methyl acrylate	59447-55-1	GRKDVZMVHOLESV-UHFFFAOYSA-N	(Pentabromophenyl)methyl prop-2-enoate	PBB-Acr	10.58	5.42	2.00E-04	1, 3
	1,3,5-Tris(2,3-dibromopropyl)-1,3,5-triazine-2,4,6(1H,3H,5H)-trione	52434-90-9	NZUPFZNVGSWLQC-UHFFFAOYSA-N	1,3,5-Tris(2,3-dibromopropyl)-1,3,5-triazinane-2,4,6-trione	TDBP-TAZTO	11.68	2.66	4.48E-06	1, 3
	2,3,5,6-Tetrabromo-p-xylene	23488-38-2	RXKOKVQKECXYOT-UHFFFAOYSA-N	1,2,4,5-Tetrabromo-3,6-dimethylbenzene	TBX	8.00	5.59	2.85	1, 3
	1,2,5,6-Tetrabromocyclooctane	3194-57-8	RZLXIANUDLLFHN-UHFFFAOYSA-N	1,2,5,6-Tetrabromocyclooctane	TBCO	8.42	5.14	10.0	1, 3
	7,8-Dibromo-1,2,3,4,11,11-hexachloro-1,4,4a,5,6,7,8,9,10,10a-decahydro-1,4-methanobenzocyclooctene	51936-55-1	XRFONNJUMOCNHA-UHFFFAOYSA-N	7,8-Dibromo-1,2,3,4,11,11-hexachloro-1,4,4a,5,6,7,8,9,10,10a-decahydro-1,4-methanobenzo[8]annulene	DBHCTD	11.75	6.06	2.52E-04	1, 3
PBDEs	2,4,4'-Tribromodiphenyl ether	41318-75-6	UPNBETHEXPIWQX-UHFFFAOYSA-N	2,4-Dibromo-1-(4-bromophenoxy)benzene	PBDE 28	9.45	5.94	8.97E-05	1, 2, 3
	2,2',4,4'-Tetrabromodiphenyl ether	5436-43-1	XYBSIYMGXVUVGY-UHFFFAOYSA-N	1,1'-Oxybis(2,4-dibromobenzene)	PBDE 47	10.53	6.81	9.38E-06	1, 2, 3
	2,2',4,4',5,5'-Hexabromodiphenyl ether	68631-49-2	RZXIRSKYBISPGF-UHFFFAOYSA-N	1,1'-Oxybis(2,4,5-tribromobenzene)	PBDE 153	11.71	7.90	8.50E-06	1, 2, 3
	2,2',4,4',5,6'-Hexabromodiphenyl ether	207122-15-4	VHNPZYZQKWIWOD-UHFFFAOYSA-N	1,3,5-Tribromo-2-(2,4,5-tribromophenoxy)benzene	PBDE 154	11.71	7.82	8.48E-06	1, 2, 3
	2,2',3,4,4',5',6-Heptabromodiphenyl ether	207122-16-5	ILPSCQCLBHQUEM-UHFFFAOYSA-N	1,2,3,5-Tetrabromo-4-(2,4,5-tribromophenoxy)benzene	PBDE 183	11.69	8.27	5.10E-06	1, 2, 3
	2,2',4,4',5-Pentabromodiphenyl ether	60348-60-9	WHPVYXDFIXRKLN-UHFFFAOYSA-N	1,2,4-Tribromo-5-(2,4-dibromophenoxy)benzene	PBDE 99	11.35	7.32	4.13E-06	1, 2, 3
	2,2',4,4',6-Pentabromodiphenyl ether	189084-64-8	NSKIRYMHNFTRLR-UHFFFAOYSA-N	1,3,5-Tribromo-2-(2,4-dibromophenoxy)benzene	PBDE 100	11.54	7.24	3.92E-06	1, 2, 3
	2,3',4,4'-Tetrabromodiphenyl ether	189084-61-5	DHUMTYRHKMCVAG-UHFFFAOYSA-N	1,2-Dibromo-4-(2,4-dibromophenoxy)benzene	PBDE 66	10.53	6.48	1.43E-05	1, 3
	2,2',3,4,4'-Pentabromodiphenyl ether	182346-21-0	DMLQSUZPTTUUDP-UHFFFAOYSA-N	1,2,3-Tribromo-4-(2,4-dibromophenoxy)benzene	PBDE 85	11.65	7.37	9.72E-07	1, 3

	Preferred name	CAS RN	INCHIKEY	IUPAC name	Abbreviation	$LogK_{OA}$	$LogK_{OW}$	VP (Pa)	Phase
	2,2',3,3',4,4',5,5',6,6'-Decabromodiphenyl ether	1163-19-5	WHHGLZMJPXIBIX-UHFFFAOYSA-N	1,1'-Oxybis(pentabromobenzene)	PBDE 209	11.68	7.74	1.13E-08	1, 3
PCBs	2,5-Dichlorobiphenyl	34883-39-1	KKQWHYGECTYFIA-UHFFFAOYSA-N	2,5-Dichloro-1,1'-biphenyl	PCB 9	7.42	5.13	0.18	1, 3
	3,3'-Dichlorobiphenyl	2050-67-1	KTXUOWUHFLBZPW-UHFFFAOYSA-N	3,3'-Dichloro-1,1'-biphenyl	PCB 11	7.42	5.28	8.69E-02	1, 3
	2,4,4'-Trichlorobiphenyl	7012-37-5	BZTYNSQSZHARAZ-UHFFFAOYSA-N	2,4,4'-Trichloro-1,1'-biphenyl	PCB 28	7.78	5.62	1.73E-02	1, 3
	2,2',5,5'-Tetrachlorobiphenyl	35693-99-3	HCWZEPKLWVAEOV-UHFFFAOYSA-N	2,2',5,5'-Tetrachloro-1,1'-biphenyl	PCB 52	8.49	6.18	3.39E-03	1, 3
	2,2',4,5,5'-Pentachlorobiphenyl	37680-73-2	LAHWLEDBADHJGA-UHFFFAOYSA-N	2,2',4,5,5'-Pentachloro-1,1'-biphenyl	PCB 101	8.96	6.10	1.61E-03	1, 3
	2,3',4,4',5-Pentachlorobiphenyl	31508-00-6	IUTPYMGCWINGEY-UHFFFAOYSA-N	2,3',4,4',5-Pentachloro-1,1'-biphenyl	PCB 118	9.24	7.11	4.11E-04	1, 3
	2,2',3,4,4',5'-Hexachlorobiphenyl	35065-28-2	RPUMZMSNLZHIGZ-UHFFFAOYSA-N	2,2',3,4,4',5'-Hexachloro-1,1'-biphenyl	PCB 139	9.80	7.34	2.82E-04	1, 3
	2,2',4,4',5,5'-Hexachlorobiphenyl	35065-27-1	MVWHGTYKUMDIHL-UHFFFAOYSA-N	2,2',4,4',5,5'-Hexachloro-1,1'-biphenyl	PCB 153	9.73	6.56	1.83E-04	1, 3
	2,2',3,4,4',5,5'-Heptachlorobiphenyl	35065-29-3	WBHQEUPUMONIKF-UHFFFAOYSA-N	2,2',3,4,4',5,5'-Heptachloro-1,1'-biphenyl	PCB 180	9.94	7.72	5.63E-05	1, 3
OCPs	Pentachlorobenzene	608-93-5	CEOCDNVZRAIOQZ-UHFFFAOYSA-N	1,2,3,4,5-Pentachlorobenzene	PECB	6.49	5.17	0.14	1, 3
	alpha-1,2,3,4,5,6-Hexachlorocyclohexane	319-84-6	JLYXXMFPNIAWKQ-SHFUYGGZSA-N	(1R,2R,3R,4R,5S,6S)-1,2,3,4,5,6-Hexachlorocyclohexane	A_HCH	8.09	3.72	5.66E-03	1, 3
	beta-Hexachlorocyclohexane	319-85-7	JLYXXMFPNIAWKQ-CDRYSYESSA-N	(1r,2r,3r,4r,5r,6r)-1,2,3,4,5,6-Hexachlorocyclohexane	B_HCH	8.09	3.72	5.66E-03	1, 3
	Lindane	58-89-9	JLYXXMFPNIAWKQ-GNIYUCBRSA-N	(1R,2S,3r,4R,5S,6r)-1,2,3,4,5,6-Hexachlorocyclohexane	G_HCH	8.09	3.72	5.66E-03	1, 3
	delta-Hexachlorocyclohexane	319-86-8	JLYXXMFPNIAWKQ-GPIVLXJGSA-N	(1R,2S,3r,4R,5S,6s)-1,2,3,4,5,6-Hexachlorocyclohexane	D_HCH	8.09	3.72	5.66E-03	1, 3
	epsilon-Hexachlorocyclohexane	6108-10-7	JLYXXMFPNIAWKQ-DCLYFUHFSA-N	(1R,2R,3s,4S,5S,6s)-1,2,3,4,5,6-Hexachlorocyclohexane	E_HCH	8.09	3.72	5.66E-03	1, 3
	Hexachlorobenzene	118-74-1	CKAPSXZOOQJIBF-UHFFFAOYSA-N	Hexachlorobenzene	HCB	7.37	5.73	2.43E-03	1, 3
	o,p'-DDE	3424-82-6	ZDYJWDIWLRZXDB-UHFFFAOYSA-N	1-Chloro-2-[2,2-dichloro-1-(4-chlorophenyl)ethenyl]benzene	OP_DDE	9.44	6.23	8.21E-04	1, 3
	p,p'-DDE	72-55-9	UCNVFOCBFJOQAL-UHFFFAOYSA-N	1,1'-(2,2-Dichloroethene-1,1-diyl)bis(4-chlorobenzene)	PP_DDE	9.44	6.73	7.95E-04	1, 3
	1-(2-Chlorophenyl)-1-(4-chlorophenyl)-2,2-dichloroethane	53-19-0	JWBOIMRXGHLCPP-UHFFFAOYSA-N	1-Chloro-2-[2,2-dichloro-1-(4-chlorophenyl)ethyl]benzene	OP_DDD	10.02	6.77	2.58E-04	1, 3

	Preferred name	CAS RN	INCHIKEY	IUPAC name	Abbreviation	LogK_{OA}	LogK_{OW}	VP (Pa)	Phase
	p,p'-DDD	72-54-8	AHJKRLASYNVKDZ-UHFFFAOYSA-N	1,1'-(2,2-Dichloroethane-1,1-diyl)bis(4-chlorobenzene)	PP_DDDD	10.02	6.13	1.80E-04	1, 3
	o,p'-DDT	789-02-6	CVUGPAFCQJIYDT-UHFFFAOYSA-N	1-Chloro-2-[2,2,2-trichloro-1-(4-chlorophenyl)ethyl]benzene	OP_DDT	9.57	6.92	1.79E-04	1, 3
	DDT	50-29-3	YVGGHNCTFXOJCH-UHFFFAOYSA-N	1,1'-(2,2,2-Trichloroethane-1,1-diyl)bis(4-chlorobenzene)	PP_DDT	9.57	6.91	2.17E-05	1, 3
CUPs	Fonofos	944-22-9	KVGLBTYUCJYMND-UHFFFAOYSA-N	O-Ethyl S-phenyl ethylphosphonodithioate		8.98	3.94	4.49E-02	1, 2, 3
	Terbufos	13071-79-9	XLNZEKHULJKQBA-UHFFFAOYSA-N	S-[(tert-Butylsulfanyl)methyl] O,O-diethyl phosphorodithioate		9.78	4.48	4.24E-02	1, 2, 3
	Disulfoton	298-04-4	DOFZAZXDOSGAJZ-UHFFFAOYSA-N	O,O-Diethyl S-[2-(ethylsulfanyl)ethyl] phosphorodithioate		9.36	4.02	1.31E-02	1, 2, 3
	Diazinon	333-41-5	FHIVAFMUCKRCQO-UHFFFAOYSA-N	O,O-Diethyl O-[6-methyl-2-(propan-2-yl)pyrimidin-4-yl] phosphorothioate		9.83	3.81	1.20E-02	1, 2, 3
	Fenitrothion	122-14-5	ZNOLGFHPUIJIMJ-UHFFFAOYSA-N	O,O-Dimethyl O-(3-methyl-4-nitrophenyl) phosphorothioate		8.87	3.30	7.04E-03	1, 2, 3
	Metolachlor	51218-45-2	WVQBLGZPHOPPFO-UHFFFAOYSA-N	2-Chloro-N-(2-ethyl-6-methylphenyl)-N-(1-methoxypropan-2-yl)acetamide		9.39	3.13	4.15E-03	1, 2, 3
	Pendimethalin	40487-42-1	CHIFOSRWCNZCFN-UHFFFAOYSA-N	3,4-Dimethyl-2,6-dinitro-N-(pentan-3-yl)aniline		10.20	5.20	3.99E-03	1, 2, 3
	Chlorpyrifos	2921-88-2	SBPBAQFWLVIOKP-UHFFFAOYSA-N	O,O-Diethyl O-(3,5,6-trichloropyridin-2-yl) phosphorothioate		10.57	4.96	2.71E-03	1, 2, 3
	Dimethoate	60-51-5	MCWXGJITAZMZEV-UHFFFAOYSA-N	O,O-Dimethyl S-[2-(methylamino)-2-oxoethyl] phosphorodithioate		9.92	0.78	2.50E-03	1, 2, 3
	Acetochlor	34256-82-1	VTNQPKFIQCLBDU-UHFFFAOYSA-N	2-Chloro-N-(ethoxymethyl)-N-(2-ethyl-6-methylphenyl)acetamide		9.72	3.03	2.05E-03	1, 2, 3
	Alachlor	15972-60-8	XCSGPAVHZFQHGE-UHFFFAOYSA-N	2-Chloro-N-(2,6-diethylphenyl)-N-(methoxymethyl)acetamide		9.72	3.52	1.84E-03	1, 2, 3
	Dimethachlor	50563-36-5	SCCDDNKJYDZXMM-UHFFFAOYSA-N	2-Chloro-N-(2,6-dimethylphenyl)-N-(2-methoxyethyl)acetamide		8.78	2.17	1.51E-03	1, 2, 3
	Pirimicarb	23103-98-2	YFGYUFNIOHWBOB-UHFFFAOYSA-N	2-(Dimethylamino)-5,6-dimethylpyrimidin-4-yl dimethylcarbamate		8.63	1.70	9.69E-04	1, 2, 3
	Methyl parathion	298-00-0	RLBIQVVOMOPOHC-UHFFFAOYSA-N	O,O-Dimethyl O-(4-nitrophenyl) phosphorothioate		8.21	2.86	4.70E-04	1, 2, 3
	Malathion	121-75-5	JXSJBGJIGXNWCI-UHFFFAOYSA-N	Diethyl 2-[(dimethoxyphosphorothioyl)sulfanyl]butanedioate		9.61	2.36	4.51E-04	1, 2, 3
	Azinphos-methyl	86-50-0	CJJOSEISRRTUQB-UHFFFAOYSA-N	O,O-Dimethyl S-[(4-oxo-1,2,3-benzotriazin-3(4H)-yl)methyl] phosphorodithioate		9.83	2.75	2.13E-04	1, 2, 3

	Preferred name	CAS RN	INCHIKEY	IUPAC name	Abbreviation	$LogK_{OA}$	$LogK_{OW}$	VP (Pa)	Phase
	1-Naphthalenol, 1-(N-methylcarbamate)	63-25-2	CVXBEEMKQHEXEN-UHFFFAOYSA-N	Naphthalen-1-yl methylcarbamate		9.13	2.36	1.82E-04	1, 2, 3
	Prochloraz	67747-09-5	TVLSRXXIMLFWEO-UHFFFAOYSA-N	N-Propyl-N-[2-(2,4,6-trichlorophenoxy)ethyl]-1H-imidazole-1-carboxamide		11.76	4.10	1.50E-04	1, 2, 3
	Terbutylazine	5915-41-3	FZXISNSWEXTPMF-UHFFFAOYSA-N	N~2~-tert-Butyl-6-chloro-N~4~-ethyl-1,3,5-triazine-2,4-diamine		8.42	3.21	1.47E-04	1, 2, 3
	Chloridazon	1698-60-8	WYKYKTKDBLFHCY-UHFFFAOYSA-N	5-Amino-4-chloro-2-phenylpyridazin-3(2H)-one		8.50	1.17	6.02E-05	1, 2, 3
	Metazachlor	67129-08-2	STEPQTYSZVCJPV-UHFFFAOYSA-N	2-Chloro-N-(2,6-dimethylphenyl)-N-[(1H-pyrazol-1-yl)methyl]acetamide		9.59	2.13	6.01E-05	1, 2, 3
	Metribuzin	21087-64-9	FOXFZRUHNHCZPX-UHFFFAOYSA-N	4-Amino-6-tert-butyl-3-(methylsulfanyl)-1,2,4-triazin-5(4H)-one		7.51	1.70	5.85E-05	1, 2, 3
	Propiconazole	60207-90-1	STJLVHWMYQXCPB-UHFFFAOYSA-N	1-{[2-(2,4-Dichlorophenyl)-4-propyl-1,3-dioxolan-2-yl]methyl}-1H-1,2,4-triazole		10.20	3.72	5.60E-05	1, 2, 3
	Atrazine	1912-24-9	MXWJVTOOROXGIU-UHFFFAOYSA-N	6-Chloro-N~2~-ethyl-N~4~-(propan-2-yl)-1,3,5-triazine-2,4-diamine		8.38	2.61	3.84E-05	1, 2, 3
	Diuron	330-54-1	XMTQQYYKAHVGBJ-UHFFFAOYSA-N	N'-(3,4-Dichlorophenyl)-N,N-dimethylurea		9.96	2.68	9.22E-06	1, 2, 3
	Chlorotoluron	15545-48-9	JXCGFZXSOMJFOA-UHFFFAOYSA-N	N'-(3-Chloro-4-methylphenyl)-N,N-dimethylurea		9.81	2.41	4.86E-06	1, 2, 3
	(2R,6S)-Fenpropimorph	67564-91-4	RYAUSSKQMZRMAI-ALOPSCKCSA-N	rel-(2R,6S)-4-[3-(4-tert-Butylphenyl)-2-methylpropyl]-2,6-dimethylmorpholine		8.85	4.93	4.01E-06	1, 2, 3
	Fenoxaprop-P-ethyl	71283-80-2	PQKBPHSEKWERTG-LLVKDONJSA-N	Ethyl (2R)-2-{4-[(6-chloro-1,3-benzoxazol-2-yl)oxy]phenoxy}propanoate		11.53	4.58	3.99E-06	1, 2, 3
	Isoproturon	34123-59-6	PUIYMUZLKQOUOZ-UHFFFAOYSA-N	N,N-Dimethyl-N'-[4-(propan-2-yl)phenyl]urea		9.80	2.69	3.31E-06	1, 2, 3
	Simazine	122-34-9	ODCWYMIRDDJXKW-UHFFFAOYSA-N	6-Chloro-N~2~,N~4~-diethyl-1,3,5-triazine-2,4-diamine		8.36	2.18	2.98E-06	1, 2, 3
	Temephos	3383-96-8	WWJZWCUNLNYYAU-UHFFFAOYSA-N	O,O,O',O'-Tetramethyl O,O'-[sulfanediyldi(4,1-phenylene)] bis(phosphorothioate)		11.68	5.96	2.21E-06	1, 2, 3
	Tebuconazole	107534-96-3	PXMNMQRDXWABCY-UHFFFAOYSA-N	1-(4-Chlorophenyl)-4,4-dimethyl-3-[(1H-1,2,4-triazol-1-yl)methyl]pentan-3-ol		10.65	3.70	1.72E-06	1, 2, 3
	Metamitron	41394-05-2	VHCNQEUWZYOAEV-UHFFFAOYSA-N	4-Amino-3-methyl-6-phenyl-1,2,4-triazin-5(4H)-one		7.72	0.83	8.55E-07	1, 2, 3
	Carbendazim	10605-21-7	TWFZGCMQGLPBSX-UHFFFAOYSA-N	Methyl 1H-benzimidazol-2-ylcarbamate		8.41	1.52	1.01E-07	1, 2, 3
	Fluroxypyr	69377-81-7	MEFQWPUMEMWTJP-UHFFFAOYSA-N	[(4-Amino-3,5-dichloro-6-fluoropyridin-2-yl)oxy]acetic acid		8.14	1.68	3.80E-09	1, 2, 3
P L	Pentafluoropropanoic anhydride	356-42-3	XETRHNFRKCNWAJ-UHFFFAOYSA-N	Pentafluoropropanoic anhydride	PFPA	2.19	2.73	16549	1, 2, 3

	Preferred name	CAS RN	INCHIKEY	IUPAC name	Abbreviation	$LogK_{OA}$	$LogK_{OW}$	VP (Pa)	Phase
	Perfluorobutanoic acid	375-22-4	YPJUNDFVDDCYIH-UHFFFAOYSA-N	Heptafluorobutanoic acid	PFBA	3.46	1.43	2.27	1, 2, 3
	Perfluorohexanoic acid	307-24-4	PXUULQAPEKKVAH-UHFFFAOYSA-N	Undecafluorohexanoic acid	PFHxA	3.83	2.85	120	1, 2, 3
	Perfluorooctanoic acid	335-67-1	SNGREZUHAYWORS-UHFFFAOYSA-N	Pentadecafluorooctanoic acid	PFOA	4.16	3.11	14.8	1, 2, 3
	Perfluoroheptanoic acid	375-85-9	ZWBAMYVPMDSJGQ-UHFFFAOYSA-N	Tridecafluoroheptanoic acid	PFHpA	4.09	2.06	8.90	1, 2, 3
	Perfluorononanoic acid	375-95-1	UZUFPBIDKMEQEQ-UHFFFAOYSA-N	Heptadecafluorononanoic acid	PFNA	4.20	3.54	1.12	1, 2, 3
	Perfluorodecanoic acid	335-76-2	PCIUEQPBYFRTEM-UHFFFAOYSA-N	Nonadecafluorodecanoic acid	PFDA	4.28	4.15	0.20	1, 3
	Perfluorotetradecanoic acid	376-06-7	RUDINRUXCKIXAJ-UHFFFAOYSA-N	Heptacosafluorotetradecanoic acid	PFTeDA	5.75	5.10	0.14	1, 3
	Perfluorotridecanoic acid	72629-94-8	LVDGGZAZAYHXEY-UHFFFAOYSA-N	Pentacosafluorotridecanoic acid	PFTrDA	4.96	5.35	8.81E-02	1, 2, 3
	Perfluoroundecanoic acid	2058-94-8	SIDINRCMMRKXGQ-UHFFFAOYSA-N	Henicosafluoroundecanoic acid	PFUnDA	4.70	4.00	8.64E-02	1, 2, 3
	Perfluorododecanoic acid	307-55-1	CXGONMQFMIYUJR-UHFFFAOYSA-N	Tricosafluorododecanoic acid	PFDoDA	4.75	5.24	6.33E-03	1, 2, 3
	Perfluorodecanesulfonic acid	335-77-3	HYWZIAVPBSTISZ-UHFFFAOYSA-N	Henicosafluorodecane-1-sulfonic acid	PFDS	5.76	5.58	1.09E-03	1, 3
	Perfluorooctanesulfonic acid	1763-23-1	YFSUTJLHUFNCNZ-UHFFFAOYSA-N	Heptadecafluorooctane-1-sulfonic acid	PFOS	4.75	5.61	3.30E-04	1, 2, 3
	Perfluoroheptanesulfonic acid	375-92-8	OYGQVDSRYXATEL-UHFFFAOYSA-N	Pentadecafluoroheptane-1-sulfonic acid	PFHpS	4.71	4.31	4.44E-05	1, 3
	Perfluorobutanesulfonic acid	375-73-5	JGTNAGYHADQMCM-UHFFFAOYSA-N	Nonafluorobutane-1-sulfonic acid	PFBS	4.16	3.12	1.51E-06	1, 2, 3
	Perfluorohexanesulfonic acid	355-46-4	QZHDEAJFRJCDMF-UHFFFAOYSA-N	Tridecafluorohexane-1-sulfonic acid	PFHxS	4.27	2.20	1.09E-06	1, 2, 3
	Perfluorooctanesulfonamide	754-91-6	RRRXPPIDPYTNJG-UHFFFAOYSA-N	1,1,2,2,3,3,4,4,5,5,6,6,7,7,8,8,8-Heptadecafluorooctane-1-sulfonamide	PFOSA	4.30	5.02	32.60	2
	6:2 Fluorotelomer sulfonate sodium salt	27619-94-9	CJZMVGPCWZSDSZ-UHFFFAOYSA-M	Sodium 3,3,4,4,5,5,6,6,7,7,8,8,8-tridecafluorooctane-1-sulfonate	6:2 FTS	5.71	1.18	1.10E-04	2
	8:2 Fluorotelomer sulfonic acid	39108-34-4	ALVYVCQIFHTIRD-UHFFFAOYSA-N	3,3,4,4,5,5,6,6,7,7,8,8,9,9,10,10,10-Heptadecafluorodecane-1-sulfonic acid	8:2 FTS	6.22	6.18	1.34E-03	2
Bisphenols	4-(1,1-Dimethylethyl)phenol	98-54-4	QHPQWRBYOIRBIT-UHFFFAOYSA-N	4-tert-Butylphenol		7.50	3.31	5.08	2
	4-Heptylphenol	1987-50-4	KNDDEFBFJLKPFE-UHFFFAOYSA-N	4-Heptylphenol		9.23	4.03	0.310	2

	Preferred name	CAS RN	INCHIKEY	IUPAC name	Abbreviation	$LogK_{OA}$	$LogK_{OW}$	VP (Pa)	Phase
	4-(2-Methylbutan-2-yl)phenol	80-46-6	NRZWYNLTFLDQQX-UHFFFAOYSA-N	4-(2-Methylbutan-2-yl)phenol		7.71	3.49	0.274	2
	4-Octylphenol	1806-26-4	NTDQQZYCCIDJRK-UHFFFAOYSA-N	4-Octylphenol		8.79	5.19	0.268	2
	4-Nonylphenol	104-40-5	IGFHQQFPSIBGKE-UHFFFAOYSA-N	4-Nonylphenol		9.36	5.76	0.109	2
	4-(1,1,3,3-Tetramethylbutyl)phenol	140-66-9	ISAVYTVYFVQUDY-UHFFFAOYSA-N	4-(2,4,4-Trimethylpentan-2-yl)phenol		8.69	4.87	6.49E-02	2
	4-Dodecylphenol	104-43-8	KJWMCPYEODZESQ-UHFFFAOYSA-N	4-Dodecylphenol		10.61	7.91	7.87E-03	2
	Triclosan	3380-34-5	XEFQLINVKFYRCS-UHFFFAOYSA-N	5-Chloro-2-(2,4-dichlorophenoxy)phenol		10.55	4.76	3.28E-05	2
	Bisphenol AF	1478-61-1	ZFVMWEVVKGLCIJ-UHFFFAOYSA-N	4,4'-(1,1,1,3,3,3-Hexafluoropropane-2,2-diyl)diphenol		8.13	4.02	1.02E-05	2
	Phenol, 2-[1-(4-hydroxyphenyl)-1-methylethyl]-	837-08-1	MLCQXUZZAXKTSG-UHFFFAOYSA-N	2-[2-(4-Hydroxyphenyl)propan-2-yl]phenol		8.38	3.39	1.02E-05	2
	Bisphenol A	80-05-7	IISBACLAFKSPIT-UHFFFAOYSA-N	4,4'-(Propane-2,2-diyl)diphenol	BPA	8.38	3.32	9.04E-06	2
	2,2'-Bisphenol F	2467-02-9	MQCPOLNSJCWPGT-UHFFFAOYSA-N	2,2'-Methylenediphenol		8.18	3.40	6.67E-06	2
	Bis(4-hydroxyphenyl)methane	620-92-8	PXKLMJQFEQBVLD-UHFFFAOYSA-N	4,4'-Methylenediphenol	BPF	8.18	2.91	6.49E-06	2
	2-[(4-Hydroxyphenyl)methyl]phenol	2467-03-0	LVLNPXCISNPHLE-UHFFFAOYSA-N	2-[(4-Hydroxyphenyl)methyl]phenol		8.18	3.15	6.35E-06	2
	Bisphenol B	77-40-7	HTVITOHKHWFJKO-UHFFFAOYSA-N	4,4'-(Butane-2,2-diyl)diphenol		8.99	3.77	5.16E-06	2
	3,3'-Dimethylbisphenol A	79-97-0	YMTYZTXUZLQUSF-UHFFFAOYSA-N	4,4'-(Propane-2,2-diyl)bis(2-methylphenol)		9.07	3.87	4.67E-06	2
	Bisphenol E	2081-08-5	HCNHNBLSNVSJTJ-UHFFFAOYSA-N	4,4'-(Ethane-1,1-diyl)diphenol		8.20	3.24	4.16E-06	2
	4,4'-(9H-Fluorene-9,9-diyl)diphenol	3236-71-3	YWFPGFJLYRKYJZ-UHFFFAOYSA-N	4,4'-(9H-Fluorene-9,9-diyl)diphenol		9.52	3.67	9.46E-07	2
	2,2-Bis(4-hydroxy-3-isopropylphenyl)propane	127-54-8	IJWIRZQYWANBMP-UHFFFAOYSA-N	4,4'-(Propane-2,2-diyl)bis[2-(propan-2-yl)phenol]		9.28	6.40	7.40E-07	2
	3,3',5,5'-Tetrabromobisphenol A	79-94-7	VEORPZCZECFIRK-UHFFFAOYSA-N	4,4'-(Propane-2,2-diyl)bis(2,6-dibromophenol)		9.34	6.66	7.07E-07	2
	BisOPP-A	24038-68-4	BKTRENAPTCBBFA-UHFFFAOYSA-N	5,5'-(Propane-2,2-diyl)di([1,1'-biphenyl]-2-ol)		9.60	6.88	4.72E-07	2

	Preferred name	CAS RN	INCHIKEY	IUPAC name	Abbreviation	$LogK_{OA}$	$LogK_{OW}$	VP (Pa)	Phase
	Bisphenol Z	843-55-0	SDDLEVPIDBLVHC-UHFFFAOYSA-N	2~3~,2~4~,2~5~,2~6~-Tetrahydro-2~2~H-[1~1~,2~1~:2~1~,3~1~-terphenyl]-1~4~,3~4~-diol		9.20	4.08	3.03E-07	2
	4,4'-Dihydroxytetraphenylmethane	1844-01-5	BATCUENAARTUKW-UHFFFAOYSA-N	4,4'-(Diphenylmethylene)diphenol		9.53	3.51	2.20E-07	2
	bisphenol TMC	129188-99-4	UMPGNGRIGSEMTC-UHFFFAOYSA-N	2~3~,2~3~,2~5~-Trimethyl-2~3~,2~4~,2~5~,2~6~-tetrahydro-2~2~H-[1~1~,2~1~:2~1~,3~1~-terphenyl]-1~4~,3~4~-diol		9.31	6.08	8.54E-08	2
	Bisphenol P	2167-51-3	GIXXQTYGFOHYPT-UHFFFAOYSA-N	4,4'-[1,4-Phenylenedi(propane-2,2-diyl)]diphenol		9.45	6.11	3.32E-08	2
	1,3-Bis[1-methyl-1-(4-hydroxyphenyl)ethyl]benzene	13595-25-0	PVFQHGDIOXNKIC-UHFFFAOYSA-N	4,4'-[1,3-Phenylenedi(propane-2,2-diyl)]diphenol		9.45	6.11	3.22E-08	2
	4,4'-(1-Phenylethylidene)bisphenol	1571-75-1	VOWWYDCFAISREI-UHFFFAOYSA-N	4,4'-(1-Phenylethane-1,1-diyl)diphenol		9.30	3.71	2.76E-08	2
	4,4'-Sulfonyldiphenol	80-09-1	VPWNQTHUCYMVMZ-UHFFFAOYSA-N	4,4'-Sulfonyldiphenol	BPS	8.98	-0.10	2.60E-08	2
	2,4'-Dihydroxydiphenyl sulfone	5397-34-2	LROZSPADHSXFJA-UHFFFAOYSA-N	2-(4-Hydroxybenzene-1-sulfonyl)phenol		8.42	-0.08	2.52E-08	2
BADGE	Bisphenol A diglycidyl ether	1675-54-3	LCFVJGUPQDGYKZ-UHFFFAOYSA-N	2,2'-{Propane-2,2-diylbis[(4,1-phenylene)oxymethylene]}bis(oxirane)	BADGE	10.47	3.36	1.13E-04	2
	BADGE-HCl	13836-48-1	HLLOKZYCSSQYEB-UHFFFAOYSA-N	1-Chloro-3-[4-(2-{4-[(oxiran-2-yl)methoxy]phenyl}propan-2-yl)phenoxy]propan-2-ol	BADGE-HCl	10.45	4.21	2.86E-05	2
	1,1'-{Propane-2,2-diylbis[(4,1-phenylene)oxy]}bis(3-chloropropan-2-ol)	4809-35-2	PTCFDJRJOGPUFE-UHFFFAOYSA-N	1,1'-{Propane-2,2-diylbis[(4,1-phenylene)oxy]}bis(3-chloropropan-2-ol)	BADGE-2HCl	9.49	5.54	4.87E-07	2
	3-[4-(2-{4-[(Oxiran-2-yl)methoxy]phenyl}propan-2-yl)phenoxy]propane-1,2-diol	76002-91-0	NBLIPZBCGXIEFO-UHFFFAOYSA-N	3-[4-(2-{4-[(Oxiran-2-yl)methoxy]phenyl}propan-2-yl)phenoxy]propane-1,2-diol	BADGE-H2O	9.43	3.86	4.44E-07	2
	3,3'-{Methylenebis[(4,1-phenylene)oxy]}di(propane-1,2-diol)	72406-26-9	VGJRHUPDMVWBFM-UHFFFAOYSA-N	3,3'-{Methylenebis[(4,1-phenylene)oxy]}di(propane-1,2-diol)	BFDGE-2H2O	9.42	1.32	1.58E-07	2
	3,3'-{Propane-2,2-diylbis[(4,1-phenylene)oxy]}di(propane-1,2-diol)	5581-32-8	NISVZEWKUNUGQQ-UHFFFAOYSA-N	3,3'-{Propane-2,2-diylbis[(4,1-phenylene)oxy]}di(propane-1,2-diol)	BADGE-2H2O	9.42	2.16	1.03E-07	2
	3,3'-{Methylenebis[(4,1-phenylene)oxy]}bis(2-chloropropan-1-ol)	374772-79-9	VAFXFEBPCUMOIF-UHFFFAOYSA-N	3,3'-{Methylenebis[(4,1-phenylene)oxy]}bis(2-chloropropan-1-ol)	BFDGE-2HCl	9.46	4.00	5.60E-08	2
	Bis[4-(glycidyloxy)phenyl]methane	2095-03-6	XUCHXOAWJMEFLF-UHFFFAOYSA-N	2,2'-{Methylenebis[(4,1-phenylene)oxymethylene]}bis(oxirane)	BFDGE	10.57	3.38	1.11E−04	2
HB	(+/-)-α-Hexabromocyclododecane	134237-50-6	DEIGXXQKDWULML-PQTSNVLCSA-N	(1R,2R,5S,6R,9R,10S)-1,2,5,6,9,10-Hexabromocyclododecane	A_HBCD	11.68	5.92	6.28E-05	2

	Preferred name	CAS RN	INCHIKEY	IUPAC name	Abbreviation	$LogK_{OA}$	$LogK_{OW}$	VP (Pa)	Phase
	(+/-)-beta-Hexabromocyclododecane	134237-51-7	DEIGXXQKDWULML-UFVWWTPHSA-N	(1R,2R,5R,6S,9R,10S)-1,2,5,6,9,10-Hexabromocyclododecane	B_HBCD	11.68	5.92	6.28E-05	2
	(+/-)-gamma-Hexabromocyclododecane	134237-52-8	DEIGXXQKDWULML-MOCCIAMBSA-N	(1R,2R,5R,6S,9S,10R)-1,2,5,6,9,10-Hexabromocyclododecane	G_HBCD	11.68	5.92	6.28E-05	2
CP	1,5,5,6,6,10-Hexachlorodecane	90943-97-8	WFXBVJBVCUQCGH-UHFFFAOYSA-N	1,5,5,6,6,10-Hexachlorodecane	HCD	8.64	4.98	4.82E-02	2
Dechloranes	Dechlorane 604 Component B	NOCAS_1017197	YNUQMMCFLGZDJS-XBZVRZDZSA-N	(1R,4S,5R)-1,2,3,4,7,7-Hexachloro-5-(2,4,5-tribromophenyl)bicyclo[2.2.1]hept-2-ene		11.69	7.54	3.91E-04	2
	Dechloran 604	71245-27-7	PHYHWKJTYWPNCS-PDPXOMPHSA-N	(1R,4S,5R)-1,2,3,4,7,7-Hexachloro-5-(2,3,4,5-tetrabromophenyl)bicyclo[2.2.1]hept-2-ene	DC-604	11.68	7.67	5.63E-05	2
	syn-Dechlorane Plus	135821-03-3	UGQQAJOWXNCOPY-MXYLTYEXSA-N	(1R,4S,4aS,6aR,7R,10S,10aS,12aR)-1,2,3,4,7,8,9,10,13,13,14,14-Dodecachloro-1,4,4a,5,6,6a,7,10,10a,11,12,12a-dodecahydro-1,4:7,10-dimethanodibenzo[a,e][8]annulene	Syn-DP	11.68	6.37	6.26E-08	1, 2, 3
	anti-Dechlorane Plus	135821-74-8	UGQQAJOWXNCOPY-VBCJEVMVSA-N	(1R,4S,4aS,6aS,7S,10R,10aR,12aR)-1,2,3,4,7,8,9,10,13,13,14,14-Dodecachloro-1,4,4a,5,6,6a,7,10,10a,11,12,12a-dodecahydro-1,4:7,10-dimethanodibenzo[a,e][8]annulene	Anti-DP	11.68	6.37	6.26E-08	1, 2, 3
	Dechlorane 602	31107-44-5	CDBKZZACXQLFMK-DSCPOPHVSA-N	(1S,4R,4aR,5aS,6R,9S,9aR,9bS)-1,2,3,4,6,7,8,9,10,10,11,11-Dodecachloro-1,4,4a,5a,6,9,9a,9b-octahydro-1,4:6,9-dimethanodibenzo[b,d]furan	DC-602	15.00	8.36	NA	2
	Dechlorane 603	13560-92-4	WSEXCYJMFQVEIN-UUMMPQHJSA-N	(3S,6R,7S,9R)-3,4,5,6,10,11,12,13,16,16,17,17-dodecachlorohexacyclo[6.6.1.13,6.110,13.02,7.09,14]heptadeca-4,11-diene	DC-603	15.20	8.24	NA	2
	Dechlorane 601	13560- 90-2	COOHPPAZRZIWHM-UHFFFAOYSA-N	4,5,6,7,13,14,15,16,19,19,20,20-dodecachloroheptacyclo[9.6.1.14,7.113,16.02,10.03,8.012,17]icosa5,14-diene	DC-601	16.70	8.00	NA	2

Text S1 Instrumental analysis at RECETOX laboratories

PAHs + Musks

p-terphenyl was used as the internal standard and added after the final volume reduction. PAHs and musks were analyzed using an Agilent 8890A GC (Agilent Technologies, Inc., Santa Clara, CA, USA) equipped with a 60 m × 0.25 mm × 0.25 µm Rxi-5Sil-MS capillary column (Restek, Inc., France), coupled to a triple quadrupole Agilent 7000D MS (Agilent Technologies, Inc., Santa Clara, CA, USA). The temperature program for the GC oven started at 80 °C (2 min hold), then continued with 15 °C/min to 180 °C (no hold) and lastly 5 °C/min to 310 °C (20 min hold). The inlet temperature was 280 °C. The injection volume was 1 µl in pulsed-splitless mode. The carrier gas was helium with a 1.5 mL/min flow rate. The temperature of the GC-MS transfer line was 310 °C. The ion source was heated to 320 °C. The mass spectrometer was operating in selected ion monitoring (SIM) mode. Compound quantification was done using MassHunter Workstation 10.1 software (Agilent Technologies, Inc., Santa Clara, CA, USA) with an external calibration curve, with a linear range of 1 ng/ml to 1 µg/ml.

Substituted PAHs

PCB 95 was used as the internal standard and added after the final volume reduction. Nitro- and oxy-PAHs were analyzed using an Agilent 7890 GC (Agilent Technologies, Inc., Santa Clara, CA, USA) equipped with a 30m × 0.25mm × 0.25 µm Rxi-5Sil capillary column (Restek, Inc., France), coupled to a Waters Xevo TQ-S MS (Waters Corporation, Milford, MA, USA). The temperature program for the GC oven at 90°C (1 min hold), then increased at a rate of 40 °C/min to 180°C (0 min hold), followed by an increase of 5 °C/min to 320°C (6 min hold). The inlet temperature was set at 270 °C. The injection volume was 1 µl in splitless mode. The MS was operated under dry source conditions in multiple reactions monitoring (MRM) mode. The carrier gas used was helium with a 1.5 mL/min flow rate.

NFRs

PBDE 77 was used as the internal standard and added after the final volume reduction. NFRs were analyzed using Agilent 7890A GC (Agilent Technologies, Inc., Santa Clara, CA, USA) equipped with RTX-1614 size 15 m × 0.25 mm × 0.10 µm column (Restek, Inc., France), coupled to Waters AutoSpec Premier MS (Waters Corporation, Milford, MA, USA). The GC temperature program started at 80 °C (1 min hold), increased at a rate of 30 °C/min to 140 °C (0 min hold), followed by an increase of 4 °C/min to 175 °C (0 min hold), then increase at a rate of 8 °C/min to 270 °C (0 min hold) and finally at 15 °C/min to 325 °C (5 min hold). The injected sample volume was 2 µl at 250 °C in pulsed splitless mode. Helium was used as a carrier gas at 1 mL/min and 1.4 mL/min after 15 min. The MS was operated in EI+ and SIM mode at the resolution of >10000.

PBDEs

PBDE 77 was used as the internal standard and added after the final volume reduction. PBDEs were analyzed using Agilent 7890A GC (Agilent Technologies, Inc., Santa Clara, CA, USA) equipped with RTX-1614 size 15 m × 0.25 mm × 0.10 µm column (Restek, Inc., France), coupled to Waters AutoSpec Premier MS (Waters Corporation, Milford, MA, USA). The GC temperature program started at 80 °C (1 min hold), increased at a rate of 20 °C/min to 250 °C (0 min hold), followed by an increase of 1.5 °C/min to 260 °C (2 min hold) and finally by 25 °C/min to 320 °C (4.5 min hold). The GC/MS interface and ion source temperatures were 280 and 250 °C, respectively. The injected sample volume was 2 µl at 280 °C in pulsed splitless mode. Helium was used as a carrier gas at 1 mL/min and 1.4 mL/min after 15 min. The MS was operated in EI+ and SIM mode at the resolution of >10000. For BDE-209, the resolution was set to >5,000.

PCBs + OCPs

PCB 95 was used as the internal standard and added after final volume reduction. PCBs and OCPs were analyzed using an Agilent 8890A GC (Agilent Technologies, Inc., Santa Clara, CA,

USA) equipped with a 60 m × 0.25 mm × 0.25 µm Rxi-5Sil-MS capillary column (Restek, Inc., France), coupled to a triple quadrupole Agilent 7000D MS (Agilent Technologies, Inc., Santa Clara, CA, USA). The temperature program for the GC oven started at 80 °C (1.5 min hold), then continued with 40 °C/min to 200 °C (18 min hold) and lastly 5 °C/min to 305 °C (no hold). The inlet temperature was 280 °C. The injection volume was 1 µl in pulsed-splitless mode. The carrier gas was helium with a 1.5 mL/min flow rate. The temperature of the GC-MS transfer line was 310 °C. The ion source was heated to 250 °C. The mass spectrometer was operating in multiple reaction monitoring (MRM) mode with nitrogen as collision gas with a flow of 1.5 mL/min. Compound quantification was done with MassHunter Workstation 10.1 software (Agilent Technologies, Inc., Santa Clara, CA, USA) with an external calibration curve, with a linear range of 1 ng/ml to 1 µg/ml.

Phthalates

p-terphenyl was used as the internal standard and added after the final volume reduction. Phthalates were analyzed using an Agilent 7890A GC (Agilent Technologies, Inc., Santa Clara, CA, USA) equipped with a 30 m × 0.25 mm × 0.25 µm Rxi-5Sil-MS capillary column (Restek, Inc., France), coupled to a tandem mass spectrometer Agilent 7000B MS/MS (Agilent Technologies, Inc., Santa Clara, CA, USA). The temperature program of the GC oven started at 80 °C (3 min hold) and then increased at a rate of 7 °C/min until reaching 320 °C (5 min hold). The inlet temperature was set at 280 °C. The injected sample volume was 1 µl in splitless mode. The carrier gas used was helium with a 1.5 mL/min flow rate. The transfer line and ion source temperatures were set at 280 °C. Electron impact ionization (EI) was used, and the mass spectrometer was operating in selected ion monitoring (SIM) mode, with nitrogen as the collision gas at a flow rate of 1.5 mL/min. Compound quantification was done using MassHunter Workstation 10.1 software (Agilent Technologies, Inc., Santa Clara, CA, USA) with an external calibration curve, with a linear range of 10 ng/ml to 10 µg/ml.

CUPs

CUPs were analyzed using an Agilent 1290 Series Gradient HPLC System (Agilent Technologies, Inc., Santa Clara, CA, USA) equipped with a Luna C-18 endcapped (4 µm) size 100 x 2 mm (Phenomenex, CA, USA) equipped with a Phenomenex SecurityGuard C18 size 4 × 2 mm guard column (Phenomenex, CA, USA), coupled to a SCIEX QTrap 5500+ MS (ABSciex, CA, USA) with ESI source. The column was held at 30 °C in a column oven. The injection volume was 5 µl. Separation was achieved using an aqueous solution of 0.1% formic acid (mobile phase A) and MeOH with 0.1% formic acid (mobile phase B) at a flow rate of 0.25 mL/min. The method duration was 11.5 minutes. Ionization was monitored in positive mode using electrospray ionization (ESI+) with the following parameters: 5500 V, a heated source at 400 °C, Ion Source Gas 1 (40 psi), Ion Source Gas 2 (30 psi), and curtain gas (15 psi). An eight-point, calibration curve of native CUP standards was used for quantification and results were processed in Analyst (SCIEX, CA, USA).

PFAS

PFAS were analyzed using an Agilent 1290 Series Gradient HPLC System (Agilent Technologies, Inc., Santa Clara, CA, USA) equipped with a SYNERGI 4µ Fusion Max-RP 80Ä size 100 mm × 2 mm column (Phenomenex, CA, USA) together with a Phenomenex SecurityGuard C18 size 4 × 2 mm guard column (Phenomenex, CA, USA), coupled to a QTrap 5500+ MS (SCIEX, CA, USA) with ESI source. The column was held at 30 °C in a column oven. The injection volume was 10 µl. Separation was achieved using a 5 mM aqueous solution of ammonium acetate (55:45) (mobile phase A) and MeOH (mobile phase B) at a flow rate of 0.4 mL/min. The method duration was 11 minutes. Ionization was monitored in negative mode using electrospray ionization (ESI-) with the following parameters: 4500 V, a heated source at 450 °C, Ion Source Gas 1 (50 psi), Ion Source Gas 2 (30 psi), and curtain gas (15 psi). An

eleven-point, calibration curve of native PFAS standards was used for quantification and results were processed in Analyst (SCIEX, CA, USA).

Text S2 Instrumental analysis at NILU laboratories

PAHs

D10-biphenyl, D10-fluoranthene and D12-perylene were used as the internal standard and added after the final volume reduction. PAHs were analyzed using an Agilent 5977A/7890B GC/LRMS (Agilent Technologies, Inc., Santa Clara, CA, USA) equipped with a standard split/splitless injector. A Select PAH size 30 m × 0.25 mm × 0.15 µm capillary column (Agilent Technologies, Inc., Santa Clara, CA, USA) was used. The temperature program of the GC oven started at 50 °C (1,5 min hold), then the temperature was increased in the first ramp at a rate of 5° C/m to 230 °C (0 min hold), then in the second ramp at a rate of 10 °C/min to 280 °C (0 min hold), and then in the third ramp at a rate of 5 °C/min to 310 °C (10 min hold). The injection volume was 1 µL in splitless mode. The inlet temperature was 300 °C. Helium was used as carrier gas with a 1.9 mL/min flow rate. Electron ionization (EI) SIM mode with a source temperature of 300 °C was used. Molecular ions were used in PAH quantification.

Substituted PAHs

D10-Biphenyl, D10-fluoranthene and D12-perylene were used as the internal standard and added after the final volume reduction. Substituted PAHs were analyzed using an Agilent 7890B/7200 GC/qTOF (Agilent Technologies, Inc., Santa Clara, CA, USA) equipped with a PTV inlet. An HP-5ms Ultra Inert size 15 m × 0.25 mm × 0.25µm capillary column (Agilent Technologies, Inc., Santa Clara, CA, USA) was used. The temperature program of the GC oven started at 55 °C (2 min hold), then the temperature was increased in the first ramp at a rate of 45 °C/min to 150 °C (0 min hold), then in the second ramp at a rate of 5 °C/min to 230°C (0 min hold), and then in the third ramp at a rate of 15 °C/min to 325 °C (3 min hold). The PVT injection volume was 1 µL. The inlet temperature was 300 °C. Helium was used as carrier gas with a flow rate of 1.4 mL/min. ECNI scan mode with a source temperature of 150 °C was used. Molecular ions were used in nitro-oxy-PAH quantification.

PBDEs and NFRs

1,2,3,4-tetrachloronaphthalene was used as the internal standard and added after the final volume reduction. PBDEs and BFRs were analyzed using an Agilent 7890A GC (Agilent Technologies, Inc., Santa Clara, CA, USA) with a 7683B Agilent autosampler (Agilent Technologies, Inc., Santa Clara, CA, USA) equipped with an RTX-1614 size 15 m × 0.25 mm × 0.10 µm capillary column (Restek, Inc., France), coupled to a Micromass Autospec M mass spectrometer (Waters Corporation, Milford, MA, USA). The temperature program of the GC oven started at 45 °C (2.5 min hold), then the temperature was increased in the first ramp at a rate of 22 °C/min until reaching 220 °C (0 min hold) and then in the second ramp at a rate of 7 °C/min until reaching 320 °C (0 min hold). The PTV injection program started at 45 °C (0.35 min hold) then the temperature was increased by 250 °C/min until reaching 320 °C (24 min hold). The injected sample volume was 1 µl. Helium was used as carrier gas with a 1.7 ml/min flow rate.

CPs and dechloranes

1,2,3,4-tetrachloronaphthalene was used as the internal standard and added after final volume reduction. CPs and dechloranes were analyzed using an Agilent 7200B GC-qToF (Agilent Technologies, Inc., Santa Clara, CA, USA) in ECNI mode equipped with an HP-5ms Ultra Inert size 15 m × 0.25 mm × 0.25µm capillary column (Agilent Technologies, Inc., Santa Clara, CA, USA). The temperature program of the GC oven started at 55 °C (2 min hold) and then the temperature was increased in the first ramp at a rate of 70 °C/min until reaching 200 °C (1 min hold), then in the second ramp at the rate of 20 °C/min until reaching 280 °C (1 min hold), then in the third ramp at the rate of 5 °C/min until reaching 300 °C (0 min hold) and finally in the

fourth ramp at the rate of 70 °C/min until reaching 320 °C (1 min hold). The PTV injection program started at 60 °C (0.35 min hold) then the temperature was increased by 500 °C/min until reaching 320 °C (1 min hold), then cooled down at a rate of 500 °C/min until reaching 60 °C (0 min hold). The transfer line temperature (GC-MS connection) was set at 280 °C. The injected sample volume was 0.1 µl. Helium was used as carrier gas with a 1.2 ml/min flow rate.

Phthalates

D4-di-n-propyl phthalate and D4-di-n-octyl phthalate were used as the internal standard and added after the final volume reduction. Phthalates were analyzed using a TSQ Vantage LC-MS/MS (Thermo Fisher Scientific Inc., Waltham, MA, USA) with a HESI source, equipped with an Aquity HSS T3 C18 column size 2.1 mm × 100 mm, 1.8 µm (Waters Corporation, Milford, MA, USA), which was held at 40 °C in a column oven. The injection volume was 10 µl. Separation was achieved using 0.1% formic acid in water (mobile phase A) and 0.1% formic acid in MeOH (mobile phase B) at a flow rate starting at 0.3 to 0.4 mL/min at 100% B. The LC method starts at 90:10 H_2O/MeOH for 30 seconds, ramping fast to 70:30 H_2O/MeOH and slower to 100% MeOH. The method duration was 12 minutes. Ionization was monitored in positive mode using electrospray ionization (ESI+) with the following parameters: 5000 V, a heated source at 380 °C, nitrogen as sheath gas (30 Arb), auxiliary gas (15 Arb), and a capillary temperature of 230 °C. Multiple reaction monitoring (MRM) of the $M + H^+$ was achieved using Argon as the collision gas (1.5 mTorr).

PFAS

3,7-dimethyl PFOA was used as the internal standard and added after the final volume reduction. PFAS were analyzed using a TSQ Vantage UHPLC-MS/MS (Thermo Fisher Scientific Inc., Waltham, MA, USA) coupled to a quaternary Accela 1250 pump (Thermo Fisher Scientific Inc., Waltham, MA, USA) with a PAL Sample Manager (Thermo Fisher Scientific Inc., Waltham, MA, USA). An Acquity UPLC HSS 3 T column size 2.1 mm × 100 mm, 1.8 µm (Waters Corporation, Milford, MA, USA) together with a Waters Van guard HSS T3 size 2.1 mm × 5 mm, 1.8 µm guard column (Waters Corporation, Milford, MA, USA) was used. The injection volume was 10 µL. Separation was achieved using 2 mM NH_4OAc in 90:10 MeOH/water (mobile phase A) and 2 mM NH_4OAc in MeOH (mobile phase B) at a flow rate starting at 0.3 to 0.5 mL/min at 100% B. The method duration was 13.4 minutes. Ionization was monitored in negative mode using electrospray ionization (ESI-) with the following parameters: 2500 V, a heated source at 350 °C, nitrogen as sheath gas (30 Arb), auxiliary gas (10 Arb), and a capillary temperature of 270 °C. The MS was run in SRM mode with Argon as the collision gas (1.5 mTorr) to monitor two product ions for each analyte.

Bisphenols, parabens

D8-4,4'-bisphenol A, D10-4,4'- bisphenol F, D8-bisphenol B and D16-bisphenol P were used as the internal standard and added after the final volume reduction. Bisphenols and parabens were analyzed using a liquid chromatograph Vanquish UHPLC System (Thermo Fisher Scientific Inc., Waltham, MA, USA) equipped with a capillary column Acquity UPLC HSS T3 size 3.0 mm × 150 mm, 1.8 µm (Waters Corporation, Milford, MA, USA), which was held at 40 °C in a column oven, coupled to a Q Exactive Plus Orbitrap mass spectrometer (Thermo Fisher Scientific Inc., Waltham, MA, USA). The injection volume was 1 µl. The mobile phase start gradient was MeOH: Type 1 Water (65:35), increased to 100% MeOH in 15 minutes (7 minutes hold time) at a flow rate of 500 µl/min. The method duration was 22 minutes. The method for MS was Top5 peaks with the following parameters: negative polarity, 113 to 975 m/z scan range and resolution of 70,000. A resolution of 17,500 was used in the subsequent MS^2 stage with an inclusion list and a stepped collision energy (CE) fragmentation of 20, 40, and 60.

HBCDs

D18-α-HBCD, D18-β-HBCD and D18-γ-HBCD were used as the internal standard and added after final volume reduction. HBCDs were analyzed using a liquid chromatograph Vanquish UHPLC System (Thermo Fisher Scientific Inc., Waltham, MA, USA) equipped with a capillary column Acquity UPLC HSS T3 size 3.0 mm × 150 mm, 1.8 μm (Waters Corporation, Milford, MA, USA), which was held at 40 °C in a column oven, coupled to a Q Exactive Plus Orbitrap mass spectrometer (Thermo Fisher Scientific Inc., Waltham, MA, USA). The injection volume was 3 μl. The mobile phase start gradient was 90% MeOH + 10% Acetonitrile: Type 1 Water (5:95), increased to 100% MeOH in 5 minutes (5 minutes hold time) at a flow rate of 450 μl/min. The method duration was 12 minutes. The method for MS was full MS-SIM with the following parameters: negative polarity, 350 – 700 m/z scan range and resolution of 70,000.

BADGE/BFDGE

D10-BADGE was used as the internal standard and added after final volume reduction. BADGE/BFDGE were analyzed using a liquid chromatograph Vanquish UHPLC System (Thermo Fisher Scientific Inc., Waltham, MA, USA) equipped with a capillary column Acquity UPLC HSS T3 size 3.0 mm × 150 mm, 1.8 μm (Waters Corporation, Milford, MA, USA), which was held at 40 °C in a column oven, coupled to a Q Exactive Plus Orbitrap mass spectrometer (Thermo Fisher Scientific Inc., Waltham, MA, USA). The injection volume was 4 μl for samples and 3 μl for standards. The mobile phase start gradient was MeOH: Type 1 Water (40:60) + 2mM Ammoniumacetate, increased to 100% MeOH in 8 minutes (2 minutes hold time) at a flow rate of 500 μl/min. The method duration was 10 minutes. The method for MS was Top5 peaks with the following parameters: positive polarity, 200 – 650 m/z scan range and resolution of 70,000. A resolution of 17,500 was used in the subsequent MS^2 stage with an inclusion list and stepped collision energy (CE) fragmentation of 30.

OPFRs

D27-Tris(2,4-dimethylphenyl) phosphate was used as the internal standard and added after final volume reduction. OPFRs were analyzed using a TSQ Vantage LC-MS/MS (Thermo Fisher Scientific Inc., Waltham, MA, USA) with a HESI source, equipped with an Aquity HSS T3 C18 column size 2.1 mm × 100 mm, 1.8 μm (Waters Corporation, Milford, MA, USA), which was held at 40 °C in a column oven. The injection volume was 10 μl. Separation was achieved using 0.1% formic acid in water (mobile phase A) and 0.1% formic acid in MeOH (mobile phase B) at a flow rate starting at 0.3 to 0.4 mL/min at 100% B. The LC method starts at 90:10 H_2O/MeOH for 30 seconds, ramping fast to 80:20 H_2O/MeOH and slower to 100% MeOH. The method duration was 11 minutes. Ionization was monitored in positive mode using electrospray ionization (ESI+) with the following parameters: 3500 V, a heated source at 280 °C, nitrogen as sheath gas (30 Arb), auxiliary gas (15 Arb), and a capillary temperature of 230 °C. Multiple reaction monitoring (MRM) of the $M + H^+$ was achieved using Argon as the collision gas (1.5 mTorr). Results were processed in LCQuan 2.8 (Thermo Fisher Scientific Inc., Waltham, MA, USA).

Table S4: Instrument detection and quantification limits (IDL, IQL) and blank concentrations for Phase 3 extractions.

	Compound	IDL (ng/sample)	IQL (ng/sample)	SUPRAS blank average ± standard deviation (ng/sample)	MeOH/hex:act blanks average ± stand. dev. (ng/sample)
PAHs	Naphthalene	0.022	0.074	N/A	58.4 ± 3.07
	Biphenyl	0.014	0.043	N/A	14.1 ± 1. 71
	Acenaphthylene	0.034	0.102	N/A	1.30 ± 0.218
	Acenaphthene	0.030	0.089	N/A	< 0.089
	Fluorene	0.033	0.100	< 0.100	8.16 ±1.79
	Phenanthrene	0.024	0.072	< 0.072	9.41 ± 0.129
	Anthracene	0.033	0.100	< 0.100	< 0.100
	Fluoranthene	0.009	0.028	7.86 ± 2.84	6.96 ± 1.58
	Pyrene	0.010	0.029	26.2 ± 9.59	1.84 ± 0.433
	Retene	0.018	0.055	5.54 ± 2.50	1.63 ± 0.419
	Benzo(b)fluorene	0.023	0.069	< 0.069	< 0.069
	Benzo-Naphtho-Thiophene	0.011	0.033	< 0.033	< 0.033
	Benzo(ghi)fluoranthene	0.008	0.023	1.65 ± 0.61	0.425 ± 0.119
	Cyclopenta(cd)pyrene	0.018	0.054	< 0.054	< 0.054
	Benz(a)anthracene	0.008	0.023	< 0.023	< 0.023
	Triphenylene	0.006	0.017	< 0.017	0.464 ± 0.061
	Chrysene	0.006	0.019	< 0.019	0.317 ± 0.027
	Benzo(b)fluoranthene	0.029	0.086	< 0.086	< 0.086
	Benzo(j)fluoranthene	0.030	0.091	< 0.091	< 0.091
	Benzo(k)fluoranthene	0.032	0.096	< 0.096	< 0.096
	Benzo(e)pyrene	0.026	0.079	< 0.079	< 0.079
	Benzo(a)pyrene	0.040	0.119	< 0.119	< 0.119
	Perylene	0.030	0.091	< 0.091	< 0.091
	Indeno(123cd)pyrene	0.025	0.076	< 0.076	< 0.076
	Dibenz(ah)anthracene	0.019	0.058	< 0.058	< 0.058
	Dibenz(ac)anthracene	0.019	0.058	< 0.058	< 0.058
	Benzo(ghi)perylene	0.021	0.062	< 0.062	< 0.062
	Anthanthrene	0.040	0.119	< 0.119	< 0.119
	Coronene	0.032	0.096	< 0.096	< 0.096
PCBs	PCB 9	0.003	0.010	< 0.010	< 0.010
	PCB 11	0.004	0.012	< 0.012	0.057 ± 0.004
	PCB 28	0.005	0.016	< 0.016	< 0.016
	PCB 52	0.005	0.015	< 0.015	< 0.015
	PCB 101	0.010	0.030	< 0.030	< 0.030
	PCB 118	0.009	0.027	< 0.027	< 0.027
	PCB 138	0.011	0.034	< 0.034	< 0.034
	PCB 153	0.010	0.029	< 0.029	< 0.029
	PCB 180	0.008	0.023	< 0.023	< 0.023
OCPs	ppDDT	0.022	0.066	< 0.066	< 0.066
	opDDT	0.021	0.064	< 0.064	< 0.064
	ppDDD	0.012	0.037	< 0.037	< 0.037
	opDDD	0.009	0.028	< 0.028	< 0.028
	ppDDE	0.006	0.019	< 0.019	< 0.019
	opDDE	0.006	0.019	< 0.019	< 0.019
	Alpha-HCH	0.009	0.027	< 0.027	< 0.027
	Beta-HCH	0.031	0.092	< 0.092	< 0.092
	Delta-HCH	0.043	0.128	< 0.128	< 0.128
	Gamma-HCH	0.015	0.045	< 0.045	< 0.045
	Epsilon-HCH	0.017	0.050	< 0.050	< 0.050
	HCB	0.005	0.016	< 0.016	< 0.016
	PeCB	0.003	0.010	< 0.010	< 0.010

	Compound	IDL (ng/sample)	IQL (ng/sample)	SUPRAS blank average ± standard deviation (ng/sample)	MeOH/hex:act blanks average ± stand. dev. (ng/sample)
N-PAHs	1-Nitronaphthalene	0.0003	0.001	0.039 ± 0.055	0.280 ± 0.007
	2-nitronaphthalene	0.005	0.016	< 0.016	0.178 ± 0.124
	3-nitroacenaphthene	0.001	0.003	< 0.003	< 0.003
	5-nitroacenaphthene	0.0001	0.0003	< 0.000	< 0.000
	2-nitrofluorene	0.000	0.001	< 0.001	< 0.001
	9-nitroanthracene	0.027	0.082	< 0.082	< 0.082
	9-nitrophenanthrene	0.009	0.028	< 0.028	< 0.028
	3-nitrophenanthrene	0.121	0.362	< 0.362	< 0.362
	2-nitrofluoranthene	0.001	0.003	< 0.003	0.048 ± 0.009
	3-nitrofluoranthene	0.002	0.005	< 0.005	< 0.005
	1-nitropyrene	0.002	0.006	< 0.006	0.132 ± 0.023
	2-nitropyrene	0.001	0.002	< 0.002	< 0.002
	7-nitrobenzoaanthracene	0.003	0.008	< 0.008	< 0.008
	6-nitrochrysene	0.006	0.019	< 0.019	< 0.019
	1,3-Dinitropyrene	0.270	0.810	< 0.810	< 0.810
	1,6-Dinitropyrene	3.50	10.5	< 10.5	< 10.5
	1,8-Dinitropyrene	1.39	4.17	< 4.17	< 4.17
	6-nitrobenzoapyrene	0.044	0.133	< 0.133	< 0.133
O-PAHs	1,4-naphthoquinone	0.498	1.493	231 ± 174	81.5 ± 25.5
	naphthalene-1-aldehyde	0.184	0.553	< 0.553	0.870 ± 1.13
	9-fluorenone	0.008	0.025	2.97 ± 3.13	5.29 ± 3.12
	9,10-anthraquinone	0.001	0.002	0.07 ± 0.01	4.47 ± 3.15
	benzo-a-fluoren-11-one	0.002	0.006	< 0.006	0.640 ± 0.080
	benzo-b-fluoren-11-one	0.002	0.006	< 0.006	0.478 ± 0.500
	benzanthrone	0.001	0.004	< 0.004	0.460 ± 0.490
	benz(a)anthracene-7,12-dione	0.001	0.002	0.01 ± 0.02	2.47 ± 0.710
	5,12-naphthacenequinone	0.001	0.003	< 0.003	0.070 ± 0.090
PHTHs	DMP	0.370	1.22	< 1.22	1.73 ± 1.40
	DIBP	0.124	0.408	87.5 ± 24.7	137 ± 194
	DEP	0.138	0.457	18.8 ± 2.12	13.1 ± 18.2
	DBP	0.120	0.397	7.21 ± 1.18	44.2 ± 62.2
	DMEP	2.33	7.69	< 7.69	< 7.69
	Bis(4-methyl-2-pentyl)phthalate A	0.481	1.59	< 1.59	< 1.59
	Bis(4-methyl-2-pentyl)phthalate B	0.497	1.64	< 1.64	< 1.64
	Bis(2-ethoxyethyl)phthalate	1.64	5.40	< 5.40	< 5.40
	DPNP	0.131	0.433	< 0.433	< 0.433
	Di-n-hexyl phthalate	0.291	0.962	< 0.962	< 0.962
	BBzP	0.659	2.17	< 2.17	< 2.17
	DBEP	2.76	9.09	< 9.09	< 9.09
	DCHP	0.410	1.35	< 1.35	< 1.35
	DEHP	0.505	1.67	24.0 ± 2.60	27.2 ± 37.1
	DNOP	0.730	2.41	< 2.41	< 2.41
	DINP	2.16	7.14	< 7.14	< 7.14
PFAS	PFBA	0.003	0.010	< 0.010	< 0.010
	PFPA	0.003	0.010	< 0.010	< 0.010
	PFHxA	0.003	0.010	< 0.010	< 0.010
	PFHpA	0.003	0.010	< 0.010	< 0.010
	PFOA	0.006	0.020	< 0.020	< 0.020
	PFNA	0.001	0.004	< 0.004	< 0.004
	PFDA	0.001	0.004	< 0.004	0.021 ± 0.006
	PFUnDA	0.001	0.004	< 0.004	0.005 ± 0.007
	PFDoDA	0.002	0.005	< 0.005	0.010 ± 0.010
	PFTrDA	0.002	0.005	< 0.005	0.009 ± 0.010

	Compound	IDL (ng/sample)	IQL (ng/sample)	SUPRAS blank average ± standard deviation (ng/sample)	MeOH/hex:act blanks average ± stand. dev. (ng/sample)
	PFTeDA	0.001	0.004	< 0.004	0.024 ± 0.009
	PFBS	0.003	0.010	< 0.010	< 0.010
	PFHxS	0.004	0.014	< 0.014	0.006 ± 0.008
	PFHpS	0.002	0.005	< 0.005	< 0.005
	PFOS	0.027	0.090	0.620 ± 0.670	< 0.090
	PFDS	0.001	0.004	< 0.004	0.006 ± 0.008
CUPs	Alachlor	0.030	0.100	< 0.100	< 0.100
	Acetochlor	0.030	0.100	< 0.100	< 0.100
	Atrazin	0.003	0.010	< 0.010	0.213 ± 0.158
	Azinfos-metyl	0.009	0.030	< 0.030	0.076 ± 0.102
	Carbaryl	0.030	0.100	< 0.100	0.302 ± 0.209
	Diazinon	0.003	0.010	< 0.010	< 0.010
	Dimetachlor	0.009	0.030	< 0.030	0.202 ± 0.147
	Dimethoate	0.009	0.030	< 0.030	0.695 ± 0.495
	Disulfoton	0.015	0.050	< 0.050	< 0.050
	Diuron	0.030	0.100	< 0.100	0.268 ± 0.202
	Fenitrothion	0.091	0.300	< 0.300	< 0.300
	Fenoxaprop ethyl	0.003	0.010	< 0.010	< 0.010
	Fenpropimorph	0.003	0.010	< 0.010	0.021 ± 0.028
	Fluroxypyr	0.303	1.00	< 1.00	< 1.00
	Fonofos	0.030	0.100	< 0.100	< 0.100
	Chlorpyrifos	0.003	0.010	< 0.010	< 0.010
	Chlorsulfuron	0.003	0.010	< 0.010	0.751 ± 0.530
	Chlortoluron	0.030	0.100	< 0.100	0.247 ± 0.328
	Isoproturon	0.003	0.010	< 0.010	0.337 ± 0.233
	Malathion	0.030	0.100	< 0.100	< 0.100
	Metamitron	0.076	0.250	< 0.250	1.04 ± 0.719
	Metazachlor	0.009	0.030	< 0.030	0.308 ± 0.217
	Metolachlor	0.003	0.010	< 0.010	< 0.010
	Metribuzin	0.009	0.030	< 0.030	0.344 ± 0.242
	Methyl Parathion	0.152	0.500	< 0.500	< 0.500
	Pendimethalin	0.003	0.010	< 0.010	< 0.010
	Pirimicarb	0.009	0.030	< 0.030	0.248 ± 0.177
	Prochloraz	0.003	0.010	< 0.010	< 0.010
	Propiconazole	0.009	0.030	< 0.030	< 0.030
	Pyrazon	0.018	0.060	< 0.060	0.863 ± 0.612
	Simazine	0.003	0.010	< 0.010	0.381 ± 0.270
	Tebuconazole	0.003	0.010	< 0.010	< 0.010
	Temefos	0.009	0.030	< 0.030	< 0.030
	Terbufos	0.015	0.050	< 0.050	< 0.050
	Terbuthylazine	0.003	0.010	< 0.010	0.044 ± 0.064

Table S5: Percentage recoveries from Phase 1 analysis, SUPRAS 1-5 are different SUPRAS (see Table 1), and A and B are replicates.

		SUPRAS 1		SUPRAS 2		SUPRAS 3		SUPRAS 4		SUPRAS 5		BLANKS				
	**Compound**	**A**	**B**	**A**	**B**	**A**	**B**	**A**	**B**	**A**	**B**	**SUPRAS 1**	**SUPRAS 2**	**SUPRAS 3**	**SUPRAS 4**	**SUPRAS 5**
PAHs	Phenanthrene-D10	35.8	39.7	38.3	20.8	30.3	34.8	41.5	40.8	28.6	29.2	33.5	9.22	51.8	11.1	2.95
	Perylene-D12	70.0	67.6	71.1	61.0	58.7	63.4	70.9	71.7	53.0	54.6	90.9	92.1	76.3	61.5	37.8
Nitro-PAHs	1-nitronaphthalene-D7	32.8	42.6	27.4	5.15	16.1	21.5	26.5	29.8	16.5	21.8	4.96	0.212	18.1	0.535	0.032
	2-nitrofluorene-D9	46.4	61.5	40.6	27.1	29.0	39.5	38.8	48.7	32.6	36.7	29.5	24.7	24.6	15.8	8.27
	9-nitroanthracene-D9	28.0	20.3	17.5	11.5	10.3	12.0	17.1	17.8	10.7	10.8	8.60	9.34	8.91	9.09	4.19
	3-nitrofluoranthene-D9	70.1	83.0	71.8	41.8	41.0	60.1	67.6	70.0	46.0	61.8	31.6	32.6	23.8	18.4	9.34
	1-nitropyrene-D9	80.5	90.1	79.6	46.5	46.2	65.8	73.6	72.2	50.2	60.7	27.2	29.9	21.3	16.6	7.67
	6-nitrochrysene-D11	99.6	169	131	78.4	78.5	116	134	166	104	129	33.6	37.5	23.9	17.2	9.04
	6-nitrobenzoapyrene-D11	76.5	68.7	121	67.4	54.3	108	181	244	128	156	0.392	0.217	0.200	0.820	0.142
Oxy-PAHs	Dibenzofuran-d8	79.6	112	69.3	16.1	45.5	52.7	84.3	80.3	36.6	65.3	3.78	0.764	33.2	0.158	0.529
	9-fluorenone-d8	91.6	101	62.9	28.6	46.3	62.8	69.5	82.1	47.2	52.8	41.5	12.5	64.1	14.6	3.35
	9,10-anthraquinone-d8	145	171	110	61.4	61.0	81.7	88.7	90.9	61.3	72.4	145	119	113	70.4	33.4
Musk	Tonalide-D3	73.4	68.3	73.0	61.3	80.1	72.0	72.3	65.0	57.0	49.2	95.6	67.5	89.7	46.8	24.5
NFRs	13-C-PBDE 28	N/A	12.2	109	8.60	63.2	66.6	72.3	19.2	90.1	13.3	10.1	84.9	31.6	5.30	39.4
	13C-PBBZ	N/A	52.6	92.3	52.5	39.5	44.6	55.3	45.3	122	39.3	36.5	70.8	46.8	19.3	24.6
	13C_HBB	N/A	85.2	75.5	51.4	56.4	45.8	73.4	62.4	77.8	106	88.6	63.1	46.4	47.0	43.7
	13-C-PBDE 47	N/A	69.6	103	44.6	66.8	70.1	68.6	51.8	95.8	95.7	83.4	94.7	48.0	47.3	48.4
	13-C-EH-TBB	N/A	99.1	118	63.9	80.5	52.9	94.2	52.7	78.8	82.5	71.9	71.3	47.2	38.4	42.3
	13-C-BTBPE	N/A	118	121	86.4	83.9	42.5	99.6	57.8	ND	93.6	67.3	ND	41.7	ND	ND
PCBs	13C-PCB 28	78.6	82.8	76.4	55.0	47.3	67.0	60.2	82.5	45.0	48.4	77.1	47.2	62.7	32.5	13.1
	13C-PCB 52	73.1	80.7	74.7	70.2	58.3	70.7	62.0	76.1	54.9	86.5	84.4	56.1	71.6	39.7	18.2
	13C-PCB 101	82.1	85.8	53.5	56.1	46.3	85.3	68.9	95.7	38.0	91.7	83.3	80.3	65.3	53.0	26.1
	13C-PCB 138	87.1	103	84.4	109	78.7	52.3	83.8	62.4	41.3	64.1	97.2	92.7	65.9	64.5	30.4
	13C-PCB 153	94.3	87.0	58.4	106	46.5	52.8	50.7	86.9	65.1	84.6	90.1	102	78.0	61.6	30.4
	13C-PCB 180	92.6	59.3	95.5	102	80.1	50.9	66.8	93.9	63.5	99.7	87.0	97.8	49.2	67.2	35.1
OCPs	13C-PeCB	19.7	32.6	21.1	1.53	17.1	17.0	28.5	30.2	11.9	29.9	2.21	0.859	16.5	ND	ND
	13C-HCB	49.3	59.1	47.5	17.9	39.5	41.1	44.5	54.9	29.5	53.2	27.4	7.15	43.1	4.34	ND
	13C-b-HCH	77.6	74.3	64.6	64.3	54.6	69.6	66.4	71.6	46.3	75.1	78.5	59.7	62.4	38.2	20.5
	13C-g-HCH	71.6	69.5	57.7	44.8	45.1	58.0	59.7	61.7	44.0	62.7	55.1	19.6	58.9	18.0	5.03
	13C-ppDDT	94.7	100	78.2	97.3	61.9	41.5	62.2	55.9	34.3	52.7	119	108	82.1	67.0	30.2

		SUPRAS 1		SUPRAS 2		SUPRAS 3		SUPRAS 4		SUPRAS 5		BLANKS				
	**Compound**	**A**	**B**	**A**	**B**	**A**	**B**	**A**	**B**	**A**	**B**	**SUPRAS 1**	**SUPRAS 2**	**SUPRAS 3**	**SUPRAS 4**	**SUPRAS 5**
	13C-ppDDD	107	77.4	103	120	56.0	87.7	58.6	101	77.8	122	89.4	85.3	69.2	55.3	25.8
	13C-ppDDE	77.0	85.8	82.3	91.0	74.5	76.0	64.8	90.3	62.4	90.7	86.1	86.5	70.0	57.8	28.7
PHTHs	D4-DEP	37.7	51.6	40.2	14.0	37.4	41.5	47.1	29.2	30.2	35.8	43.8	10.9	61.5	18.9	5.81
	D4-BBzP	87.8	106	89.8	82.8	83.3	102	97.8	61.1	74.7	75.8	105	104	91.8	67.8	36.8
	D4-DEHP	91.0	114	96.9	95.8	87.6	102	108	66.6	74.4	74.3	108	112	97.2	70.0	41.1
CUPs	Acetochlor-D11	32.6	31.7	30.7	N/A	33.3	36.5	35.0	35.4	24.5	24.4	80.3	88.8	86.5	60.8	44.2
	Alachlor-D13	29.4	33.4	30.6	N/A	35.2	38.0	38.5	36.0	25.4	23.3	93.4	102	101	70.2	46.8
	Atrazine-D5	49.3	55.9	48.5	N/A	53.6	54.3	60.5	48.1	41.9	35.6	82.3	84.2	86.2	86.9	37.8
	Carbendazim-D4	60.6	59.6	47.1	N/A	47.6	56.7	60.6	57.4	35.7	30.3	93.4	83.8	92.9	90.2	46.1
	Chlorpyrifos-D10	2.98	2.64	2.66	N/A	3.79	3.30	2.68	2.77	1.88	1.82	62.6	66.5	72.7	42.8	34.3
	Chlortoluron-D6	45.6	44.8	39.9	N/A	41.3	45.6	40.7	46.4	33.4	22.7	91.6	78.6	85.3	97.9	45.2
	Dimethoate-D6	41.1	48.7	40.3	N/A	33.2	33.5	43.9	43.3	30.4	26.1	83.5	75.7	87.2	63.0	46.1
	Diuron-D6	31.8	35.0	28.4	N/A	36.2	37.9	42.6	33.8	28.8	24.8	111	89.1	108	119	56.2
	Fenitrothion-D6	59.2	43.3	31.8	N/A	60.3	46.9	62.5	29.4	39.7	62.7	71.3	61.9	94.5	45.1	49.4
	Isoproturon-D6	45.6	46.4	39.6	N/A	41.6	45.1	48.1	42.6	30.5	30.7	103	92.4	96.2	79.7	51.9
	Metamitron-D5	26.3	31.9	29.2	N/A	23.7	23.9	31.9	33.9	24.6	26.1	79.8	73.5	71.8	78.8	36.8
	Metazachlor-D6	50.2	45.3	37.5	N/A	50.9	56.6	44.2	46.8	40.0	32.9	107	90.6	89.8	106	46.0
	Metolachlor-D6	41.7	51.8	47.5	N/A	43.8	53.5	54.6	52.0	37.1	33.7	132	104	137	121	65.8
	Metribuzin-D3	33.0	41.7	32.8	N/A	37.1	38.5	37.4	50.1	25.2	28.9	98.6	94.7	83.7	100	48.7
	Pendimethalin-D5	6.65	10.3	10.6	N/A	8.06	11.4	11.3	9.24	9.20	8.06	68.9	66.9	83.0	48.9	42.3
	Prochloraz-D7	36.8	35.5	34.3	N/A	34.4	41.6	40.1	38.5	27.3	26.2	103	89.6	102	103	41.6
	Propiconazole-D5	32.5	36.6	29.5	N/A	34.4	37.8	37.8	32.7	23.7	19.1	99.6	85.1	92.0	106	43.6
	Pyrazon-D5	37.8	37.8	27.0	N/A	28.7	29.8	37.2	33.6	23.6	25.1	86.0	78.0	89.7	103	51.2
	Simazine-D10	68.0	72.1	56.1	N/A	63.1	63.5	68.1	66.6	33.1	39.6	102	93.2	95.8	86.4	39.8
	Tebuconazole-D6	37.7	36.9	40.5	N/A	39.0	40.0	39.5	40.3	26.5	29.4	88.9	85.7	89.4	100	42.6
	Terbuthylazine-D5	45.1	45.1	44.8	N/A	45.4	45.7	46.3	43.6	32.2	25.4	90.0	72.4	89.1	81.1	43.3
PFAS	Perfluoro-n-[1,2-13C2]dodecanoic acid (MPFDoDA)	40.7	44.2	40.3	N/A	42.8	41.7	42.6	44.7	37.4	36.0	177	151	174	181	86.8
	Perfluoro-n-[1,2,3,4-13C4]butanoic acid (MPFBA)	36.2	45.3	48.1	N/A	31.8	28.3	40.6	39.3	56.6	37.1	98.4	101	87.4	110	62.3
	Perfluoro-n-[1,2-13C2]decanoic acid (MPFDA)	32.0	34.9	28.8	N/A	34.1	31.2	33.8	36.7	26.6	24.7	152	124	142	135	75.1
	Perfluoro-n-[1,2-13C2]hexanoic acid (MPFHxA)	27.2	38.1	40.7	N/A	31.0	27.8	29.9	28.2	42.4	42.9	91.8	100	82.7	94.1	61.1
	Sodium perfluoro-1-[18O2]hexanesulfonate (MPFHxS)	48.4	64.1	57.2	N/A	43.4	48.6	55.1	56.2	60.9	59.9	133	118	130	133	64.1
	Perfluoro-n-[1,2,3,4,5,-13C5]nonanoic acid (MPFNA)	52.5	63.9	58.3	N/A	55.7	56.2	56.7	61.5	60.3	55.1	166	146	159	146	78.3
	Perfluoro-n-[1,2,3,4-13C4]octanoic acid (MPFOA)	47.0	61.1	55.6	N/A	48.4	51.0	48.7	49.0	52.7	52.1	130	122	125	124	68.0
	Sodium perfluoro-1-[1,2,3,4-13C4]-octanesulfonate (MPFOS)	57.3	74.8	64.1	N/A	54.3	58.7	68.4	67.8	68.8	62.7	154	131	137	160	65.1

		SUPRAS 1		SUPRAS 2		SUPRAS 3		SUPRAS 4		SUPRAS 5		BLANKS				
	**Compound**	**A**	**B**	**A**	**B**	**A**	**B**	**A**	**B**	**A**	**B**	**SUPRAS 1**	**SUPRAS 2**	**SUPRAS 3**	**SUPRAS 4**	**SUPRAS 5**
	Perfluoro-n-[1,2,-13C2]undecanoic acid (MPFUnDA)	38.3	42.2	36.1	N/A	38.9	37.5	40.0	42.7	33.4	32.8	169	147	162	163	83.6
	Perfluoro-n-[1,2,3,4-13C4]butanoic acid (MPFBA)	6.22	5.42	13.5	N/A	4.39	4.28	5.79	3.99	27.9	29.9	8.01	10.8	4.68	41.1	34.2

*Table S6: Matrix effect from Phase 1 analysis calculated for SUPRAS 1. The matrix effect was calculated as 100-[(average response of analyte in matrix eluent/average response of analyte in standard solution)*100].*

Group	Compound	Matrix Effect (%)
PAHs	Phenanthrene-D10	-12.7
	Perylene-D12	24.3
Nitro-PAHs	1-nitronaphthalene-D7	-661
	2-nitrofluorene-D9	-83.0
	9-nitroanthracene-D9	-181
	3-nitrofluoranthene-D9	-142
	1-nitropyrene-D9	-214
	6-nitrochrysene-D11	-300
	6-nitrobenzoapyrene-D11	-18400
Oxy-PAHs	Dibenzofuran-d8	-2310
	9-fluorenone-d8	-132
	9,10-anthraquinone-d8	-9.07
Musk	Tonalide-D3	25.9
NFRs	13-C-PBDE 28	-20.8
	13C-PBBZ	-44.1
	13C_HBB	3.84
	13-C-PBDE 47	16.5
	13-C-EHTBB	-37.8
	13-C-BTBPE	-75.2
PCBs	13C-PCB 28	-4.73
	13C-PCB 52	8.94
	13C-PCB 101	-0.80
	13C-PCB 138	2.44
	13C-PCB 153	-0.58
	13C-PCB 180	12.7
OCPs	13C-PeCB	-1080
	13C-HCB	-97.8
	13C-b-HCH	3.22
	13C-g-HCH	-28.1
	13C-ppDDT	18.0
	13C-ppDDD	-3.45
	13C-ppDDE	5.49
PHTH	D4-DEP	-1.94
	D4-BBzP	8.07
	D4-DEHP	5.40
CUPs	Acetochlor-D11	60.0
	Alachlor-D13	66.4
	Atrazine-D5	36.1
	Carbendazim-D4	35.6
	Chlorpyrifos-D10	95.5
	Chlortoluron-D6	50.7
	Dimethoate-D6	46.2
	Diuron-D6	69.9
	Fenitrothion-D6	28.1
	Isoproturon-D6	55.5
	Metamitron-D5	63.6
	Metazachlor-D6	55.5
	Metolachlor-D6	64.6
	Metribuzin-D3	62.2
	Pendimethalin-D5	87.7
	Prochloraz-D7	65.0
	Propiconazole-D5	65.3
	Pyrazon-D5	56.1
	Simazine-D10	31.2
	Tebuconazole-D6	58.0
	Terbuthylazine-D5	49.8
PFAS	MPFDoDA	75.9
	MPFBA	58.6

	MPFDA	77.9
	MPFHxA	64.4
	MPFHxS	57.7
	MPFNA	64.9
	MPFOA	58.7
	MPFOS	57.0
	MPFUnDA	76.1
	MPFBA	27.4

Table S7: Percentage recoveries for Phase 2 analysis. A, B, an C are 3 replicates.

	Compound	A	B	C	Average	St. Dev.
PAHs	D10-Acenaften	7.80	12.7	11.8	10.7	2.13
	D10-Antracene	32.7	30.5	36.2	33.1	2.35
	10D-Pyrene	44.1	42.5	48.5	45.0	2.54
	D12-Benz(a)anthracene	45.4	38.2	46.9	43.5	3.80
	D12-Benz(e)pyrene	49.3	43.8	51.6	48.2	3.27
	D12-Benzo(ghi)perylene	43.3	41.0	46.8	43.7	2.38
Nitro-PAHs	D9-1-Nitropyrene	62.1	48.9	42.6	51.2	8.11
	D9-3-Nitrofluorathene	73.9	58.6	49.3	60.6	10.1
PBDEs + NFRs	13C-PBDE 28	92.8	109	109	104	7.57
	13C-PBBZ	60.7	73.1	84.7	72.8	9.80
	13C-HBB	61.6	74.9	82.9	73.1	8.78
	13C-PBDE 47	109	139	137	128	13.7
	13C-EH-TBB	85.1	110	111	102	11.9
	13C-BT-BEP	71.5	100	104	92.1	14.6
PHTHs	D4-DEP	103	117	159	126	23.6
	D4-DIBP	35.0	33.1	52.1	40.0	8.53
	D4-DHxP	42.5	40.7	62.3	48.5	9.78
	D4-DEHP	86.2	85.1	111	94.1	11.9
CUPs	D11-Acetochlor	9.51	12.8	11.2	11.2	1.35
	D13-Alachlor	11.3	18.4	15.4	15.1	2.91
	D5-Atrazine	16.1	22.5	15.4	18.0	3.21
	D4-Carbendazim	15.1	20.2	17.3	17.5	2.07
	D10-Chlorpyrifos	4.28	5.45	4.13	4.62	0.589
	D6-Chlortoluron	16.7	20.7	14.2	17.2	2.66
	D6-Dimethoate	19.5	24.1	20.8	21.5	1.93
	D6-Diuron	14.3	16.9	13.6	15.0	1.41
	D6-Fenitrothion	6.98	10.2	6.69	7.95	1.59
	D6-Isoproturon	14.3	16.9	13.6	15.0	1.41
	D6-Metazachlor	19.9	26.9	18.3	21.7	3.71
	D6-Metolachlor	13.5	19.3	16.8	16.5	2.41
	D3-Metribuzin	15.6	21.9	22.2	19.9	3.05
	D5-Pendimethalin	16.2	26.8	19.2	20.8	4.47
	D7-Prochloraz	9.30	14.2	9.40	11.0	2.28
	D5-Propiconazole	9.34	14.1	9.86	11.1	2.12
	D10-Simazine	18.7	25.8	21.9	22.1	2.90
	D6-Tebuconazole	15.1	24.2	16.2	18.5	4.06
	D5-Terbuthylazin	8.51	12.1	9.55	10.0	1.50
PFAS	13C-PFPA			39.7	39.7	
	13C-PFHxA		60.7	44.6	52.7	8.06
	13C-PFHpA		52.3	38.2	45.3	7.04
	13C-PFOA		67.3	52.9	60.1	7.22
	13C-PFNA		69.3	64.6	66.9	2.34
	13C-PFDA		57.7	45.9	51.8	5.90

	Compound	A	B	C	Average	St. Dev.
	13C-PFUnDA		60.3	39.8	50.0	10.2
	13C-PFDoDA		64.6	45.2	54.9	9.69
	13C-PFTeDA		67.7	50.7	59.2	8.51
	13C-PFBS		76.7	63.6	70.1	6.52
	13C-PFHxS		71.0	55.7	63.4	7.66
	13C-PFOS		71.0	63.7	67.4	3.64
	13C-FOSA		11.3	4.46	7.86	3.41
	13C-6:2 FTS		186	147	166	19.3
	13C-8:2 FTS		139	108	124	15.2
Dechlorane	13C-Dechlorane 602	43.1	46.1	43.5	44.2	1.33
	13C-Dechlorane plus syn	53.4	58.1	54.5	55.3	2.01
CPs	13C-Hexachlorodecane	57.0	66.0	60.0	61.0	3.74
OPFRs	D15-TEP	21.8	13.9	30.3	22.0	6.71
	D12-TCEP	79.1	78.5	84.9	80.9	2.88
	D18-TCPP	65.5	60.2	59.5	61.7	2.68
	D15-TDCPP	131	107	131	123	11.6
	D15-TPP	80.4	68.7	69.5	72.9	5.34
	D27-TnBP	65.5	60.6	62.1	62.7	2.05
	D33-TIPPP	61.9	67.6	62.5	64.0	2.58
	D51-TEHP	96.6	107	84.9	96.1	8.98
Bisphenols	13C-bisphenol A	24.2	54.9	10.3	29.8	18.6
	13C-4,4-bisphenol S	72.8	31.8	33.0	45.9	19.1
	13C-4,4-bisphenol F	44.0	74.5	12.9	43.8	25.2
	13C-bisphenol B	32.5	42.2	9.78	28.2	13.6
	13C-2,4-bisphenol S	93.1	50.5	57.0	66.9	18.7
	13C-bisphenol AF	28.5	32.4	7.99	23.0	10.7
	D4-2,2-bisphenol F	27.2	70.5	7.52	35.1	26.3
BADGE	D6-BADGE	25.0	62.5	8.25	31.9	22.7
	13C-BFDGE	60.4	63.9	16.0	46.8	21.8
Parabens	13C-Butyl-4-Hydroxybenzoate	14.8	43.3		29.1	14.3
	13C-Ethyl-4-Hydroxybenzoate	9.61	16.1		12.9	3.36
	13C-Methyl-4-Hydroxybenzoate	7.51	13.2		10.4	2.84
	13C-Propyl-4-Hydroxybenzoate	2.74	6.81		4.78	2.03

Table S8: Comparison of SRM 2585 certified/literature values (ng/g) with those obtained by SUPRAS extraction and "conventional" hex:ace and MeOH extractions.

	Compound	SRM 2585 certified	SRM SUPRAS extraction	SRM hex:acet/MeOH extraction
PAHs	Naphthalene	266		
	Phenanthrene	1920	1340	1240
	Anthracene	96	178	132
	Fluoranthene	4380	4210	9150
	Pyrene	3290	3140	7030
	Benz[a]anthracene	1160	1090	975
	Chrysene	2260	1950	1800
	Benzo[b]fluoranthene	2700	3720	8020
	Benzo[k]fluoranthene	1330	1280	1200
	Benzo[a]pyrene	1140	1060	1022
	Indeno[1,2,3-cd]pyrene	2080	2750	2800
	Dibenz[a,h]anthracene	301	296	265
	Benzo[ghi]perylene	2280	2800	2810
	Benzo[ghi]fluoranthene	317	366	781
	Triphenylene	589	464	440
	Benzo[j]fluoranthene	1320	1000	925
	Benzo[e]pyrene	2160	2090	1910
	Perylene	387	315	287
	Dibenz[a,c]anthracene	183	253	252
	Coronene	603	1220	1370
PCBs	PCB 28	13.4	9.47	8.02
	PCB 52	21.8	14.6	12.9
	PCB 101	29.8	23.5	20.5
	PCB 118	26.3	23.4	18.9
	PCB 138	27.6	24.5	20.5
	PCB 153	40.2	25.6	23.9
	PCB 180	18.4	17.5	20.7
OCPs	ppDDT	111	59.9	35.3
	opDDT	44.5	35.8	23.8
	ppDDD	27.3	16.5	5.32
	ppDDE	261	180	155
	g-HCH	4.1		2.56
	PeCB	20.9	163	21.2
PFAS	PFBA	230	277	229
	PFHxA	260	429	366
	PFHpA	249	295	316
	PFNA	99.4	105	94.9
	PFDoA	34.6	36.5	36.0
	PFTriA	29.4	20.9	21.8
	PFHxS	1440	1250	1260
	PFOS	2310	2260	2360
	PFOA	567.0	928	757
	PFDA	38.1	67.2	67.4
	PFUnA	37.8	43.6	42.7
	PFBS	40.8	18.0	18.1
PBDEs	PBDE 28	46.9	48.0	48.1
	PBDE 47	497	617	571
	PBDE 85	43.8	42.4	32.4
	PBDE 99	892	1100	955
	PBDE 100	145	183	163
	PBDE 153	119	159	141
	PBDE 154	83.5	102	93.7
	PBDE 183	43.0	107	57.4
	PBDE 209	2510	N/A	2940
NF	BTBPE	38.4	22.3	28.5

	Compound	SRM 2585 certified	SRM SUPRAS extraction	SRM hex:acet/MeOH extraction
	EH-TBB	32.4	26.1	32.5
	BEH-TEBP	574	1020	1430
	TBP-AE	6	0.308	0.196
	PBBZ	1.6	2.67	2.43
	PBEB	7.7	6.93	7.32
	HBB	2.8	3.13	2.70
	Syn-DP	18.7	18.6	52.0
	Anti-DP	44.1	18.1	30.9
PHTHs	DMP	1.8	1.65	1.10
	DEP	7.5	11.1	8.03
	DiBP	6.5	11.9	13.4
	DnBP	32.4	28.3	30.6
	BzBP	93.0	92.4	95.5
	DEHP	552.0	484	508
	DiNP	199.0	66.6	81.7
Musk	Galaxolide	1470	1200	2450
	Tonalide	1700	1036	2470

Reiner, J.L., Blaine, A.C., Higgins, C.P. et al. Polyfluorinated substances in abiotic standard reference materials. Anal Bioanal Chem 407, 2975–2983 (2015). https://doi.org/10.1007/s00216-013-7330-2

Fan X., Kubwabo C., Rasmussen P.E., Wu F. Non-PBDE halogenated flame retardants in Canadian indoor house dust: sampling, analysis, and occurrence. Environmental Science and Pollution Research 23:7998–8007 (2016). https://doi.org/10.1007/s11356-015-5956-7

Larsson K., Lindh C.H., Jönsson B.A., et al. Phthalates, non-phthalate plasticizers and bisphenols in Swedish preschool dust in relation to children’s exposure. Environ Int 102:114–124 (2017). https://doi.org/10.1016/j.envint.2017.02.006

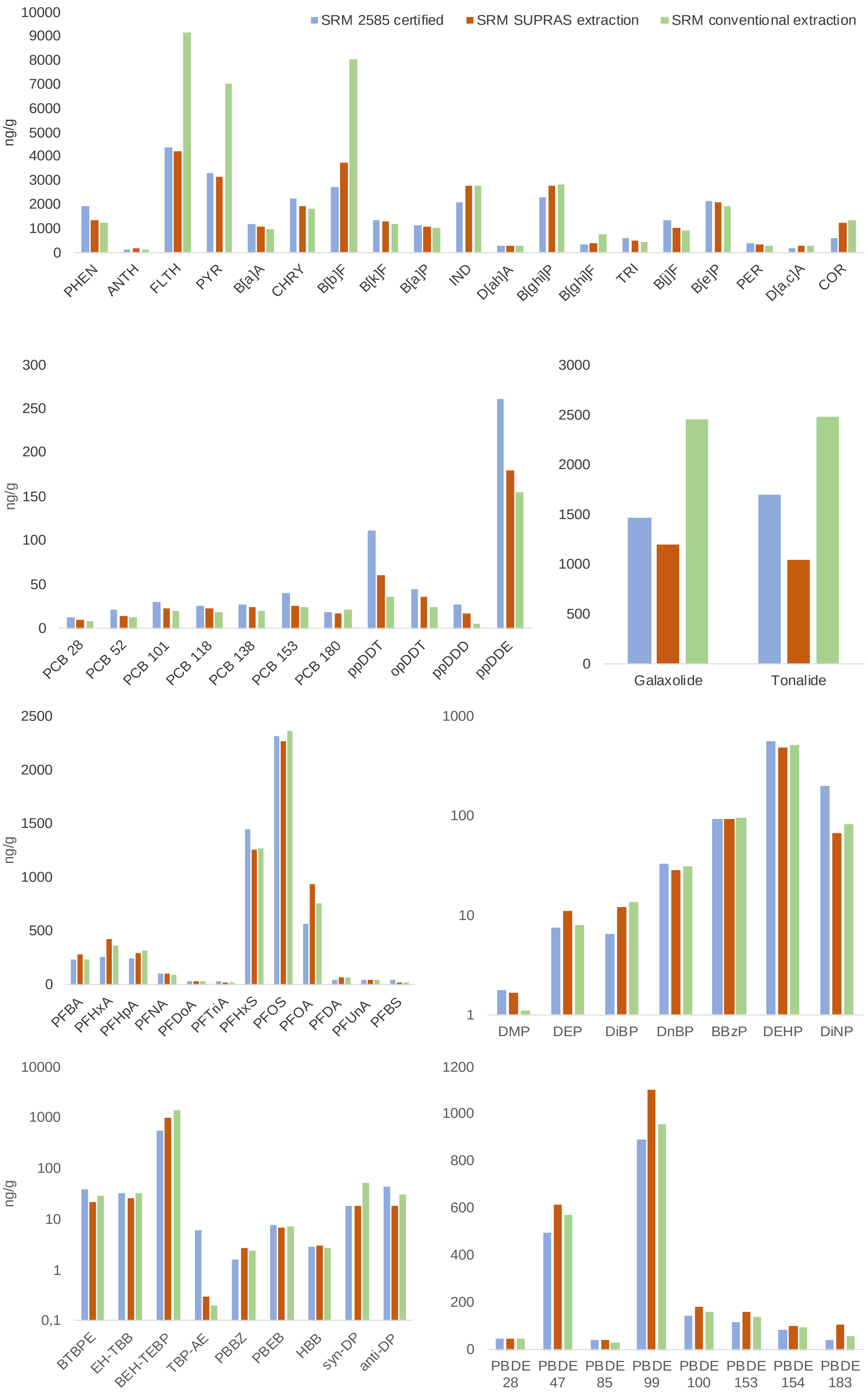

Figure S1: Comparison of SRM 2585 certified/literature values (ng/g) with those obtained by SUPRAS extraction and "conventional" extractions. PHTHs and NFRs are displayed on logarithmic scale.
